# COVID-19 bacteremic co-infection is a major risk factor for mortality, ICU admission, and mechanical ventilation

**DOI:** 10.1186/s13054-023-04312-0

**Published:** 2023-01-23

**Authors:** Michael John Patton, Carlos J. Orihuela, Kevin S. Harrod, Mohammad A. N. Bhuiyan, Paari Dominic, Christopher G. Kevil, Daniel Fort, Vincent X. Liu, Maha Farhat, Jonathan L. Koff, Charitharth V. Lal, Anuj Gaggar, Robert P. Richter, Nathaniel Erdmann, Matthew Might, Amit Gaggar

**Affiliations:** 1 Medical Scientist Training Program, Heersink School of Medicine, University of Alabama at Birmingham, Birmingham, AL USA; 2 Hugh Kaul Precision Medicine Institute, University of Alabama at Birmingham, Birmingham, AL USA; 3 Department of Microbiology, University of Alabama at Birmingham, Birmingham, AL USA; 4 Department of Anesthesiology and Perioperative Medicine, Heersink School of Medicine, University of Alabama at Birmingham, Birmingham, AL USA; 5 Department of Internal Medicine, Division of Clinical Informatics, Louisiana State University Health Sciences Center at Shreveport, Shreveport, LA USA; 6 Department of Medicine, Division of Cardiovascular Sciences, University of Iowa, Iowa City, IA USA; 7 Departments of Pathology, Molecular and Cellular Physiology, and Cellular Biology and Anatomy, Louisiana State University Health Sciences Center at Shreveport, Shreveport, LA USA; 8 Ochsner Health System, New Orleans, LA USA; 9 Kaiser Permanente Division of Research, Oakland, CA USA; 10 Harvard University Medical School, Boston, MA USA; 11 Department of Medicine, Division of Pulmonary, Critical Care, and Sleep Medicine, Yale University, New Haven, USA; 12 Department of Pediatrics, Neonatology Division, University of Alabama at Birmingham, Birmingham, AL USA; 13 ArriveBio, San Francisco, CA USA; 14 Department of Pediatrics, Division of Pediatric Critical Care, University of Alabama at Birmingham, Birmingham, AL USA; 15 Department of Medicine, Division of Infectious Diseases, University of Alabama at Birmingham, Birmingham, AL USA; 16 Department of Medicine, Pulmonary, Allergy, and Critical Care Medicine Division, University of Alabama at Birmingham, Birmingham, AL USA; 17 Birmingham VA Medical Center, Pulmonary Section, Birmingham, AL USA

## Abstract

**Background:**

Recent single-center reports have suggested that community-acquired bacteremic co-infection in the context of Coronavirus disease 2019 (COVID-19) may be an important driver of mortality; however, these reports have not been validated with a multicenter, demographically diverse, cohort study with data spanning the pandemic.

**Methods:**

In this multicenter, retrospective cohort study, inpatient encounters were assessed for COVID-19 with community-acquired bacteremic co-infection using 48-h post-admission blood cultures and grouped by: (1) confirmed co-infection [recovery of bacterial pathogen], (2) suspected co-infection [negative culture with ≥ 2 antimicrobials administered], and (3) no evidence of co-infection [no culture]. The primary outcomes were in-hospital mortality, ICU admission, and mechanical ventilation. COVID-19 bacterial co-infection risk factors and impact on primary outcomes were determined using multivariate logistic regressions and expressed as adjusted odds ratios with 95% confidence intervals (Cohort, OR 95% CI, Wald test *p* value).

**Results:**

The studied cohorts included 13,781 COVID-19 inpatient encounters from 2020 to 2022 in the University of Alabama at Birmingham (UAB, *n* = 4075) and Ochsner Louisiana State University Health—Shreveport (OLHS, *n* = 9706) cohorts with confirmed (2.5%), suspected (46%), or no community-acquired bacterial co-infection (51.5%) and a comparison cohort consisting of 99,170 inpatient encounters from 2010 to 2019 (UAB pre-COVID-19 pandemic cohort). Significantly increased likelihood of COVID-19 bacterial co-infection was observed in patients with elevated ≥ 15 neutrophil-to-lymphocyte ratio (UAB: 1.95 [1.21–3.07]; OLHS: 3.65 [2.66–5.05], *p* < 0.001 for both) within 48-h of hospital admission. Bacterial co-infection was found to confer the greatest increased risk for in-hospital mortality (UAB: 3.07 [2.42–5.46]; OLHS: 4.05 [2.29–6.97], *p* < 0.001 for both), ICU admission (UAB: 4.47 [2.87–7.09], OLHS: 2.65 [2.00–3.48], *p* < 0.001 for both), and mechanical ventilation (UAB: 3.84 [2.21–6.12]; OLHS: 2.75 [1.87–3.92], *p* < 0.001 for both) across both cohorts, as compared to other risk factors for severe disease. Observed mortality in COVID-19 bacterial co-infection (24%) dramatically exceeds the mortality rate associated with community-acquired bacteremia in pre-COVID-19 pandemic inpatients (5.9%) and was consistent across alpha, delta, and omicron SARS-CoV-2 variants.

**Conclusions:**

Elevated neutrophil-to-lymphocyte ratio is a prognostic indicator of COVID-19 bacterial co-infection within 48-h of admission. Community-acquired bacterial co-infection, as defined by blood culture-positive results, confers greater increased risk of in-hospital mortality, ICU admission, and mechanical ventilation than previously described risk factors (advanced age, select comorbidities, male sex) for COVID-19 mortality, and is independent of SARS-CoV-2 variant.

**Supplementary Information:**

The online version contains supplementary material available at 10.1186/s13054-023-04312-0.

## Introduction

Infection with severe acute respiratory syndrome coronavirus 2 (SARS-CoV-2), the causative pathogen of Coronavirus disease 2019 (COVID-19), has resulted in over 6.3 million deaths worldwide [1]. Studies conducted in the first year of the pandemic identified advanced age, select comorbidities, male sex, leukopenia, neutrophilia, and several small nucleotide polymorphisms as major risk factors for disease severity and post-infection mortality [2–4]. Bacterial co-infection in COVID-19 was initially reported to have low prevalence with limited contribution to overall severity and mortality [5–16]. Despite these early reports, recent evidence suggests that bacterial co-infection in COVID-19 may influence mortality, although large-scale multicenter studies have not validated this hypothesis [17].

Reported COVID-19 co-infection rates have ranged from 2 to 8% but consistently appear less than the influenza co-infection rates as reported in the 1918 pandemic and the estimated 34% co-infection rate of the 2009 influenza A (H1N1) pandemic [6, 7, 18–20]. Mortality rates from COVID-19 co-infection have varied widely from twofold compared to non-co-infected COVID-19 patients, to having no effect on mortality among co-infected ICU patients [5, 8, 17, 21]. Several issues confound a reliable determination of co-infection prevalence and associated morbidity, notably inconsistent definitions of co-infection, limited sample size without independent multicenter cohorts, and inclusion of outpatient encounters in co-infection event rate calculations [5, 7]. Here, we defined bacterial co-infection as the presence of a pathogenic isolate from a sterile site, blood, as determined by positive blood cultures taken within 48-h of admission. This approach provides an analysis that discriminates true pathogens from incidental or colonizing organisms, enabling a consistent assessment of co-infection across cohorts and their impact on clinical outcomes.

The primary aims of this retrospective study were to define: (1) the prevalence of COVID-19 co-infections, (2) the impact of COVID-19 co-infection and SARS-CoV-2 variant strain on clinical outcomes including ICU admission, need for invasive mechanical ventilation, and in-hospital mortality utilizing two independent cohorts, and (3) early biomarkers associated with bacterial co-infection. We hypothesized that bacterial co-infection contributes to poor clinical outcomes in COVID-19 subjects irrespective of SARS-CoV-2 variant, and that early recognition of co-infection is possible with routinely gathered laboratory and vital sign measurements.

## Methods

### Study design and population

A multicenter, retrospective cohort study was performed using adult (age: 18–90 years) hospital admissions with a length of stay (LOS) 1–120 days, SARS-CoV-2-positive tests (rapid antigen or polymerase chain reaction) within 48-h of hospital admission, and blood culture evidence of bacterial co-infection in the University of Alabama at Birmingham Health System (UAB) cohort and the Ochsner Louisiana State University Health—Shreveport (OLHS) cohort. The UAB cohort consisted of hospitals from Jefferson, Shelby, and St. Clair counties in Alabama, USA. The OLHS cohort consisted of hospitals across the state of Louisiana, USA. Data extraction was limited to 01/2020–03/2022. Rationale for using blood cultures alone was due to their standardized use, interpretation across both cohorts, and to avoid culture sites where contamination and colonization are prominent. To exclude hospital-acquired infections, we restricted cultures to those collected within 48-h of COVID-19 admission (see Additional file 1: eFig. 1).

COVID-19-positive inpatient encounters were grouped by (1) confirmed bacterial co-infections [positive blood culture(s) taken within 48-h of admission, containing bacterial pathogens; fungal organisms were omitted], (2) clinically suspected co-infections [negative blood culture(s) obtained within 48-h of admission and initiation of treatment with ≥ 2 doses of antimicrobial agents], and (3) no co-infection [no blood cultures collected within 48-h of admission]. Criteria for the confirmed and suspected sub-groupings were modeled after [22] suspected infection international consensus definition and are described in Additional file 1: eFig. 1 [22]. Bacterial organisms recovered from blood culture in the UAB and OLHS cohorts are described in Additional file 1: eFigs. 2–3.

To compare the effect of COVID-19 co-infection on inpatient outcomes to a pre-pandemic cohort, a total of 199,239 COVID-19-negative inpatient encounters from 2010 to 2019 in the UAB health system were assessed for evidence of bacterial infection (Additional file 1: eFig. 8). After exclusion, 99,170 inpatient encounters with adult patients (age: 18–90 years) with a LOS between 1 and 120 days were stratified into confirmed community-acquired bacteremic infection (*n* = 1703), suspected community-acquired bacteremic infection (*n* = 11,795), and no community-acquired bacteremic infection (*n* = 85,672). Detailed information on the pre-COVID-19 pandemic UAB cohort accrual, characteristics, and bacterial pathogens recovered can be found in Additional file 1: eFigs. 8–9, eTable 14.

### Data extraction and primary study outcomes

Primary outcomes studied were in-hospital mortality, ICU admission (anytime), and need for invasive mechanical ventilation. Mechanical ventilation was confirmed by the first date of concomitant recordings of endotracheal tube insertion distance and ventilator settings. Presentation severity was assessed using the physiologic and laboratory measurements required for calculating Systemic Inflammatory Response Syndrome scores (SIRS; range 0 [best] to 4 [worst]) [23]. SIRS variables are tachycardia (heart rate > 90 beats/min), tachypnea (respiratory rate > 20 breaths/min), fever or hypothermia (temperature > 38 or < 36 Celsius [C°]), and leukocytosis, leukopenia, or bandemia (white blood cell count < 4 or > 12 × 10^3^/uL or bandemia ≥ 10%). Rationale for using the SIRS criteria was threefold: 1) SIRS variables are routinely gathered across both cohorts within 24-h of hospital admission, 2) data missingness for all SIRS variables was exceedingly low (3.8% of all included encounters), enabling unbiased outcome modeling without use of any data imputation methods, and 3) unlike the World Health Organization (WHO) 8-point ordinal scale of COVID-19 severity, none of the SIRS variables are inherent to ICU admission or use of mechanical ventilation, which permits unconfounded modeling of these important clinical outcomes [24]. Pre-admission Charlson comorbidity scores were computed using ICD-9/10 diagnosis codes taken prior to each inpatient encounter [25].

### Statistical analysis

Overall cohort statistics were performed using the Wilcoxon rank-sum test, Pearson’s Chi-squared test, or Fisher’s exact test and are reported in Additional file 1: eTable 1. Temporal laboratory measurement trends stratified by COVID-19 bacterial co-infection status and assessed from the day of admission (day 0) to 3 days post-admission. Pooled encounters from the UAB and OLHS cohorts were used for this analysis to increase cohort size for early biomarker trend assessment. Statistical differences between COVID-19 co-infection status groups were assessed using Bonferroni corrected t-tests for multiple comparisons between COVID-19 with confirmed (reference), suspected, and no co-infection subgroups.

Multivariable logistic regression models were used to assess pre- and post-admission risk factors for COVID-19 bacterial co-infection in the UAB and OLHS cohorts independently, using two groups (confirmed bacterial co-infection vs a single group comprised of the suspected and no co-infection groups; Fig. 3). Additional sensitivity testing for pre- and post-admission risk factor models was performed using only the confirmed and suspected co-infection groups (Additional file 1: eTable 15). Univariate and multivariate logistic regression models were used to assess the impact of COVID-19 co-infection on primary clinical outcomes including in-hospital mortality, ICU admission, and invasive mechanical ventilation use. Models were built from the UAB and OLHS cohorts independently unless specified. No assumptions or imputations for missing data were made for any model variables (see Additional file 1: eFig. 5 for variable missingness). Co-linearity of model variables was assessed using Spearman correlation analysis (Additional file 1: eFig. 6). Pre-admission COVID-19 co-infection risk factor model variables included age, sex, and pulmonary, renal, cardiac, and diabetic comorbidities as defined by the Charlson comorbidity index [25]. Post-admission risk factor models used all four components of the SIRS scores and neutrophil-to-lymphocyte ratio computed from laboratory and vitals measurements taken within 24-h of admission. Sensitivity testing for outcome models was performed using the blood culture(-) suspected co-infection cohort as a reference group (with and without data imputation for missing comorbidity and 24-h post-admission SIRS scores) and is reported in Additional file 1: eTables 16–34, eFig. 10.

The effect of COVID-19 co-infection on clinical outcomes was assessed using univariate and multivariable logistic regression with advanced age (≥ 65 years), male sex, pre-admission cardiac, pulmonary, diabetic, and renal comorbidities, and co-infection status model variables. All modeling experiments reported as adjusted odds ratios with bootstrapped (*n* = 1000 iterations) 95% confidence intervals (CI) and the Wald test to determine statistical significance of model variables. All statistical analyses were performed using R (version 4.2, R Foundation).

## Results

### Population characteristics

**Fig. 1:**
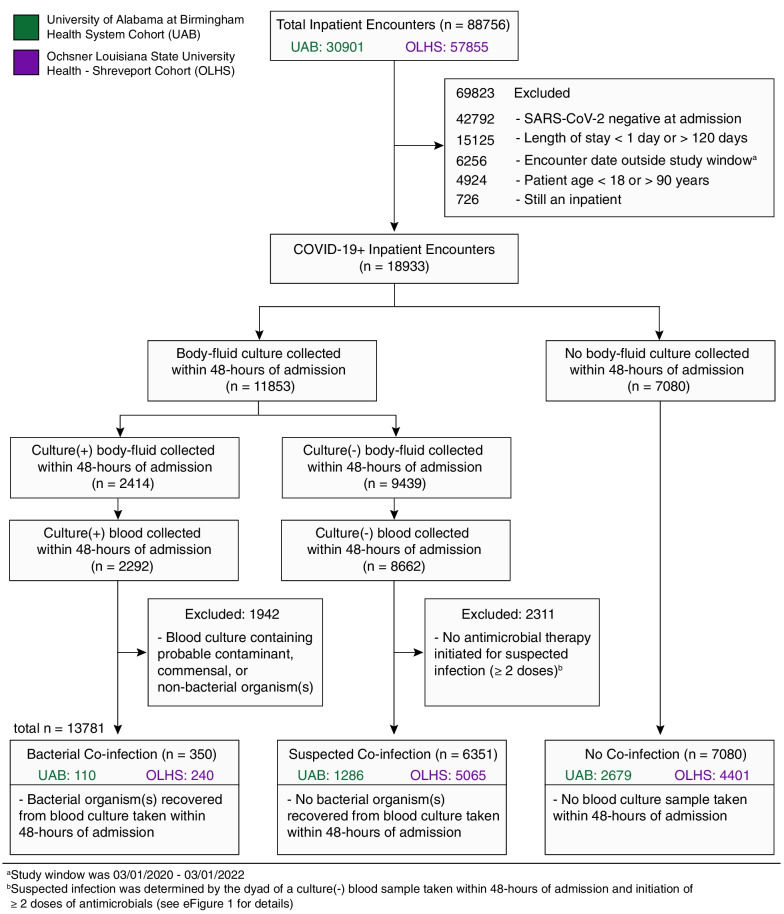
Accrual of COVID-19 bacterial co-infection encounters from the UAB and OLHS cohorts

A total of 88,756 inpatient encounters from hospitals in the UAB (*n* = 30,901) and OLHS (*n* = 57,855) cohorts were assessed in this multicenter retrospective cohort study from 03/2020 to 03/2022. After exclusion, 13,781 inpatient encounters with adult patients (age: 18–90 years), a positive COVID-19 test within 48-h of admission, and a length of stay between 1 and 120 days were further stratified into three groups: confirmed bacterial co-infection, suspected bacterial co-infection, and no bacterial co-infection (Fig. 1). Baseline demographics, outcomes, and therapeutic interventions for patients in the three COVID-19 co-infection groups are listed in Table 1 (see Additional file 1: eTable 1 for overall cohort details). For this analysis, we restricted determination of bacterial co-infection status to blood cultures taken within 48-h of admission to ensure captured cases had high probability of clinically relevant infection. Blood culture-positive co-infection rates were 2.5% (*n* = 110) and 2.7% (*n* = 240) among COVID-19 inpatients in the UAB and OLHS cohorts, respectively. In-hospital mortality for COVID-19 co-infections (UAB: 26%; OLHS: 22%) exceeded that of the suspected (UAB: 24%; OLHS: 12%) and no co-infection groups (UAB: 5.9%; OLHS: 5.1%). This result is in contrast to the 5.9% in-hospital mortality rate observed from inpatient encounters in the pre-COVID-19 pandemic UAB cohort (*n* = 1703) with community-acquired bacteremia (Additional file 1: eTable 14) defined by a positive bacterial blood culture taken within 48-h of admission. *Staphylococcus aureus* was the most common pathogen isolated from blood cultures in all the cohorts (UAB: 29/110 [26%], Additional file 1: eFig. 2; OLHS: 60/250 [24%], Additional file 1: eFig. 3; pre-COVID UAB: 566/1703 [33%], Additional file 1: eFig. 9).

**Table 1:** Characteristics, outcomes, and therapeutics for COVID-19 + inpatient encounters with confirmed, suspected, and no bacterial co-infections in the UAB and OLHS cohorts

COVID-19 + Co-infection status^a^	UAB cohort (2020–2022)			OLHS cohort (2020–2022)		
	Confirmed Blood culture(+), *N* = 110	Suspected Blood culture(−), *N* = 1286	None No blood culture, *N* = 2679	Confirmed Blood culture( +), *N* = 240	Suspected Blood culture(-), *N* = 5065	None No blood culture, *N* = 4401
Age, median (IQR)	65 (51–75)	61 (49–72)	59 (44–70)	66 (53–77)	65 (53–75)	60 (43–72)
*Sex*, *n* (%)						
Female	49 (45)	604 (47)	1417 (53)	124 (52)	2414 (48)	2355 (54)
Male	61 (55)	682 (53)	1262 (47)	116 (48)	2651 (52)	2046 (46)
*Race*, *n* (%)						
White or Caucasian	51 (46)	572 (44)	1304 (49)	136 (57)	2669 (53)	2485 (56)
Black or African American	51 (46)	593 (46)	1131 (42)	96 (40)	2125 (42)	1694 (38)
Asian	5 (4.5)	43 (3.3)	68 (2.5)	1 (0.4)	51 (1.0)	29 (0.7)
Hispanic or Latino	2 (1.8)	40 (3.1)	103 (3.8)	0 (0)	0 (0)	0 (0)
American Indian or Alaska Native	0 (0)	2 (0.2)	6 (0.2)	2 (0.8)	26 (0.5)	24 (0.5)
Pacific Islander or Hawaiian Native	0 (0)	0 (0)	1 (< 0.1)	0 (0)	1 (< 0.1)	1 (< 0.1)
Multiple/other	0 (0)	2 (0.2)	3 (0.1)	2 (0.8)	87 (1.7)	108 (2.5)
Decline/refuse	1 (0.9)	34 (2.6)	63 (2.4)	0 (0)	11 (0.2)	5 (0.1)
Race unknown	0 (0)	0 (0)	0 (0)	3 (1.3)	95 (1.9)	55 (1.2)
Charlson Comorbidity Score, median (IQR)	3 (1–6)	2 (1–4)	1 (0–4)	2 (1–4)	1 (0–3)	1 (0–3)
Unknown	17	385	744	14	494	459
*Inpatient outcomes*						
Inpatient length of stay (days), Median (IQR)	10 (5–20)	9 (5–17)	5 (3–8)	6 (4–11)	6 (3–10)	4 (2–7)
SIRS Score (within 24-h; max = 4), Median (IQR)	2 (1–3)	2 (1–2)	1 (0–2)	3 (2–3)	2 (2–3)	2 (1–2)
Unknown	4	41	56	17	118	299
*In-Hospital Mortality Status*, *n* (%)						
In-hospital deceased	29 (26)	302 (23)	157 (5.9)	52 (22)	632 (12)	223 (5.1)
Discharged living	81 (74)	984 (77)	2522 (94)	188 (78)	4433 (88)	4178 (95)
*Mortality status* (30 days), *n* (%)						
Deceased (30 days)	28 (25)	265 (21)	140 (5.2)	49 (20)	581 (11)	214 (4.9)
Living (30 days)	82 (75)	1021 (79)	2539 (95)	191 (80)	4484 (89)	4187 (95)
*ICU status* (anytime), *n* (%)						
ICU admission	61 (55)	697 (54)	509 (19)	130 (54)	2126 (42)	1214 (28)
No ICU admission	49 (45)	589 (46)	2170 (81)	110 (46)	2939 (58)	3187 (72)
*Mechanical ventilation* (anytime), *n* (%)						
Required ventilation	32 (29)	447 (35)	234 (8.7)	55 (23)	822 (16)	308 (7.0)
No ventilation	78 (71)	839 (65)	2445 (91)	185 (77)	4243 (84)	4093 (93)
*Inpatient therapeutics*						
Antimicrobials^b^ (within 48-h), *n* (%)						
Received antimicrobial	104 (95)	1250 (97)	722 (27)	214 (89)	5013 (99)	1501 (34)
No antimicrobial	6 (5.5)	36 (2.8)	1957 (73)	26 (11)	52 (1.0)	2900 (66)
*Dexamethasone* (within 48-h), *n* (%)						
Received dexamethasone	48 (44)	771 (60)	1311 (49)	90 (38)	2434 (48)	1690 (38)
No dexamethasone	62 (56)	515 (40)	1368 (51)	150 (62)	2631 (52)	2711 (62)
*Pre-admission comorbidities*						
Diabetic, *n* (%)	52 (56)	406 (45)	732 (38)	109 (48)	1716 (38)	1212 (31)
Heart failure or MI, *n* (%)	39 (42)	291 (32)	491 (25)	67 (30)	1069 (23)	874 (22)
Chronic pulmonary disease, *n* (%)	24 (26)	266 (30)	516 (27)	67 (30)	1145 (25)	889 (23)
Renal disease, *n* (%)	40 (43)	302 (34)	432 (22)	80 (35)	1157 (25)	779 (20)
Liver disease, *n* (%)	16 (17)	131 (15)	244 (13)	18 (8.0)	388 (8.5)	310 (7.9)
Vascular disease, *n* (%)	32 (34)	239 (27)	419 (22)	70 (31)	1284 (28)	952 (24)
Cancer (any malignancy), *n* (%)	22 (24)	167 (19)	253 (13)	26 (12)	545 (12)	323 (8.2)
Peptic ulcer disease, *n* (%)	8 (8.6)	45 (5.0)	104 (5.4)	9 (4.0)	121 (2.6)	106 (2.7)
Hemiplegia or paraplegia, *n* (%)	9 (9.7)	44 (4.9)	68 (3.5)	9 (4.0)	106 (2.3)	63 (1.6)
Rheumatoid disease, *n* (%)	8 (8.6)	57 (6.3)	95 (4.9)	7 (3.1)	172 (3.8)	121 (3.1)
Dementia, *n* (%)	13 (14)	70 (7.8)	93 (4.8)	21 (9.3)	250 (5.5)	129 (3.3)
AIDS/HIV, *n* (%)	3 (3.2)	18 (2.0)	31 (1.6)	5 (2.2)	47 (1.0)	25 (0.6)

IQR, interquartile range; ICU, intensive care unit; UAB, University of Alabama at Birmingham Health System; OLHS, Ochsner Louisiana State University Health—Shreveport; SIRS, systemic inflammatory response syndrome score; MI, myocardial infarction

Data represented as number (percentage; %) of patients unless otherwise indicated

^a^COVID-19 + encounters include (1) age range 18–90 years, (2) COVID-19 + test within 48-h of hospital admission, and (3) inpatient length of stay 1–120 days. See methods and Additional file 1: eFig. 1 for complete COVID-19 bacterial co-infection subgroup definition

^b^Suspected co-infection patients in the who did not receive antimicrobials within 48-h (UAB *n* = 36; OLHS *n* = 52) were patients with blood cultures taken between 30 and 48-h post-admission. As a result, their 2 doses of antimicrobial therapy start time fell outside the first 48-h window. All 88 suspected infection patients received at least 2 doses of antimicrobials and were represented by the suspected infection scenario 1 described in eAppendix

### Temporal laboratory trends and biomarkers for COVID-19 bacterial co-infection

**Fig. 2:**
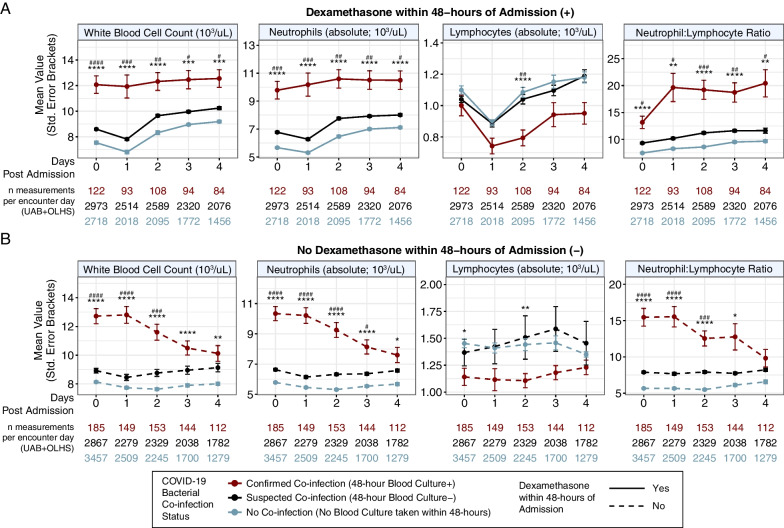
Elevated neutrophil-to-lymphocyte ratio (≥ 15) in COVID-19 bacterial co-infection patients is independent of dexamethasone treatment within 48-h of hospital admission. Post-admission laboratory trends for COVID-19 inpatient encounters pooled from both the UAB and OLHS cohorts and stratified by confirmed, suspected, and no bacterial co-infection and 48-h dexamethasone treatment. Mean laboratory values with standard error bars from day of admission (day 0) to 4 days post-admission are shown for patients with **A** dexamethasone treatment within 48-h (solid lines) and **B** no dexamethasone treatment within 48-h (dashed line), all stratified by COVID-19 co-infection status. Statistical significance was assessed using Bonferroni corrected t-tests: (*p* < 0.0001 = ****/####, *p* < 0.001 = ***/###, *p* < 0.01 = **/##, *p* < 0.05 = */#). Reference group (red): Confirmed co-infection 48-h post-admission blood culture (+). Comparison group 1 (light blue, *): No co-infection. Comparison group 2 (dark blue; #): Suspected co-infection 48-h post-admission blood culture (−). UAB, University of Alabama at Birmingham cohort; OLHS, Ochsner Louisiana State University Health—Shreveport cohort

To identify biomarkers associated with COVID-19 co-infection, post-admission laboratory result trends were assessed. Overall, complete blood count (CBC) with differential measurements including white blood cell count, absolute neutrophil count, absolute lymphocyte count, and neutrophil-to-lymphocyte ratio (NLR) were substantially different between confirmed COVID-19 co-infection versus suspected and no co-infection groups (Fig. 2). We assessed the impact of corticosteroid treatment in COVID-19 management by stratifying the cohort by patients that did or did not receive dexamethasone treatment (within 48-h of admission) and found the NLR remained elevated in the confirmed COVID-19 bacterial co-infection group at each time point post-admission, regardless of dexamethasone treatment (Fig. 2). This finding demonstrates that dexamethasone-induced neutrophilia did not materially influence the observation of high NLR in confirmed bacterial co-infection [26]. Other statistically significant findings for COVID-19 co-infection included elevated lactate and creatinine levels in each post-admission time point, elevated C-reactive protein at the first two time points, and elevated procalcitonin on the day of admission (Additional file 1: eFig. 4).

### COVID-19 bacterial co-infection risk factors

**Fig. 3:**
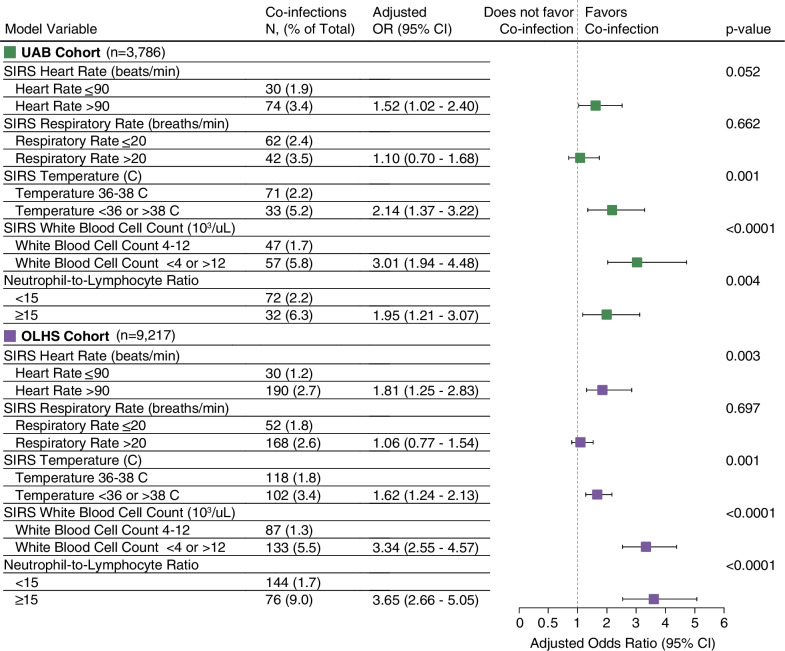
Elevated neutrophil-to-lymphocyte ratio (≥ 15) and select SIRS score components are prognostic indicators of COVID-19 bacterial co-infection. Adjusted odds ratios and 95% CIs for COVID-19 bacterial co-infection post-admission risk factors are shown for the UAB (green) and OLHS (purple) cohorts. Accompanying co-infection rates and Wald test statistical significance are reported for each model variable. SIRS components were restricted to the first reading within 24-h of admission. Neutrophil-to-lymphocyte ratio was computed with first complete blood count measurement taken within 24-h of admission. n, total inpatient encounters; C, Celsius temperature; SIRS, systemic inflammatory response syndrome; CI, Confidence interval; OR, adjusted odds ratio; UAB, University of Alabama at Birmingham cohort; OLHS, Ochsner Louisiana State University Health—Shreveport cohort

Pre-admission and 24-h post-admission risk factors associated with COVID-19 bacterial co-infections were assessed using independent multivariable logistic regression models in the UAB and OLHS cohorts. We observed an increased likelihood of COVID-19 co-infection (Cohort: OR [95%], *p* value) for patients with a pre-admission history of diabetes (UAB: 1.51 [0.95–2.38], *p* = 0.086; OLHS: 1.50 [1.12–1.97], *p* = 0.006) and renal disease (UAB: 1.59 [1.02–2.53], *p* = 0.052; OLHS: 1.53 [1.08–2.14], *p* = 0.01; Additional file 1: eFig. 7). Post-admission risk factors, including three of the four SIRS criterion and a ≥ 15 neutrophil-to-lymphocyte ratio, were associated with increased likelihood of COVID-19 bacterial co-infection across both cohorts. Specifically, we observed statistically significant increased likelihood of co-infection for patients with > 90 heart rate (UAB: 1.52 [1.02–2.40], *p* = 0.052; OLHS: 1.81 [1.25–2.83], *p* = 0.003), temperature < 36C or > 38C (UAB: 2.14 [1.37–3.22], *p* = 0.001; OLHS: 1.62 [1.24–2.13], *p* = 0.001), white blood cell count < 4 or > 12 × 10^3^/uL (UAB: 3.01 [1.94–4.48], *p* < 0.0001; OLHS: 3.34 [2.55–4.57], *p* < 0.0001), and ≥ 15 neutrophil-to-lymphocyte ratio (UAB: 1.95 [1.21–3.07], *p* = 0.004; OLHS: 3.65 [2.66–5.05], *p* < 0.0001) within 24-h of admission (Fig. 3).

### Impact of COVID-19 bacterial co-infection on clinical outcomes

**Fig. 4:**
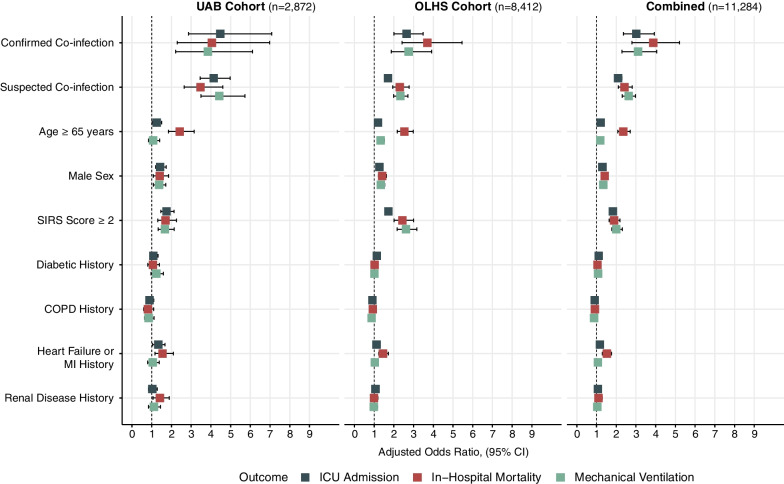
COVID-19 bacterial co-infection confers greater increased risk for in-hospital mortality, mechanical ventilation, and ICU admission than previously identified COVID-19 severity risk factors. Multivariable logistic regression models show confirmed COVID-19 co-infection is the greatest contributor to increased likelihood of in-hospital mortality (red), mechanical ventilation (green), and ICU admission (dark gray). Model variables included culture status within 48-h of admission (reference*: no 48-h blood culture), age ≥ 65 (reference*: age < 65), male sex (reference*: female sex), 24-h post-admission SIRS score ≥ 2 (reference*: 24-h post-admission SIRS score < 2), diabetic history, COPD history, heart failure or MI history, renal disease history (reference*: no pre-admission history of respective comorbidity). For model details see Additional file 1: eTables 2–4. Reference*, reference not shown; SIRS, severe inflammatory response syndrome score; MI, myocardial infarction; COPD, chronic obstructive pulmonary disease; UAB, University of Alabama at Birmingham cohort; OLHS, Ochsner Louisiana State University Health—Shreveport cohort

Multiple risk factors have been associated with COVID-19 severity and mortality including advanced age, male sex, and diabetic, cardiac, renal, and pulmonary comorbidities [4]. Multivariable logistic regression models were used to evaluate the impact of COVID-19 co-infection status on clinical outcomes (Cohort, OR [95% CI], *p* value). After adjusting for advanced age (≥ 65 years), male sex, pre-admission comorbidities (diabetes, COPD, heart failure or myocardial infarction, renal disease), and 24-h post-admission SIRS score ≥ 2, we observed that confirmed co-infection conferred the greatest increased likelihood for in-hospital mortality (UAB: 3.70 [2.42–5.46], *p* < 0.001; OLHS: 4.05 [2.29–6.97], *p* < 0.001), ICU admission (UAB: 4.47 [2.87–7.09], *p* < 0.001; OLHS: 2.65 [2.00–3.48], *p* < 0.001), and need for mechanical ventilation (UAB: 3.84 [2.21–6.12], *p* < 0.001; OLHS: 2.75 [1.87–3.92], *p* < 0.001) (Fig. 4; Additional file 1: eTables 2–4, eTables 5–7, eTables 8–10). To evaluate the role of SARS-CoV-2 variants on bacterial co-infection, multivariable in-hospital mortality modeling was performed using date range-stratified encounters from both UAB and OLHS cohorts (Alpha variant: 03/01/2020–5/31/2021; Delta variant: 06/01/2020–01/01/2022; Omicron variant: 01/02/2022–03/01/2022). Regardless of SARS-CoV-2 variant wave timing, COVID-19 bacterial co-infection conferred the greatest increased likelihood for in-hospital mortality (Alpha: 4.12 [2.71- 6.04], *p* < 0.001; Delta: 3.23 [1.65–5.74], *p* < 0.001; Omicron: 5.49 [1.98–13.8], *p* < 0.001; Additional file 1: eTables 11–13).

## Discussion

In this multicenter retrospective study of 13,781 COVID-19 inpatient encounters, our results demonstrate an increased risk of ICU admission, mechanical ventilation, and in-hospital mortality conferred by COVID-19 bacterial co-infection that substantially exceeds previously described risk factors for severity and mortality (e.g., advanced age, male sex, select comorbidities) [4]. The external validity of this result is enhanced by use of data spanning the two years of the pandemic (2020–2022) across two independent cohorts, as well as a 10-year pre-COVID-19 pandemic comparator cohort (2010–2019). Our investigation identified laboratory trends associated with COVID-19 bacterial co-infection and provide evidence that ≥ 15 NLR, temperature, white blood cell count, and heart rate components of the SIRS criterion can help healthcare providers discriminate COVID-19 bacterial co-infections within 24-h of admission. These results emphasize the role of bacteria in SARS-CoV-2 mortality and highlight the potential for NLR as a rapid and easily available prognostic biomarker of bacterial co-infection, and relatedly, disease severity.

A strength of this study is the use of large, demographically diverse, independent cohorts. The UAB cohort (*n* = 4075) reflects an academic hospital and level I trauma center servicing five surrounding states. The OLHS cohort (*n* = 9706) includes encounters from rural, suburban, and academic medical centers across the state of Louisiana. Despite the different clinical settings, both cohorts overall were well matched for patient age, race, sex, and inpatient LOS. Due to sufficiently sized cohorts, all risk factor and outcome modeling performed in this study did not use any form of imputed data. In agreement with previous studies, we found COVID-19 bacterial co-infection to be relatively infrequent in both UAB (2.5%, *n* = 110) and OLHS cohorts (2.7%, *n* = 240), with *Staphylococcus aureus* and *Escherichia coli* as the most frequent Gram-positive and Gram-negative pathogens recovered from 48-h post-admission blood cultures, respectively [5].

Prior studies have explored early biomarkers of COVID-19 co-infections including a 2021 multi-cohort study that reported an elevated baseline white blood cell count and stepwise decrease in CRP at two time points (admission and 48–72-h later) were sufficient to exclude COVID-19 bacterial co-infection in 46% of cases [27]. A crucial limitation to this approach is the requirement of 3 laboratory measurements over a 72-h period, in addition to the modest observed exclusion rate. Here, our results confirm both the elevated CRP and white blood cell count findings as indicators of bacterial co-infection in COVID-19. In addition, we found elevated NLR, lactate, creatinine, and procalcitonin at 24-h post-admission, which raises the possibility of a novel co-infection prediction score that could help overcome the clinical lag associated with culture data and sequential (48–72-h later) laboratory values. Further studies will be needed to inform the robustness of elevated NLR for co-infection detection both in the context of COVID-19 and other viral co-infections such as influenza.

Bacterial co-infection is a major source of morbidity and mortality in the context of respiratory viral infections. A recent retrospective study from Lui et al. reported a 6.8% bacterial co-infection rate with influenza A or B viruses, parainfluenza virus, or respiratory syncytial virus with 10–13% 30-day mortality rate [28]. Our results show that COVID-19 patients with confirmed bacteremic co-infections have double the 30-day mortality rate (UAB: 25%, OLHS: 20%) when compared to influenza virus bacterial co-infections [28]. We determined that COVID-19 bacterial co-infections had a profound impact on increased likelihood of in-hospital mortality, ICU admission, and need for mechanical ventilation. Across both cohorts, the odds ratio for in-hospital mortality for COVID-19 co-infection was higher than the reported mortality odds ratio for influenza virus bacterial co-infection and was independent of SARS-CoV-2 variant [17, 28, 29]. Importantly, the 26% and 22% in-hospital mortality rates observed in the UAB and OLHS cohorts were fivefold higher than the community-acquired bacteremia encounters from the UAB pre-COVID-19 pandemic comparator cohort (5.9%) These results strongly suggest an underappreciated interaction between bacterial pathogens and SARS-CoV-2, and their impact on clinical outcomes.

In addition to the blood culture-positive co-infection group, the suspected co-infection population displayed increased odds ratios for markers of severe disease. This is partially explained by the association of increased disease severity at presentation prompting clinicians to manage the possibility of bacterial co-infection with initiation of antibacterial therapy and collection of blood cultures. However, another consideration is that co-infection for this study was strictly defined to pathogens recovered from blood culture. From a physiologic perspective, co-infection includes a broad variety of organisms involving multiple different tissues. However, accurately differentiating relevant pathogens from recovery of incidental or colonizing organisms is inherently difficult, particularly from sites such as the respiratory tract. Further, recovery of pathogens from the blood is influenced by the presence of antimicrobials prior to culture collection, either as an outpatient or shortly after presentation. Despite the restrictiveness of our approach and the low frequency of bacterial co-infection, our observations attribute a massive effect on morbidity and mortality to co-infection when viewed in context of the estimated 4 million hospitalizations for COVID-19 in the United States [30].

A critical question remains regarding the optimal therapeutic management of high-risk presentations of COVID-19 and potentially other respiratory viral pathogens. Although targeted antimicrobial therapy remains a mainstay of modern management of critically ill patients, results from a 2022 multi-omic study comparing broncho-alveolar lavage samples from influenza virus and COVID-19 co-infections showed that initiation of antimicrobials during COVID-19 co-infection did not alter lung inflammation [31]. Notably, here nearly all patients identified in the co-infected group, and all patients in the suspected co-infection groups, had prompt initiation of antimicrobial treatment. These results suggest that antimicrobials alone may be insufficient to prevent progression to severe disease and worse clinical outcomes in COVID-19 bacterial co-infections. Interestingly, a variety of immune modulators for moderate and severe COVID-19 have been found to improve clinical outcomes, with limited evidence for secondary infections [24, 32–35]. Collectively, these observations suggest the possibility that targeted immune suppression may be beneficial in managing severe COVID-19 even in the setting of co-infection. Future multicenter studies focused on determining immunological correlates for disease severity and bacterial co-infection are warranted for COVID-19 and other respiratory viral pathogens.

This study has limitations. By applying a strict definition of bacterial co-infection based on blood cultures taken within 48-h of admission, our study deliberately decreased sensitivity for bacterial co-infection overall, and excluded other types of pathogens. We eliminated culture results from bacterial species likely to represent contaminant or colonizer species unlikely to represent active infection (Additional file 1: eFigs. 2–3). Another limitation in our analyses was poor coverage of vaccination status at time of presentation from both cohorts. This prevented inclusion of vaccination as a model variable for all outcome models, although our analysis that SARS-CoV-2 variant did not impact our findings, suggests that vaccination status did not drive our observations.

In conclusion, this retrospective multicenter analysis identified COVID-19 bacterial co-infections in independent cohorts using 48-h blood culture results. Our results show elevated neutrophil-to-lymphocyte ratio and components of the SIRS criteria as early, unambiguous, biomarkers of COVID-19 bacterial co-infections. Finally, we assessed the clinical impact of COVID-19 co-infection and found increased likelihood of ICU admission, mechanical ventilation, and in-hospital mortality compared to COVID-19 infection alone.

## Conclusions

This retrospective multicenter analysis identified COVID-19 bacterial co-infections in independent cohorts using 48-h blood culture results. Our analysis confirmed elevated ≥ 15 NLR and components of the SIRS criteria were, early, unambiguous, biomarkers of COVID-19 bacterial co-infections. We assessed the clinical impact of COVID-19 co-infection and found an increased likelihood of ICU admission, mechanical ventilation, and in-hospital mortality compared to COVID-19 infection alone that was independent of SARS-CoV-2 variant (e.g., alpha, delta, omicron). Finally, we show that COVID-19 bacteremic co-infection has a five-fold higher in-hospital mortality rate compared to pre-COVID-19 pandemic inpatients with bacteremia within 48-h of admission.

## Supplementary Information

**Additional file 1**. Supplement Text.

## Data Availability

Select de-identified electronic medical record data from the University of Alabama at Birmingham (UAB) 2020–2022 cohort, the Ochsner Louisiana Health System—Shreveport (OLHS) 2020–2022 cohort, and the UAB pre-COVID-19 pandemic 2010–2019 cohort can be made available upon request and signing of institutional data use agreements.

## References

[1] World Health Organization. Weekly epidemiological update on COVID-19 - 3 August 2022. Emergency Situational Updates, 2022; 103. http://www.who.int/emergencies/diseases/novel-coronavirus-2019/situation-reports

[2] Zhou F, Yu T, Du R (2020). Clinical course and risk factors for mortality of adult inpatients with COVID-19 in Wuhan, China: a retrospective cohort study. Lancet.

[3] Zeberg H, Pääbo S (2020). The major genetic risk factor for severe COVID-19 is inherited from Neanderthals. Nature.

[4] Dessie ZG, Zewotir T (2021). Mortality-related risk factors of COVID-19: a systematic review and metaanalysis of 42 studies and 423,117 patients. BMC Infect Dis.

[5] Russell CD, Fairfield CJ, Drake TM (2021). Co-infections, secondary infections, and antimicrobial use in patients hospitalised with COVID-19 during the first pandemic wave from the ISARIC WHO CCP-UK study: a multicentre, prospective cohort study. Lancet Microbe.

[6] Moreno-García E, Puerta-Alcalde P, Letona L (2022). Bacterial co-infection at hospital admission in patients with COVID-19. Int J Infect Dis.

[7] Langford BJ, So M, Raybardhan S (2020). Bacterial co-infection and secondary infection in patients with COVID-19: a living rapid review and meta-analysis. Clin Microbiol Infect.

[8] Alqahtani A, Alamer E, Mir M (2022). Bacterial Coinfections Increase Mortality of Severely Ill COVID-19 Patients in Saudi Arabia. Int J Environ Res Public Health.

[9] Mirzaei R, Goodarzi P, Asadi M (2020). Bacterial co-infections with SARS-CoV-2. IUBMB Life.

[10] Rawson TM, Moore LSP, Zhu N (2020). Bacterial and fungal coinfection in individuals with coronavirus: a rapid review to support COVID-19 antimicrobial prescribing. Clin Infect Dis.

[11] Zhu X, Ge Y, Wu T (2020). Co-infection with respiratory pathogens among COVID-2019 cases. Virus Res.

[12] Hughes S, Troise O, Donaldson H, Mughal N, Moore LSP (2020). Bacterial and fungal coinfection among hospitalized patients with COVID-19: a retrospective cohort study in a UK secondary-care setting. Clin Microbiol Infect.

[13] Garcia-Vidal C, Sanjuan G, Moreno-García E (2021). Incidence of co-infections and superinfections in hospitalized patients with COVID-19: a retrospective cohort study. Clin Microbiol Infect.

[14] Westblade LF, Simon MS, Satlin MJ (2021). Bacterial coinfections in coronavirus disease 2019. Trends Microbiol.

[15] Fattorini L, Creti R, Palma C (2020). Bacterial coinfections in COVID-19: an underestimated adversary. Annali dell’Istituto superiore di sanita.

[16] Kim D, Quinn J, Pinsky B, Shah NH, Brown I (2020). Rates of co-infection between SARS-CoV-2 and other respiratory pathogens. J Am Med Assoc.

[17] Hedberg P, Johansson N, Ternhag A, Abdel-Halim L, Hedlund J, Nauclér P (2022). Bacterial co-infections in community-acquired pneumonia caused by SARS-CoV-2, influenza virus and respiratory syncytial virus. BMC Infect Dis.

[18] Lansbury L, Lim B, Baskaran V, Lim WS (2020). Co-infections in people with COVID-19: a systematic review and meta-analysis. J Infect.

[19] Lim WS, Baudouin SV, George RC, et al. BTS guidelines for the management of community acquired pneumonia in adults: update 2009. Thorax 2009;64 Suppl 3:iii1–55. 10.1136/thx.2009.121434 19783532

[20] Chertow DS, Memoli MJ (2013). Bacterial coinfection in influenza: a grand rounds review. J Am Med Assoc.

[21] Petty LA, Flanders SA, Vaughn VM, Ratz D, O’Malley M, Malani AN., Washer L, Kim T, Kocher KE, Kaatz S, Czilok T, McLaughlin E, Prescott HC, Chopra V, Gandhi T (2022). Risk factors and outcomes associated with community-onset and hospital-acquired coinfection in patients hospitalized for coronavirus disease 2019 (COVID-19): A multihospital cohort study. Infect Control Hosp Epidemiol.

[22] Seymour CW, Liu VX, Iwashyna TJ (2016). Assessment of clinical criteria for sepsis: for the third international consensus definitions for sepsis and septic shock (Sepsis-3). J Am Med Assoc.

[23] Bone RC, Balk RA, Cerra FB (1992). Definitions for sepsis and organ failure and guidelines for the use of innovative therapies in sepsis. Chest.

[24] Beigel JH, Tomashek KM, Dodd LE (2020). Remdesivir for the treatment of covid-19—final report. N Engl J Med.

[25] Charlson ME, Pompei P, Ales KL, MacKenzie CR (1987). A new method of classifying prognostic comorbidity in longitudinal studies: development and validation. J Chronic Dis.

[26] Mishler JM, Emerson PM (1977). Development of Neutrophilia by serially increasing doses of dexamethasone. Br J Haematol.

[27] Mason CY, Kanitkar T, Richardson CJ (2021). Exclusion of bacterial co-infection in COVID-19 using baseline inflammatory markers and their response to antibiotics. J Antimicrob Chemother.

[28] Liu Y, Ling L, Wong SH (2021). Outcomes of respiratory viral-bacterial co-infection in adult hospitalized patients. EClinicalMedicine.

[29] Shafran N, Shafran I, Ben-Zvi H (2021). Secondary bacterial infection in COVID-19 patients is a stronger predictor for death compared to influenza patients. Sci Rep.

[30] Centers for Disease Control and Prevention. COVID Data Tracker. Atlanta, GA: US Department of Health and Human Services, CDC; 2022, October 12. https://covid.cdc.gov/covid-data-tracker

[31] Cambier S, Metzemaekers M, Carvalho AC de, et al. Atypical response to bacterial coinfection and persistent neutrophilic bronchoalveolar inflammation distinguish critical COVID-19 from influenza. JCI Insight 2022. 10.1172/jci.insight.155055 PMC8765057 34793331

[32] Horby P, Lim WS, Group RC (2021). Dexamethasone in hospitalized patients with covid-19. N Engl J Med.

[33] Kalil AC, Patterson TF, Mehta AK (2021). Baricitinib plus remdesivir for hospitalized adults with covid-19. N Engl J Med.

[34] Wolfe CR, Tomashek KM, Patterson TF, et al. Baricitinib versus dexamethasone for adults hospitalised with COVID-19 (ACTT-4): a randomised, double-blind, double placebo-controlled trial. Lancet. Respiratory medicine 2022. 10.1016/S2213-2600(22)00088-1 PMC9126560 35617986

[35] Group RC (2021). Tocilizumab in patients admitted to hospital with COVID-19 (RECOVERY): a randomised, controlled, open-label, platform trial. Lancet.

