# Supplementary material for: COVID-19 bacteremic co-infection is a major risk factor for mortality, ICU admission, and mechanical ventilation
Source: Crit Care. 2023 Jan 23;27:34. doi: 10.1186/s13054-023-04312-0 (PMC9868503; doi:10.1186/s13054-023-04312-0)
Supplement: Supplementary file 1 — Additional file 1. Supplement Text. [file 13054_2023_4312_MOESM1_ESM.docx]

**SUPPLEMENTAL MATERIALS**

**eAppendix**

**Supplementary Methods:** Discussion of phenotyping community acquired infection and co-infection in the UAB and OLHS cohorts (2020-2022) and the UAB pre-COVID-19 pandemic cohort (2010-2019)

**eFigures**

eFigure 1: COVID-19 bacterial co-infection cohort design and accompanying microbial organism exclusion list 4

eFigure 2: Bacteria isolated from COVID-19 blood cultures taken within 48-hours of admission in the UAB Cohort 5

eFigure 3: Bacteria isolated from COVID-19 blood cultures taken within 48-hours of admission in the OLHS Cohort 6

eFigure 4: Neutrophil-to-lymphocyte ratio is elevated in COVID-19 bacterial co-infection patients at all time points post-admission. 7

eFigure 5: Distribution of missing candidate variables from the UAB and OLHS cohorts. 8

eFigure 6: Spearman correlation of candidate variables (continuous) from the UAB and OLHS cohorts. 9

eFigure 7: Pre-admission Risk Factors for COVID-19 Bacterial Co-infection for UAB and OLHS Cohorts. 10

eFigure 8: Accrual of pre-COVID-19 pandemic community acquired bacterial infection encounters from the UAB cohort (2010-2019) 11

eFigure 9: Bacterial isolated from inpatient blood cultures taken within 48-hours of admission in the UAB pre-COVID-19 pandemic cohort (2010-2019) 12

eFigure 10: In-Hospital Mortality, Mechanical Ventilation, and ICU Admission Multi-variate Logistic Regression Outcome Models (Sensitivity Testing with Blood Culture(-) Suspected Co-infection as Reference) 41

**eTables**

eTable 1: Overall Comparison of UAB and OLHS Cohort Characteristics 13

eTable 2: OLHS Cohort In-Hospital Mortality Models 15

eTable 3: UAB Cohort In-Hospital Mortality Models 16

eTable 4: Combined UAB and OLHS Cohorts In-Hospital Mortality Models 17

eTable 5: OLHS Cohort Mechanical Ventilation Models 18

eTable 6: UAB Cohort Mechanical Ventilation Models 19

eTable 7: Combined UAB and OLHS Cohorts Mechanical Ventilation Models 20

eTable 8: OLHS Cohort ICU Admission Models 21

eTable 9: UAB Cohort ICU Admission Models 22

eTable 10: Combined UAB and OLHS Cohorts ICU Admission Models 23

eTable 11: Combined UAB and OLHS Cohorts In-Hospital Mortality Models during COVID-19 Alpha-Variant Wave (03/01/2020-05/31/2021) 24

eTable 12: Combined UAB and OLHS Cohorts In-Hospital Mortality Models during COVID-19 Delta-Variant Wave (06/01/2021-01/01/2022) 25

eTable 13: Combined UAB and OLHS Cohorts In-Hospital Mortality Models during COVID-19 Omicron-Variant Wave (01/02/2022- 03/02/2022) 26

eTable 14: Characteristics, outcomes, and therapeutics for inpatient encounters with confirmed, suspected, and no bacterial infections in the UAB pre-COVID-19 pandemic cohort (2010-2019) 27

eTable 15: 24-Hour Post-Admission Risk Factors of COVID-19 Bacterial Co-infection (Sensitivity Testing with Blood Culture(-) Suspected Co-infection and Blood Culture(+) Confirmed Co-infection groups only) 31

eTable 16: UAB Cohort In-Hospital Mortality Models (Sensitivity Testing with Blood Culture(-) Suspected Co-infection as Reference) 32

eTable 17: OLHS Cohort In-Hospital Mortality Models (Sensitivity Testing with Blood Culture(-) Suspected Co-infection as Reference) 33

eTable 18: Combined UAB and OLHS Cohorts In-Hospital Mortality Models (Sensitivity Testing with Blood Culture(-) Suspected Co-infection as Reference) 34

eTable 19: UAB Cohort Mechanical Ventilation Models (Sensitivity Testing with Blood Culture(-) Suspected Co-infection as Reference) 35

eTable 20: OLHS Cohort Mechanical Ventilation Models (Sensitivity Testing with Blood Culture(-) Suspected Co-infection as Reference) 36

eTable 21: Combined UAB and OLHS Cohorts Mechanical Ventilation Models (Sensitivity Testing with Blood Culture(-) Suspected Co-infection as Reference) 37

eTable 22: UAB Cohort ICU Admission Models (Sensitivity Testing with Blood Culture(-) Suspected Co-infection as Reference) 38

eTable 23: OLHS Cohort ICU Admission Models (Sensitivity Testing with Blood Culture(-) Suspected Co-infection as Reference) 39

eTable 24: Combined UAB and OLHS Cohorts ICU Admission Models (Sensitivity Testing with Blood Culture(-) Suspected Co-infection as Reference) 40

eTable 25: OLHS Cohort In-Hospital Mortality Models (Sensitivity Testing with Imputed Data) 43

eTable 26: UAB Cohort In-Hospital Mortality Models (Sensitivity Testing with Imputed Data) 44

eTable 27: Combined UAB and OLHS Cohorts In-Hospital Mortality Models (Sensitivity Testing with Imputed Data) 45

eTable 28: OLHS Cohort In-Hospital Mortality Models (Sensitivity Testing with Blood Culture(-) Suspected Co-infection as Reference and Imputed Data) 47

eTable 29: UAB Cohort In-Hospital Mortality Models (Sensitivity Testing with Blood Culture(-) Suspected Co-infection as Reference and Imputed Data) 48

eTable 30: Combined UAB and OLHS Cohorts In-Hospital Mortality Models (Sensitivity Testing with Blood Culture(-) Suspected Co-infection as Reference and Imputed Data) 49

eTable 31: Combined UAB and OLHS Cohorts In-Hospital Mortality Models (Sensitivity Testing with Hematologic Disease History) 51

eTable 32: Combined UAB and OLHS Cohorts In-Hospital Mortality Models (Sensitivity Testing with Hematologic Disease History and Blood Culture(-) Suspected Co-infection as Reference) 52

eTable 33: UAB Cohort In-Hospital Mortality Models (Sensitivity Testing with Solid Organ Transplant Status) 53

eTable 34:UAB Cohort In-Hospital Mortality Models (Sensitivity Testing with Solid Organ Transplant Status and Blood Culture(-) Suspected Co-infection as Reference) 54

**Sensitivity Testing**

**Section 1:** Reference group testing with univariate and multivariate logistic regression models.

**Section 2**: Data imputation testing with univariate and multivariate logistic regression models.

**Section 3**: Combined reference group and data imputation testing with univariate and multivariate logistic regression models.

**Section 4:** Additional pre-existing condition/comorbidity history univariate and multivariate logistic regression models.

**eAppendix.** Supplemental Methods

1. Community acquired bacteremic co-infection in the UAB and OLHS COVID-19 cohorts (2020-2022)
   - Community acquired co-infection was defined as a blood culture taken within 48-hours of hospitalization that grew a bacterial organism considered to be pathogenic
     - To prevent capture of contaminant or pseudo-bacteremia in our confirmed co-infection cohort, we excluded blood culture results taken within 48-hours of admission with any of the following results:
       - Contaminant: Free text indicating a probable contaminant or error in the culture lab
         - Example(s): “probable skin contaminant” or “coagulase negative staphylococcus”
       - Non-Specific: unclear or gram level descriptions of blood culture growth
         - Example: “gram positive cocci present”
       - Commensals: non-pathogenic organisms without the presence of a known pathogenic species (see eFigure1 for list of excluded commensal species)
         - Example: “staphylococcus epidermidis”
       - Non-bacterial: fungal, viral, or parasitic organisms
         - Example: “mold or fungal growth”
   - Suspected community acquired co-infection was defined by the dyad of either:
     - 1) a blood culture taken with 48-hours of admission followed by antimicrobial therapy within 96-hours (≥2 total doses)
     - 2) antimicrobial therapy (≥2 total doses) followed by blood culture taken within 24-hours of the first antimicrobial start time (note: blood cultures outside the 48-hour post admission window were not considered)
   - No community acquired co-infection was defined as an inpatient admission without any evidence of a body-fluid culture taken within 48-hours of admission
2. The UAB pre-COVID-19 pandemic cohort (2010-2019) was assessed using the same criteria as the UAB and OLHS cohorts (2020-2022) with the caveat of bacterial infection as defined by positive blood culture taken within 48-hours of admission, rather than co-infection, due to the lack of a COVID-19+ test (see eFigure 8-9, eTable 14).

**eFigure 1: COVID-19 bacterial co-infection cohort design and accompanying microbial organism exclusion list**

**eFigure 2: Bacteria isolated from COVID-19 blood cultures taken within 48-hours of admission in the UAB Cohort**

**eFigure 3: Bacteria isolated from COVID-19 blood cultures taken within 48-hours of admission in the OLHS Cohort**


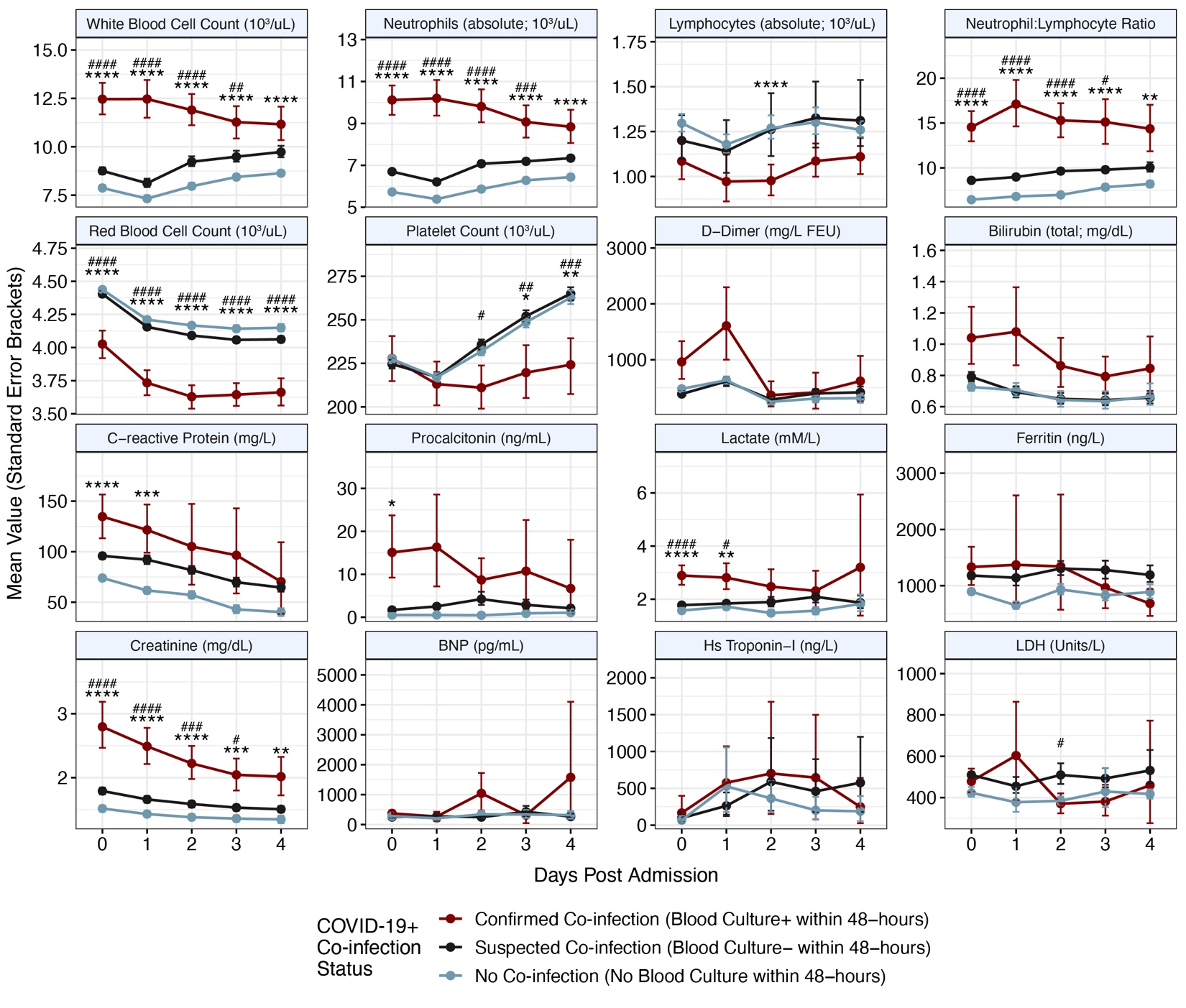


**eFigure 4: Neutrophil-to-lymphocyte ratio is elevated in COVID-19 bacterial co-infection patients at all time points post-admission.**

Post-admission laboratory trends for COVID-19 inpatients from both the UAB and OLHS cohorts, stratified by confirmed, suspected, and no suspected co-infection. Mean laboratory values with standard error brackets from day of admission (day 0) to 4 days post-admission are shown with stratification for COVID-19 co-infection status. Statistical significance was assessed using Bonferroni corrected t-tests: (p<0.0001=****/####, p<0.001=***/###, p<0.01=**/##, p<0.05=*/#). Reference group (red): Confirmed co-infection 48-hour post-admission blood culture (+). Comparison group 1 (light blue, *): No co-infection. Comparison group 2 (dark blue; #): Suspected co-infection 48-hour post-admission blood culture (-).


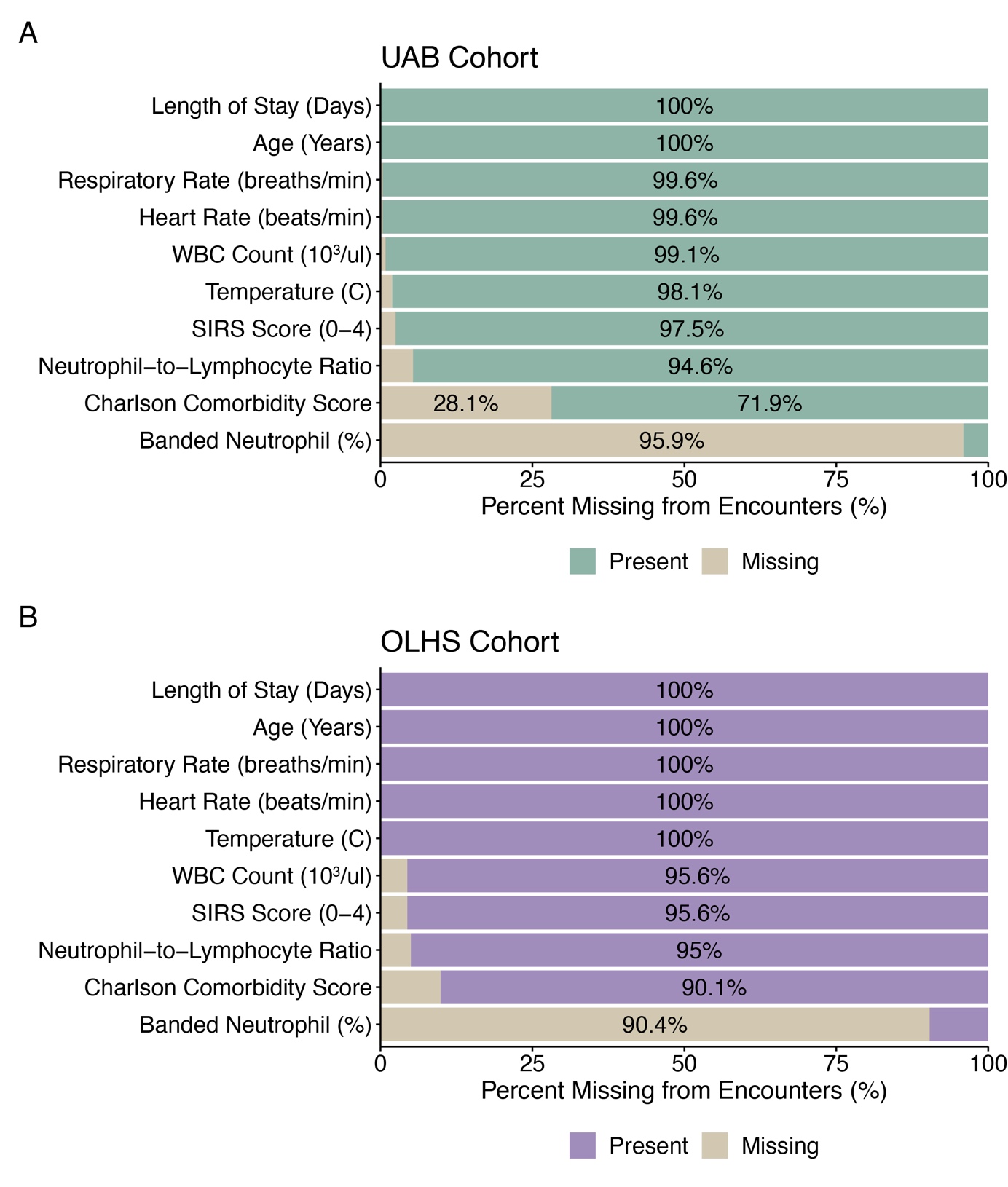


**eFigure 5: Distribution of missing candidate variables from the UAB and OLHS cohorts.**

*(*A-B) All sub-figures represent SARSCoV-2 positive encounters stratified co-infection status with number of distinct encounters displayed above each plot (see eFigure 1 for co-infection definitions). Gold bars represent the percentage of data that is missing in both plots (green for present data in the UAB cohort and purple for present data in the OLHS cohort). Percentage value text below 10% have been omitted for readability. SIRS scores *and n*eutrophil-to-lymphocyte ratios were computed from laboratory values *measured* within 24-hours of admission. If two different values had the same timestamp, either the maximum or minimum value was taken in accord with the following assignments: [Maximum lab values: SIRS neutrophil bands, SIRS heart rate, SIRS respiratory rate, SIRS white blood cell count, SIRS temperature, absolute lymphocyte count, absolute neutrophil count]. Abbreviations: SIRS, System Inflammatory Response Syndrome; WBC, White Blood Cell Count; UAB, University of Alabama at Birmingham cohort; OLHS, Ochsner Louisiana State University Health – Shreveport cohort.


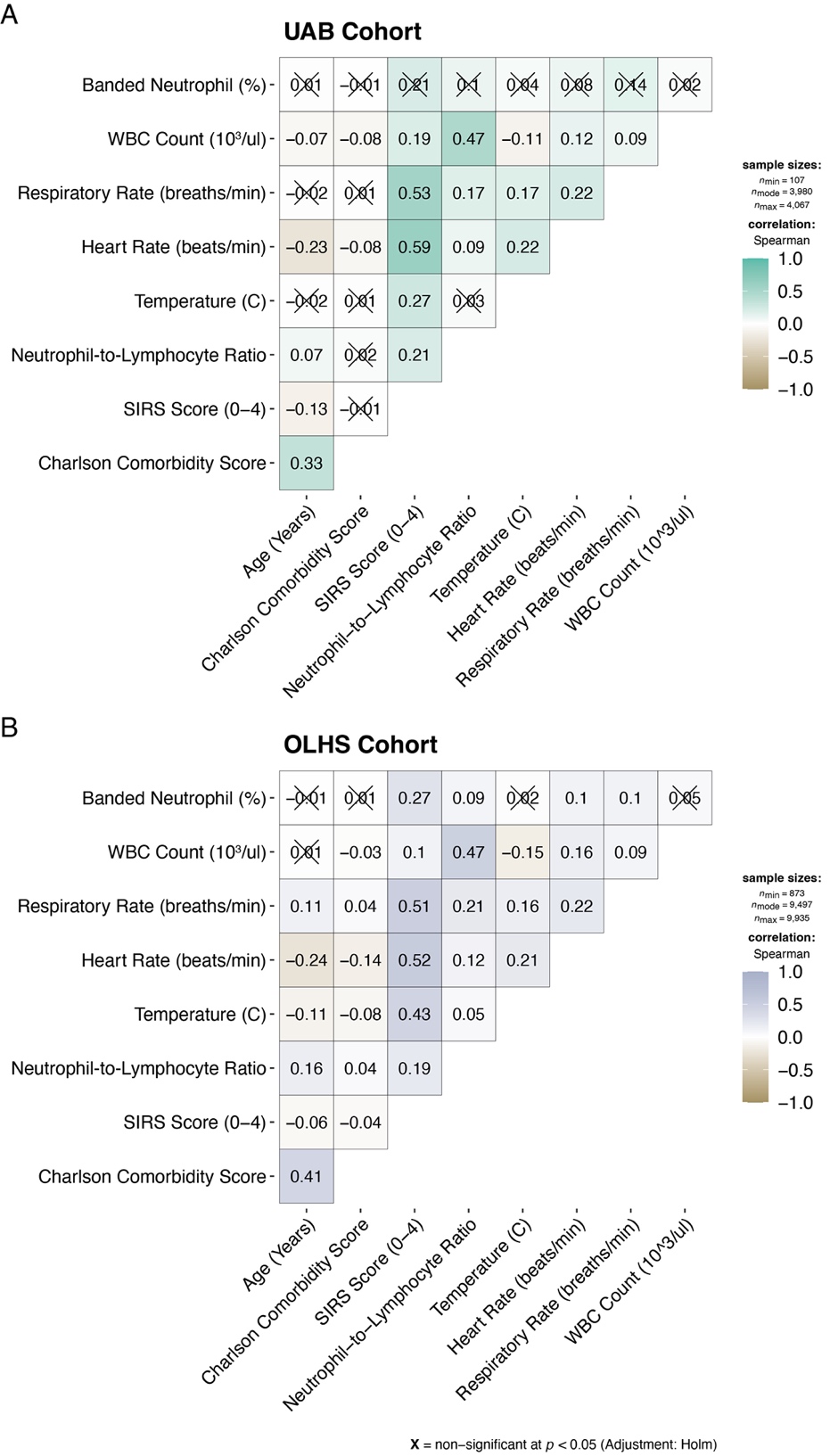


**eFigure 6: Spearman correlation of candidate variables (continuous) from the UAB and OLHS cohorts.**

(A-B) Positive and negative correlations are represented with green and gold color (UAB) or purple and gold color (OLHS), respectively. Non-significant Holm adjusted p-values between two variables are denoted with a black cross over the correlation coefficient. Abbreviations: WBC, White Blood Cell Count; SIRS, Systemic Inflammatory Response Syndrome; UAB, University of Alabama at Birmingham cohort; OLHS, Ochsner Louisiana State University Health – Shreveport cohort.


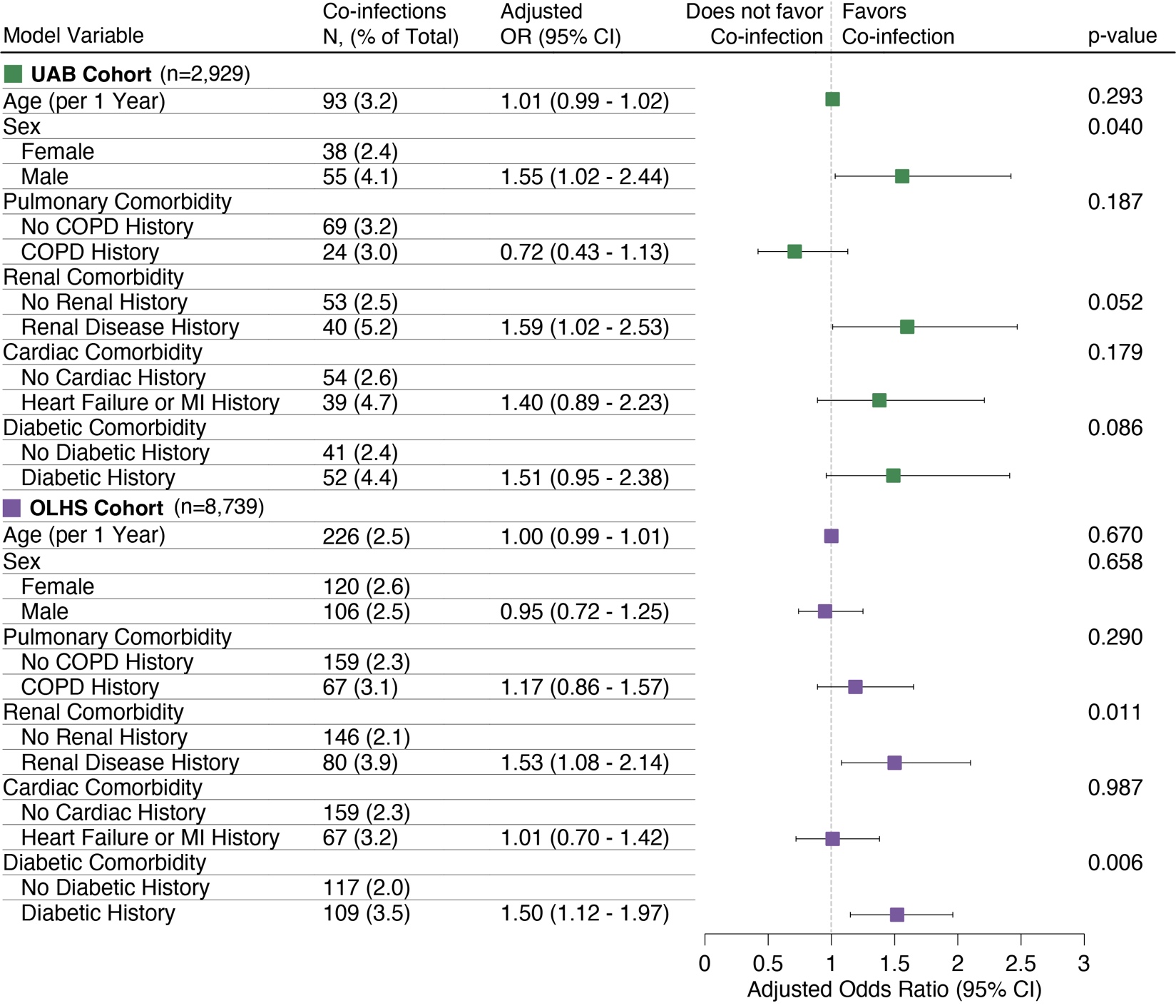


**eFigure 7: Pre-admission Risk Factors for COVID-19 Bacterial Co-infection for UAB and OLHS Cohorts.**

Shown are adjusted odds ratios and 95% CIs for pre-admission risk factors associated with COVID-19 co-infection from the UAB cohort (green) and the OLHS cohort (purple). Accompanying co-infection rates and Wald test statistical significance are reported for each model variable. Inpatient encounters without pre-existing condition or diagnosis codes or any missing demographic data were omitted from this analysis. COPD, Chronic Obstructive Pulmonary Disease; MI, Myocardial Infarction; CI, Confidence interval; OR, adjusted odds ratio; UAB, University of Alabama at Birmingham cohort; OLHS, Ochsner Louisiana State University Health – Shreveport cohort.


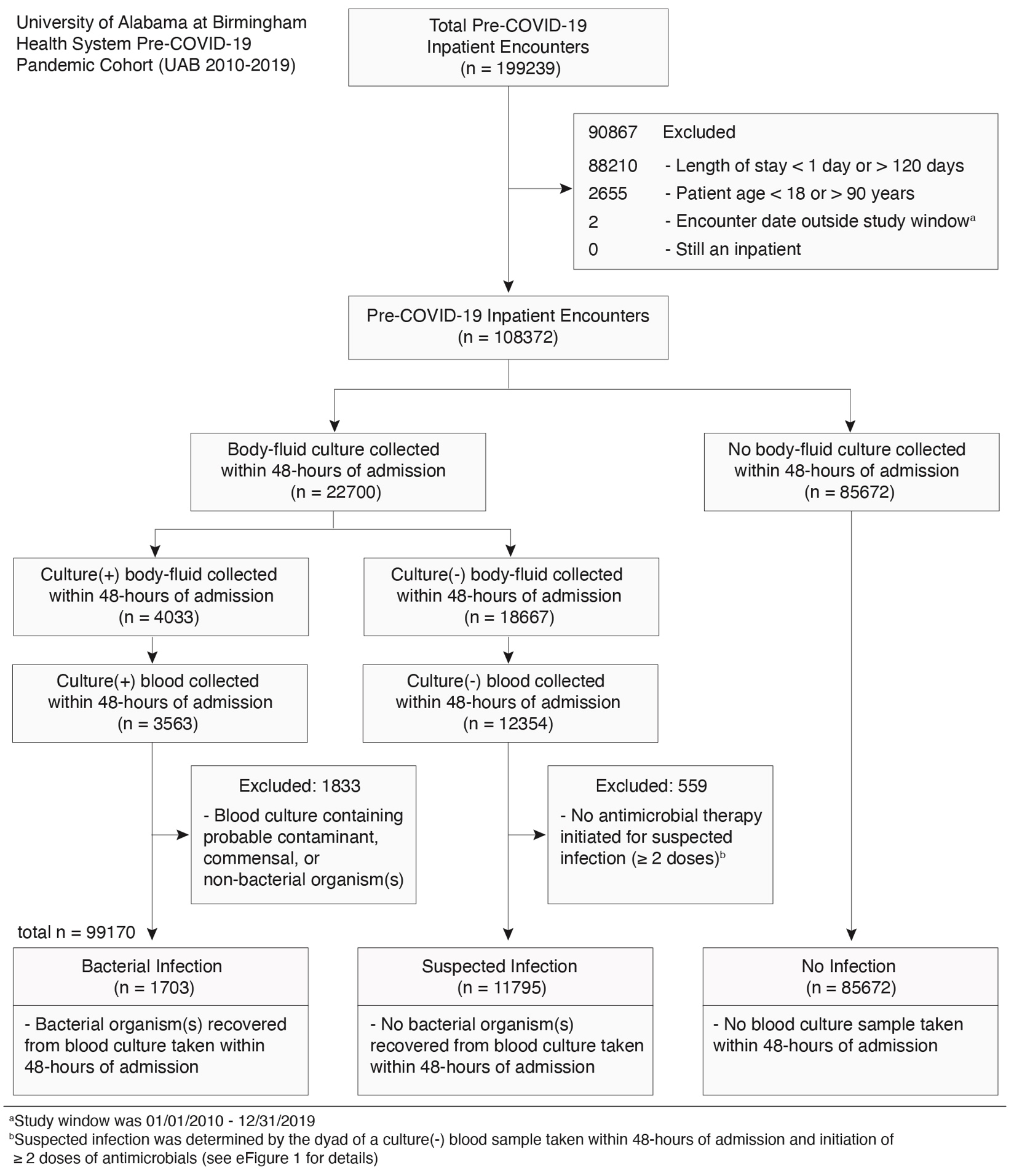


**eFigure 8: Accrual of pre-COVID-19 pandemic community acquired bacterial infection encounters from the UAB cohort (2010-2019)**


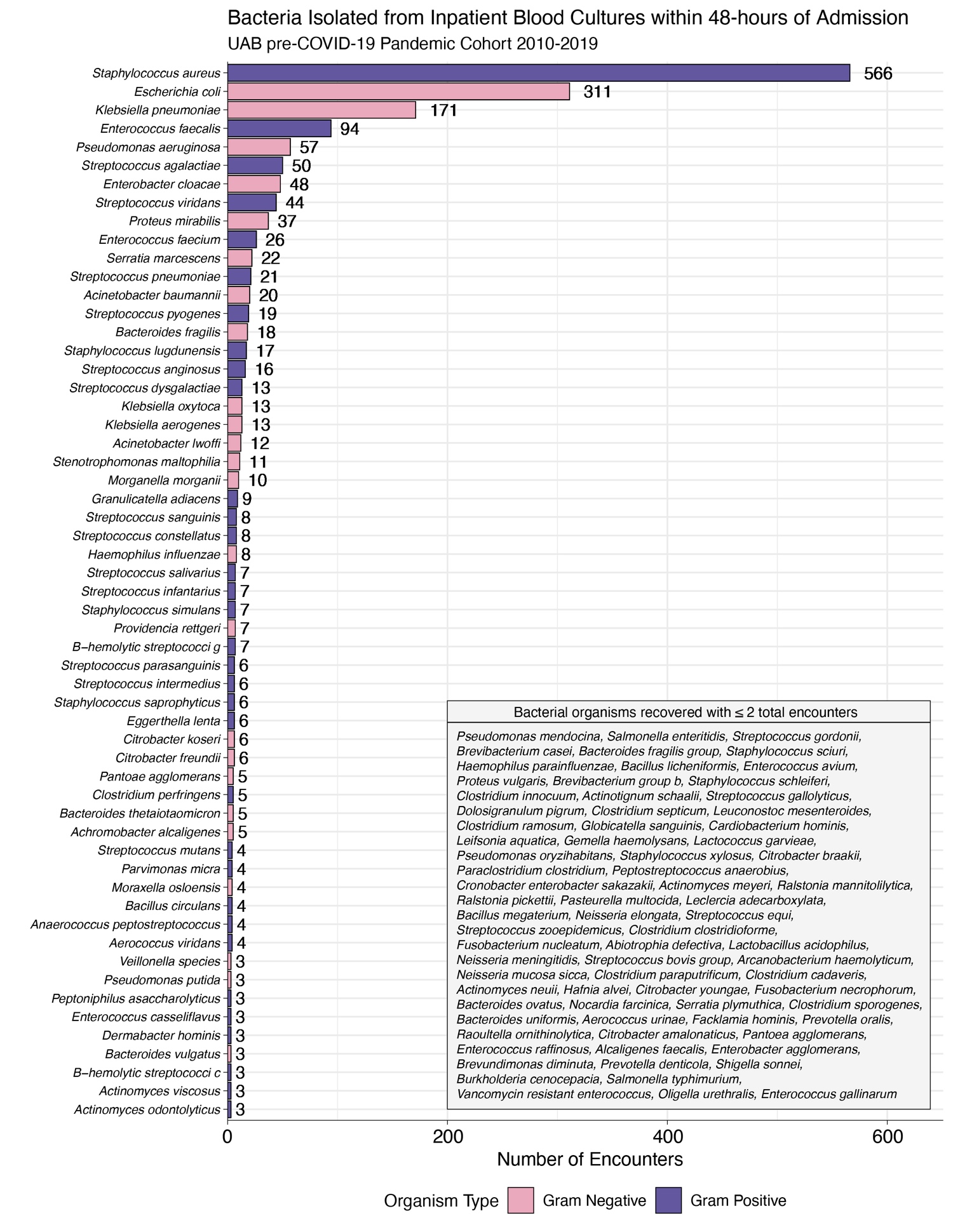


**eFigure 9: Bacterial isolated from inpatient blood cultures taken within 48-hours of admission in the UAB pre-COVID-19 pandemic cohort (2010-2019)**

| **eTable 1: Overall Comparison of UAB and OLHS Cohort Characteristics** | | | | |
| --- | --- | --- | --- | --- |
|  | Overall N = 13781^1^ | UAB Cohort N = 4075 | OLHS Cohort N = 9706 | p-value |
| Demographics |  |  |  |  |
| COVID-19+ Co-infection Status, n (%) |  |  |  | <0.001^2^ |
| Blood Culture(+) | 350 (2.5) | 110 (2.7) | 240 (2.5) |  |
| Blood Culture(-) | 6,351 (46) | 1,286 (32) | 5,065 (52) |  |
| No Blood Culture | 7,080 (51) | 2,679 (66) | 4,401 (45) |  |
| Age, Mean (SD) | 60 (17) | 58 (17) | 61 (17) | <0.001^3^ |
| Sex, n (%) |  |  |  | 0.68^2^ |
| Female | 6,963 (51) | 2,070 (51) | 4,893 (50) |  |
| Male | 6,818 (49) | 2,005 (49) | 4,813 (50) |  |
| Race, n (%) |  |  |  |  |
| White or Caucasian | 7,217 (52) | 1,927 (47) | 5,290 (55) |  |
| Black or African American | 5,690 (41) | 1,775 (44) | 3,915 (40) |  |
| Asian | 197 (1.4) | 116 (2.8) | 81 (0.8) |  |
| Hispanic or Latino | 145 (1.1) | 145 (3.6) | 0 (0) |  |
| American Indian or Alaska Native | 60 (0.4) | 8 (0.2) | 52 (0.5) |  |
| Pacific Islander or Hawaiian Native | 3 (<0.1) | 1 (<0.1) | 2 (<0.1) |  |
| Multiple/Other | 202 (1.5) | 5 (0.1) | 197 (2.0) |  |
| Decline/Refuse | 114 (0.8) | 98 (2.4) | 16 (0.2) |  |
| Race Unknown | 153 (1.1) | 0 (0) | 153 (1.6) |  |
| Charlson Comorbidity Score, Median (IQR) | 1 (0 – 3) | 2 (0 – 4) | 1 (0 – 3) | <0.001^3^ |
| Unknown | 2,113 | 1,146 | 967 |  |
| Inpatient Outcomes |  |  |  |  |
| Inpatient Length of Stay (Days), Median (IQR) | 5 (3 – 9) | 6 (3 – 11) | 5 (3 – 9) | <0.001^3^ |
| SIRS Score (within 24-hours; max=4), Median (IQR) | 2 (1 – 3) | 1 (1 – 2) | 2 (1 – 3) | <0.001^3^ |
| Unknown | 535 | 101 | 434 |  |
| In-Hospital Mortality Status, n (%) |  |  |  | <0.001^2^ |
| In-Hospital Deceased | 1,395 (10) | 488 (12) | 907 (9.3) |  |
| Discharged Living | 12,386 (90) | 3,587 (88) | 8,799 (91) |  |
| Mortality Status (30-day), n (%) |  |  |  | <0.001^2^ |
| Deceased (30-day) | 1,277 (9.3) | 433 (11) | 844 (8.7) |  |
| Living (30-day) | 12,504 (91) | 3,642 (89) | 8,862 (91) |  |
| ICU Status (anytime), n (%) |  |  |  | <0.001^2^ |
| ICU Admission | 4,737 (34) | 1,267 (31) | 3,470 (36) |  |
| No ICU Admission | 9,044 (66) | 2,808 (69) | 6,236 (64) |  |
| Mechanical Ventilation (anytime), n (%) |  |  |  | <0.001^2^ |
| Required Ventilation | 1,898 (14) | 713 (17) | 1,185 (12) |  |
| No Ventilation | 11,883 (86) | 3,362 (83) | 8,521 (88) |  |
| Inpatient Therapeutics |  |  |  |  |
| Antimicrobials (within 48-hours), n (%) |  |  |  | <0.001^2^ |
| Received Antimicrobial (within 48-hrs) | 8,804 (64) | 2,076 (51) | 6,728 (69) |  |
| No Antimicrobial (within 48-hrs) | 4,977 (36) | 1,999 (49) | 2,978 (31) |  |
| Dexamethasone (within 48-hours), n (%) |  |  |  | <0.001^2^ |
| Received Dexamethasone (within 48-hrs) | 6,344 (46) | 2,130 (52) | 4,214 (43) |  |
| No Dexamethasone (within 48-hrs) | 7,437 (54) | 1,945 (48) | 5,492 (57) |  |
| Pre-admission Comorbidities |  |  |  |  |
| Diabetic, n (%) | 4,227 (36) | 1,190 (41) | 3,037 (35) | <0.001^2^ |
| Heart Failure or MI, n (%) | 2,831 (24) | 821 (28) | 2,010 (23) | <0.001^2^ |
| Chronic pulmonary disease, n (%) | 2,907 (25) | 806 (28) | 2,101 (24) | <0.001^2^ |
| Renal disease, n (%) | 2,790 (24) | 774 (26) | 2,016 (23) | <0.001^2^ |
| Liver disease, n (%) | 1,107 (9.5) | 391 (13) | 716 (8.2) | <0.001^2^ |
| Vascular disease, n (%) | 2,996 (26) | 690 (24) | 2,306 (26) | 0.002^2^ |
| Cancer (any malignancy), n (%) | 1,336 (11) | 442 (15) | 894 (10) | <0.001^2^ |
| Peptic ulcer disease, n (%) | 393 (3.4) | 157 (5.4) | 236 (2.7) | <0.001^2^ |
| Hemiplegia or paraplegia, n (%) | 299 (2.6) | 121 (4.1) | 178 (2.0) | <0.001^2^ |
| Rheumatoid disease, n (%) | 460 (3.9) | 160 (5.5) | 300 (3.4) | <0.001^2^ |
| Dementia, n (%) | 576 (4.9) | 176 (6.0) | 400 (4.6) | 0.002^2^ |
| AIDS/HIV, n (%) | 129 (1.1) | 52 (1.8) | 77 (0.9) | <0.001^2^ |
| ^1^n (%); Mean (SD); Median (IQR) | | | | |
| ^2^Pearson's Chi-squared test | | | | |
| ^3^Wilcoxon rank sum test | | | | |

| **eTable 2: OLHS Cohort In-Hospital Mortality Models** | | | | | | |
| --- | --- | --- | --- | --- | --- | --- |
| Characteristic | Encounters Total N | Deaths, N (% of Total) | In-Hospital Mortality Unadjusted OR (95% CI)^1,2^ | p-value^3^ | In-Hospital Mortality Adjusted OR (95% CI)^2,4^ | p-value^3^ |
| Age Range |  |  |  | <0.001 |  | <0.001 |
| <65 years | 4,368 | 254 (5.8) | — |  | — |  |
| ≥65 years | 4,044 | 584 (14) | 2.72 (2.35 - 3.21) |  | 2.54 (2.17 - 2.98) |  |
| Diabetes History |  |  |  | <0.001 |  | 0.77 |
| No Diabetic History | 5,465 | 497 (9.1) | — |  | — |  |
| Diabetic History | 2,947 | 341 (12) | 1.31 (1.12 - 1.50) |  | 1.02 (0.88 - 1.20) |  |
| COPD History |  |  |  | 0.025 |  | 0.48 |
| No COPD History | 6,388 | 610 (9.5) | — |  | — |  |
| COPD History | 2,024 | 228 (11) | 1.20 (1.01 - 1.41) |  | 0.93 (0.79 - 1.10) |  |
| Heart Failure or MI History |  |  |  | <0.001 |  | <0.001 |
| No Cardiac History | 6,458 | 567 (8.8) | — |  | — |  |
| Heart Failure or MI History | 1,954 | 271 (14) | 1.66 (1.41 - 1.96) |  | 1.44 (1.23 - 1.72) |  |
| Renal Disease History |  |  |  | <0.001 |  | 0.94 |
| No Renal History | 6,427 | 587 (9.1) | — |  | — |  |
| Renal Disease History | 1,985 | 251 (13) | 1.44 (1.23 - 1.69) |  | 0.99 (0.82 - 1.19) |  |
| Sex |  |  |  | <0.001 |  | <0.001 |
| Female | 4,343 | 360 (8.3) | — |  | — |  |
| Male | 4,069 | 478 (12) | 1.47 (1.28 - 1.69) |  | 1.41 (1.22 - 1.62) |  |
| First 24-hour SIRS Score |  |  |  | <0.001 |  | <0.001 |
| <2 SIRS Score | 2,567 | 128 (5.0) | — |  | — |  |
| ≥2 SIRS Score | 5,845 | 710 (12) | 2.65 (2.20 - 3.24) |  | 2.44 (2.01 - 3.00) |  |
| COVID-19+ Co-infection Status |  |  |  | <0.001 |  | <0.001 |
| No Blood Culture, No Co-infection | 3,726 | 192 (5.2) | — |  | — |  |
| Blood Culture(-) Suspected Co-infection | 4,475 | 602 (13) | 2.85 (2.44 - 3.39) |  | 2.30 (1.94 - 2.77) |  |
| Blood Culture(+) Confirmed Co-infection | 211 | 44 (21) | 4.88 (3.31 - 6.86) |  | 3.70 (2.42 - 5.46) |  |
| ^1^Unadjusted Odds Ratio 95% confidence interval (n=1000 bootstraps) | | | | | | |
| ^2^OR = Odds Ratio, CI = Confidence Interval | | | | | | |
| ^3^Wald Test | | | | | | |
| ^4^Adjusted Odds Ratio 95% confidence interval (n=1000 bootstraps) | | | | | | |

| **eTable 3: UAB Cohort In-Hospital Mortality Models** | | | | | | |
| --- | --- | --- | --- | --- | --- | --- |
| Characteristic | Encounters Total N | Deaths, N (% of Total) | In-Hospital Mortality Unadjusted OR (95% CI)^1,2^ | p-value^3^ | In-Hospital Mortality Adjusted OR (95% CI)^2,4^ | p-value^3^ |
| Age Range |  |  |  | <0.001 |  | <0.001 |
| <65 years | 1,704 | 116 (6.8) | — |  | — |  |
| ≥65 years | 1,168 | 183 (16) | 2.56 (2.01 - 3.29) |  | 2.41 (1.85 - 3.15) |  |
| Diabetes History |  |  |  | <0.001 |  | 0.68 |
| No Diabetic History | 1,700 | 149 (8.8) | — |  | — |  |
| Diabetic History | 1,172 | 150 (13) | 1.52 (1.21 - 1.95) |  | 1.06 (0.80 - 1.39) |  |
| COPD History |  |  |  | 0.50 |  | 0.15 |
| No COPD History | 2,084 | 212 (10) | — |  | — |  |
| COPD History | 788 | 87 (11) | 1.09 (0.83 - 1.41) |  | 0.80 (0.59 - 1.10) |  |
| Heart Failure or MI History |  |  |  | <0.001 |  | 0.003 |
| No Cardiac History | 2,068 | 169 (8.2) | — |  | — |  |
| Heart Failure or MI History | 804 | 130 (16) | 2.16 (1.71 - 2.74) |  | 1.55 (1.17 - 2.10) |  |
| Renal Disease History |  |  |  | <0.001 |  | 0.022 |
| No Renal History | 2,113 | 177 (8.4) | — |  | — |  |
| Renal Disease History | 759 | 122 (16) | 2.09 (1.64 - 2.69) |  | 1.41 (1.06 - 1.89) |  |
| Sex |  |  |  | <0.001 |  | 0.010 |
| Female | 1,551 | 128 (8.3) | — |  | — |  |
| Male | 1,321 | 171 (13) | 1.66 (1.30 - 2.14) |  | 1.40 (1.08 - 1.86) |  |
| First 24-hour SIRS Score |  |  |  | <0.001 |  | <0.001 |
| <2 SIRS Score | 1,802 | 139 (7.7) | — |  | — |  |
| ≥2 SIRS Score | 1,070 | 160 (15) | 2.11 (1.62 - 2.69) |  | 1.70 (1.31 - 2.25) |  |
| COVID-19+ Co-infection Status |  |  |  | <0.001 |  | <0.001 |
| No Blood Culture, No Co-infection | 1,896 | 102 (5.4) | — |  | — |  |
| Blood Culture(-) Suspected Co-infection | 884 | 172 (19) | 4.24 (3.25 - 5.50) |  | 3.47 (2.64 - 4.60) |  |
| Blood Culture(+) Confirmed Co-infection | 92 | 25 (27) | 6.74 (3.98 - 10.7) |  | 4.05 (2.29 - 6.97) |  |
| ^1^Unadjusted Odds Ratio 95% confidence interval (n=1000 bootstraps) | | | | | | |
| ^2^OR = Odds Ratio, CI = Confidence Interval | | | | | | |
| ^3^Wald Test | | | | | | |
| ^4^Adjusted Odds Ratio 95% confidence interval (n=1000 bootstraps) | | | | | | |

| **eTable 4: Combined UAB and OLHS Cohorts In-Hospital Mortality Models** | | | | | | |
| --- | --- | --- | --- | --- | --- | --- |
| Characteristic | Encounters Total N | Deaths, N (% of Total) | In-Hospital Mortality Unadjusted OR (95% CI)^1,2^ | p-value^3^ | In-Hospital Mortality Adjusted OR (95% CI)^2,4^ | p-value^3^ |
| Age Range |  |  |  | <0.001 |  | <0.001 |
| <65 years | 6,072 | 370 (6.1) | — |  | — |  |
| ≥65 years | 5,212 | 767 (15) | 2.66 (2.33 - 3.05) |  | 2.36 (2.08 - 2.71) |  |
| Diabetes History |  |  |  | <0.001 |  | 0.48 |
| No Diabetic History | 7,165 | 646 (9.0) | — |  | — |  |
| Diabetic History | 4,119 | 491 (12) | 1.37 (1.20 - 1.55) |  | 1.05 (0.91 - 1.20) |  |
| COPD History |  |  |  | 0.022 |  | 0.24 |
| No COPD History | 8,472 | 822 (9.7) | — |  | — |  |
| COPD History | 2,812 | 315 (11) | 1.18 (1.02 - 1.34) |  | 0.92 (0.78 - 1.06) |  |
| Heart Failure or MI History |  |  |  | <0.001 |  | <0.001 |
| No Cardiac History | 8,526 | 736 (8.6) | — |  | — |  |
| Heart Failure or MI History | 2,758 | 401 (15) | 1.80 (1.58 - 2.06) |  | 1.52 (1.30 - 1.76) |  |
| Renal Disease History |  |  |  | <0.001 |  | 0.23 |
| No Renal History | 8,540 | 764 (8.9) | — |  | — |  |
| Renal Disease History | 2,744 | 373 (14) | 1.60 (1.41 - 1.83) |  | 1.10 (0.94 - 1.28) |  |
| Sex |  |  |  | <0.001 |  | <0.001 |
| Female | 5,894 | 488 (8.3) | — |  | — |  |
| Male | 5,390 | 649 (12) | 1.52 (1.34 - 1.74) |  | 1.41 (1.24 - 1.60) |  |
| First 24-hour SIRS Score |  |  |  | <0.001 |  | <0.001 |
| <2 SIRS Score | 4,369 | 267 (6.1) | — |  | — |  |
| ≥2 SIRS Score | 6,915 | 870 (13) | 2.21 (1.92 - 2.51) |  | 1.89 (1.64 - 2.19) |  |
| COVID-19+ Co-infection Status |  |  |  | <0.001 |  | <0.001 |
| No Blood Culture, No Co-infection | 5,622 | 294 (5.2) | — |  | — |  |
| Blood Culture(-) Suspected Co-infection | 5,359 | 774 (14) | 3.05 (2.67 - 3.52) |  | 2.42 (2.11 - 2.81) |  |
| Blood Culture(+) Confirmed Co-infection | 303 | 69 (23) | 5.32 (3.94 - 7.12) |  | 3.88 (2.80 - 5.21) |  |
| ^1^Unadjusted Odds Ratio 95% confidence interval (n=1000 bootstraps) | | | | | | |
| ^2^OR = Odds Ratio, CI = Confidence Interval | | | | | | |
| ^3^Wald Test | | | | | | |
| ^4^Adjusted Odds Ratio 95% confidence interval (n=1000 bootstraps) | | | | | | |

| **eTable 5: OLHS Cohort Mechanical Ventilation Models** | | | | | | |
| --- | --- | --- | --- | --- | --- | --- |
| Characteristic | Encounters Total N | Required Ventilation N (% of Total) | Required Ventilation Unadjusted OR (95% CI)^1,2^ | p-value^3^ | Required Ventilation Adjusted OR (95% CI)^2,4^ | p-value^3^ |
| Age Range |  |  |  | <0.001 |  | <0.001 |
| <65 years | 4,368 | 483 (11) | — |  | — |  |
| ≥65 years | 4,044 | 585 (14) | 1.36 (1.19 - 1.54) |  | 1.33 (1.15 - 1.51) |  |
| Diabetes History |  |  |  | 0.22 |  | 0.91 |
| No Diabetic History | 5,465 | 676 (12) | — |  | — |  |
| Diabetic History | 2,947 | 392 (13) | 1.09 (0.96 - 1.23) |  | 1.01 (0.87 - 1.17) |  |
| COPD History |  |  |  | 0.36 |  | 0.10 |
| No COPD History | 6,388 | 823 (13) | — |  | — |  |
| COPD History | 2,024 | 245 (12) | 0.93 (0.79 - 1.08) |  | 0.87 (0.74 - 1.02) |  |
| Heart Failure or MI History |  |  |  | 0.54 |  | 0.66 |
| No Cardiac History | 6,458 | 812 (13) | — |  | — |  |
| Heart Failure or MI History | 1,954 | 256 (13) | 1.04 (0.90 - 1.21) |  | 1.04 (0.86 - 1.22) |  |
| Renal Disease History |  |  |  | 0.28 |  | 0.90 |
| No Renal History | 6,427 | 802 (12) | — |  | — |  |
| Renal Disease History | 1,985 | 266 (13) | 1.09 (0.92 - 1.26) |  | 0.99 (0.83 - 1.18) |  |
| Sex |  |  |  | <0.001 |  | <0.001 |
| Female | 4,343 | 469 (11) | — |  | — |  |
| Male | 4,069 | 599 (15) | 1.42 (1.25 - 1.61) |  | 1.34 (1.18 - 1.54) |  |
| First 24-hour SIRS Score |  |  |  | <0.001 |  | <0.001 |
| <2 SIRS Score | 2,567 | 149 (5.8) | — |  | — |  |
| ≥2 SIRS Score | 5,845 | 919 (16) | 3.03 (2.53 - 3.67) |  | 2.61 (2.16 - 3.17) |  |
| COVID-19+ Co-infection Status |  |  |  | <0.001 |  | <0.001 |
| No Blood Culture, No Co-infection | 3,726 | 260 (7.0) | — |  | — |  |
| Blood Culture(-) Suspected Co-infection | 4,475 | 764 (17) | 2.74 (2.37 - 3.17) |  | 2.33 (1.99 - 2.71) |  |
| Blood Culture(+) Confirmed Co-infection | 211 | 44 (21) | 3.50 (2.38 - 4.91) |  | 2.75 (1.87 - 3.92) |  |
| ^1^Unadjusted Odds Ratio 95% confidence interval (n=1000 bootstraps) | | | | | | |
| ^2^OR = Odds Ratio, CI = Confidence Interval | | | | | | |
| ^3^Wald Test | | | | | | |
| ^4^Adjusted Odds Ratio 95% confidence interval (n=1000 bootstraps) | | | | | | |

| **eTable 6: UAB Cohort Mechanical Ventilation Models** | | | | | | |
| --- | --- | --- | --- | --- | --- | --- |
| Characteristic | Encounters Total N | Required Ventilation N (% of Total) | Required Ventilation Unadjusted OR (95% CI)^1,2^ | p-value^3^ | Required Ventilation Adjusted OR (95% CI)^2,4^ | p-value^3^ |
| Age Range |  |  |  | 0.28 |  | 0.58 |
| <65 years | 1,704 | 210 (12) | — |  | — |  |
| ≥65 years | 1,168 | 160 (14) | 1.12 (0.90 - 1.39) |  | 1.07 (0.83 - 1.40) |  |
| Diabetes History |  |  |  | 0.005 |  | 0.10 |
| No Diabetic History | 1,700 | 194 (11) | — |  | — |  |
| Diabetic History | 1,172 | 176 (15) | 1.37 (1.10 - 1.72) |  | 1.23 (0.96 - 1.59) |  |
| COPD History |  |  |  | 0.66 |  | 0.22 |
| No COPD History | 2,084 | 272 (13) | — |  | — |  |
| COPD History | 788 | 98 (12) | 0.94 (0.73 - 1.20) |  | 0.84 (0.64 - 1.12) |  |
| Heart Failure or MI History |  |  |  | 0.031 |  | 0.78 |
| No Cardiac History | 2,068 | 249 (12) | — |  | — |  |
| Heart Failure or MI History | 804 | 121 (15) | 1.29 (1.02 - 1.61) |  | 1.05 (0.79 - 1.38) |  |
| Renal Disease History |  |  |  | 0.002 |  | 0.45 |
| No Renal History | 2,113 | 247 (12) | — |  | — |  |
| Renal Disease History | 759 | 123 (16) | 1.48 (1.14 - 1.84) |  | 1.11 (0.83 - 1.44) |  |
| Sex |  |  |  | <0.001 |  | 0.009 |
| Female | 1,551 | 166 (11) | — |  | — |  |
| Male | 1,321 | 204 (15) | 1.51 (1.22 - 1.90) |  | 1.38 (1.08 - 1.71) |  |
| First 24-hour SIRS Score |  |  |  | <0.001 |  | <0.001 |
| <2 SIRS Score | 1,802 | 164 (9.1) | — |  | — |  |
| ≥2 SIRS Score | 1,070 | 206 (19) | 2.37 (1.90 - 2.93) |  | 1.66 (1.33 - 2.14) |  |
| COVID-19+ Co-infection Status |  |  |  | <0.001 |  | <0.001 |
| No Blood Culture, No Co-infection | 1,896 | 118 (6.2) | — |  | — |  |
| Blood Culture(-) Suspected Co-infection | 884 | 228 (26) | 5.23 (4.11 - 6.64) |  | 4.42 (3.50 - 5.72) |  |
| Blood Culture(+) Confirmed Co-infection | 92 | 24 (26) | 5.21 (3.08 - 8.56) |  | 3.84 (2.21 - 6.12) |  |
| ^1^Unadjusted Odds Ratio 95% confidence interval (n=1000 bootstraps) | | | | | | |
| ^2^OR = Odds Ratio, CI = Confidence Interval | | | | | | |
| ^3^Wald Test | | | | | | |
| ^4^Adjusted Odds Ratio 95% confidence interval (n=1000 bootstraps) | | | | | | |

| **eTable 7: Combined UAB and OLHS Cohorts Mechanical Ventilation Models** | | | | | | |
| --- | --- | --- | --- | --- | --- | --- |
| Characteristic | Encounters Total N | Required Ventilation N (% of Total) | Required Ventilation Unadjusted OR (95% CI)^1,2^ | p-value^3^ | Required Ventilation Adjusted OR (95% CI)^2,4^ | p-value^3^ |
| Age Range |  |  |  | <0.001 |  | 0.004 |
| <65 years | 6,072 | 693 (11) | — |  | — |  |
| ≥65 years | 5,212 | 745 (14) | 1.30 (1.16 - 1.44) |  | 1.19 (1.05 - 1.33) |  |
| Diabetes History |  |  |  | 0.012 |  | 0.24 |
| No Diabetic History | 7,165 | 870 (12) | — |  | — |  |
| Diabetic History | 4,119 | 568 (14) | 1.16 (1.04 - 1.29) |  | 1.08 (0.95 - 1.23) |  |
| COPD History |  |  |  | 0.32 |  | 0.074 |
| No COPD History | 8,472 | 1095 (13) | — |  | — |  |
| COPD History | 2,812 | 343 (12) | 0.94 (0.83 - 1.06) |  | 0.88 (0.76 - 1.01) |  |
| Heart Failure or MI History |  |  |  | 0.094 |  | 0.36 |
| No Cardiac History | 8,526 | 1061 (12) | — |  | — |  |
| Heart Failure or MI History | 2,758 | 377 (14) | 1.11 (0.98 - 1.26) |  | 1.07 (0.91 - 1.23) |  |
| Renal Disease History |  |  |  | 0.010 |  | 0.57 |
| No Renal History | 8,540 | 1049 (12) | — |  | — |  |
| Renal Disease History | 2,744 | 389 (14) | 1.18 (1.03 - 1.34) |  | 1.04 (0.91 - 1.20) |  |
| Sex |  |  |  | <0.001 |  | <0.001 |
| Female | 5,894 | 635 (11) | — |  | — |  |
| Male | 5,390 | 803 (15) | 1.45 (1.30 - 1.63) |  | 1.34 (1.20 - 1.51) |  |
| First 24-hour SIRS Score |  |  |  | <0.001 |  | <0.001 |
| <2 SIRS Score | 4,369 | 313 (7.2) | — |  | — |  |
| ≥2 SIRS Score | 6,915 | 1125 (16) | 2.52 (2.21 - 2.86) |  | 2.00 (1.77 - 2.31) |  |
| COVID-19+ Co-infection Status |  |  |  | <0.001 |  | <0.001 |
| No Blood Culture, No Co-infection | 5,622 | 378 (6.7) | — |  | — |  |
| Blood Culture(-) Suspected Co-infection | 5,359 | 992 (19) | 3.15 (2.78 - 3.59) |  | 2.63 (2.30 - 2.98) |  |
| Blood Culture(+) Confirmed Co-infection | 303 | 68 (22) | 4.00 (2.98 - 5.36) |  | 3.11 (2.29 - 4.05) |  |
| ^1^Unadjusted Odds Ratio 95% confidence interval (n=1000 bootstraps) | | | | | | |
| ^2^OR = Odds Ratio, CI = Confidence Interval | | | | | | |
| ^3^Wald Test | | | | | | |
| ^4^Adjusted Odds Ratio 95% confidence interval (n=1000 bootstraps) | | | | | | |

| **eTable 8: OLHS Cohort ICU Admission Models** | | | | | | |
| --- | --- | --- | --- | --- | --- | --- |
| Characteristic | Encounters Total N | ICU Admissions N (% of Total) | ICU Admission Unadjusted OR (95% CI)^1,2^ | p-value^3^ | ICU Admission Adjusted OR (95% CI)^2,4^ | p-value^3^ |
| Age Range |  |  |  | <0.001 |  | <0.001 |
| <65 years | 4,368 | 1489 (34) | — |  | — |  |
| ≥65 years | 4,044 | 1620 (40) | 1.30 (1.18 - 1.41) |  | 1.20 (1.09 - 1.31) |  |
| Diabetes History |  |  |  | <0.001 |  | 0.012 |
| No Diabetic History | 5,465 | 1916 (35) | — |  | — |  |
| Diabetic History | 2,947 | 1193 (40) | 1.26 (1.15 - 1.38) |  | 1.14 (1.02 - 1.25) |  |
| COPD History |  |  |  | 0.96 |  | 0.11 |
| No COPD History | 6,388 | 2360 (37) | — |  | — |  |
| COPD History | 2,024 | 749 (37) | 1.00 (0.91 - 1.10) |  | 0.91 (0.82 - 1.03) |  |
| Heart Failure or MI History |  |  |  | <0.001 |  | 0.054 |
| No Cardiac History | 6,458 | 2319 (36) | — |  | — |  |
| Heart Failure or MI History | 1,954 | 790 (40) | 1.21 (1.09 - 1.33) |  | 1.12 (1.00 - 1.26) |  |
| Renal Disease History |  |  |  | <0.001 |  | 0.30 |
| No Renal History | 6,427 | 2298 (36) | — |  | — |  |
| Renal Disease History | 1,985 | 811 (41) | 1.24 (1.12 - 1.38) |  | 1.07 (0.95 - 1.21) |  |
| Sex |  |  |  | <0.001 |  | <0.001 |
| Female | 4,343 | 1466 (34) | — |  | — |  |
| Male | 4,069 | 1643 (40) | 1.33 (1.22 - 1.45) |  | 1.27 (1.15 - 1.39) |  |
| First 24-hour SIRS Score |  |  |  | <0.001 |  | <0.001 |
| <2 SIRS Score | 2,567 | 693 (27) | — |  | — |  |
| ≥2 SIRS Score | 5,845 | 2416 (41) | 1.91 (1.74 - 2.11) |  | 1.72 (1.56 - 1.90) |  |
| COVID-19+ Co-infection Status |  |  |  | <0.001 |  | <0.001 |
| No Blood Culture, No Co-infection | 3,726 | 1056 (28) | — |  | — |  |
| Blood Culture(-) Suspected Co-infection | 4,475 | 1935 (43) | 1.93 (1.76 - 2.12) |  | 1.70 (1.54 - 1.86) |  |
| Blood Culture(+) Confirmed Co-infection | 211 | 118 (56) | 3.20 (2.44 - 4.27) |  | 2.65 (2.00 - 3.48) |  |
| ^1^Unadjusted Odds Ratio 95% confidence interval (n=1000 bootstraps) | | | | | | |
| ^2^OR = Odds Ratio, CI = Confidence Interval | | | | | | |
| ^3^Wald Test | | | | | | |
| ^4^Adjusted Odds Ratio 95% confidence interval (n=1000 bootstraps) | | | | | | |

| **eTable 9: UAB Cohort ICU Admission Models** | | | | | | |
| --- | --- | --- | --- | --- | --- | --- |
| Characteristic | Encounters Total N | ICU Admissions N (% of Total) | ICU Admission Unadjusted OR (95% CI)^1,2^ | p-value^3^ | ICU Admission Adjusted OR (95% CI)^2,4^ | p-value^3^ |
| Age Range |  |  |  | 0.002 |  | 0.016 |
| <65 years | 1,704 | 409 (24) | — |  | — |  |
| ≥65 years | 1,168 | 342 (29) | 1.31 (1.12 - 1.55) |  | 1.26 (1.02 - 1.50) |  |
| Diabetes History |  |  |  | 0.003 |  | 0.42 |
| No Diabetic History | 1,700 | 410 (24) | — |  | — |  |
| Diabetic History | 1,172 | 341 (29) | 1.29 (1.08 - 1.52) |  | 1.08 (0.90 - 1.32) |  |
| COPD History |  |  |  | 0.64 |  | 0.27 |
| No COPD History | 2,084 | 540 (26) | — |  | — |  |
| COPD History | 788 | 211 (27) | 1.05 (0.87 - 1.25) |  | 0.89 (0.72 - 1.11) |  |
| Heart Failure or MI History |  |  |  | <0.001 |  | 0.010 |
| No Cardiac History | 2,068 | 489 (24) | — |  | — |  |
| Heart Failure or MI History | 804 | 262 (33) | 1.57 (1.30 - 1.87) |  | 1.33 (1.05 - 1.67) |  |
| Renal Disease History |  |  |  | <0.001 |  | 0.81 |
| No Renal History | 2,113 | 511 (24) | — |  | — |  |
| Renal Disease History | 759 | 240 (32) | 1.45 (1.19 - 1.73) |  | 1.03 (0.83 - 1.28) |  |
| Sex |  |  |  | <0.001 |  | <0.001 |
| Female | 1,551 | 343 (22) | — |  | — |  |
| Male | 1,321 | 408 (31) | 1.57 (1.33 - 1.85) |  | 1.43 (1.19 - 1.74) |  |
| First 24-hour SIRS Score |  |  |  | <0.001 |  | <0.001 |
| <2 SIRS Score | 1,802 | 356 (20) | — |  | — |  |
| ≥2 SIRS Score | 1,070 | 395 (37) | 2.37 (1.99 - 2.82) |  | 1.75 (1.46 - 2.13) |  |
| COVID-19+ Co-infection Status |  |  |  | <0.001 |  | <0.001 |
| No Blood Culture, No Co-infection | 1,896 | 289 (15) | — |  | — |  |
| Blood Culture(-) Suspected Co-infection | 884 | 413 (47) | 4.87 (4.07 - 5.90) |  | 4.14 (3.46 - 4.97) |  |
| Blood Culture(+) Confirmed Co-infection | 92 | 49 (53) | 6.34 (4.13 - 9.82) |  | 4.47 (2.87 - 7.09) |  |
| ^1^Unadjusted Odds Ratio 95% confidence interval (n=1000 bootstraps) | | | | | | |
| ^2^OR = Odds Ratio, CI = Confidence Interval | | | | | | |
| ^3^Wald Test | | | | | | |
| ^4^Adjusted Odds Ratio 95% confidence interval (n=1000 bootstraps) | | | | | | |

| **eTable 10: Combined UAB and OLHS Cohorts ICU Admission Models** | | | | | | |
| --- | --- | --- | --- | --- | --- | --- |
| Characteristic | Encounters Total N | ICU Admissions N (% of Total) | ICU Admission Unadjusted OR (95% CI)^1,2^ | p-value^3^ | ICU Admission Adjusted OR (95% CI)^2,4^ | p-value^3^ |
| Age Range |  |  |  | <0.001 |  | <0.001 |
| <65 years | 6,072 | 1898 (31) | — |  | — |  |
| ≥65 years | 5,212 | 1962 (38) | 1.33 (1.23 - 1.43) |  | 1.21 (1.11 - 1.31) |  |
| Diabetes History |  |  |  | <0.001 |  | 0.017 |
| No Diabetic History | 7,165 | 2326 (32) | — |  | — |  |
| Diabetic History | 4,119 | 1534 (37) | 1.24 (1.15 - 1.33) |  | 1.11 (1.02 - 1.22) |  |
| COPD History |  |  |  | 0.93 |  | 0.040 |
| No COPD History | 8,472 | 2900 (34) | — |  | — |  |
| COPD History | 2,812 | 960 (34) | 0.99 (0.91 - 1.09) |  | 0.90 (0.82 - 0.99) |  |
| Heart Failure or MI History |  |  |  | <0.001 |  | 0.002 |
| No Cardiac History | 8,526 | 2808 (33) | — |  | — |  |
| Heart Failure or MI History | 2,758 | 1052 (38) | 1.26 (1.15 - 1.37) |  | 1.17 (1.07 - 1.31) |  |
| Renal Disease History |  |  |  | <0.001 |  | 0.22 |
| No Renal History | 8,540 | 2809 (33) | — |  | — |  |
| Renal Disease History | 2,744 | 1051 (38) | 1.26 (1.15 - 1.38) |  | 1.07 (0.97 - 1.18) |  |
| Sex |  |  |  | <0.001 |  | <0.001 |
| Female | 5,894 | 1809 (31) | — |  | — |  |
| Male | 5,390 | 2051 (38) | 1.38 (1.28 - 1.49) |  | 1.30 (1.20 - 1.41) |  |
| First 24-hour SIRS Score |  |  |  | <0.001 |  | <0.001 |
| <2 SIRS Score | 4,369 | 1049 (24) | — |  | — |  |
| ≥2 SIRS Score | 6,915 | 2811 (41) | 2.17 (2.00 - 2.37) |  | 1.83 (1.68 - 2.00) |  |
| COVID-19+ Co-infection Status |  |  |  | <0.001 |  | <0.001 |
| No Blood Culture, No Co-infection | 5,622 | 1345 (24) | — |  | — |  |
| Blood Culture(-) Suspected Co-infection | 5,359 | 2348 (44) | 2.48 (2.29 - 2.69) |  | 2.09 (1.91 - 2.29) |  |
| Blood Culture(+) Confirmed Co-infection | 303 | 167 (55) | 3.90 (3.08 - 4.93) |  | 3.02 (2.36 - 3.94) |  |
| ^1^Unadjusted Odds Ratio 95% confidence interval (n=1000 bootstraps) | | | | | | |
| ^2^OR = Odds Ratio, CI = Confidence Interval | | | | | | |
| ^3^Wald Test | | | | | | |
| ^4^Adjusted Odds Ratio 95% confidence interval (n=1000 bootstraps) | | | | | | |

| **eTable 11: Combined UAB and OLHS Cohorts In-Hospital Mortality Models during COVID-19 Alpha-Variant Wave (03/01/2020-05/31/2021)** | | | | | | |
| --- | --- | --- | --- | --- | --- | --- |
| Characteristic | Encounters Total N | Deaths, N (% of Total) | In-Hospital Mortality Unadjusted OR (95% CI)^1,2^ | p-value^3^ | In-Hospital Mortality Adjusted OR (95% CI)^2,4^ | p-value^3^ |
| Age Range |  |  |  | <0.001 |  | <0.001 |
| <65 years | 3,431 | 209 (6.1) | — |  | — |  |
| ≥65 years | 3,472 | 556 (16) | 2.96 (2.53 - 3.54) |  | 2.58 (2.16 - 3.07) |  |
| Diabetes History |  |  |  | <0.001 |  | 0.95 |
| No Diabetic History | 4,205 | 421 (10) | — |  | — |  |
| Diabetic History | 2,698 | 344 (13) | 1.31 (1.14 - 1.53) |  | 0.99 (0.85 - 1.17) |  |
| COPD History |  |  |  | <0.001 |  | 0.81 |
| No COPD History | 5,191 | 538 (10) | — |  | — |  |
| COPD History | 1,712 | 227 (13) | 1.32 (1.11 - 1.54) |  | 1.03 (0.85 - 1.22) |  |
| Heart Failure or MI History |  |  |  | <0.001 |  | <0.001 |
| No Cardiac History | 5,168 | 473 (9.2) | — |  | — |  |
| Heart Failure or MI History | 1,735 | 292 (17) | 2.01 (1.72 - 2.34) |  | 1.72 (1.43 - 2.09) |  |
| Renal Disease History |  |  |  | <0.001 |  | 0.19 |
| No Renal History | 5,111 | 492 (9.6) | — |  | — |  |
| Renal Disease History | 1,792 | 273 (15) | 1.69 (1.45 - 1.97) |  | 1.13 (0.93 - 1.36) |  |
| Sex |  |  |  | <0.001 |  | <0.001 |
| Female | 3,540 | 309 (8.7) | — |  | — |  |
| Male | 3,363 | 456 (14) | 1.65 (1.42 - 1.91) |  | 1.48 (1.26 - 1.74) |  |
| First 24-hour SIRS Score |  |  |  | <0.001 |  | <0.001 |
| <2 SIRS Score | 2,809 | 185 (6.6) | — |  | — |  |
| ≥2 SIRS Score | 4,094 | 580 (14) | 2.34 (1.99 - 2.79) |  | 1.95 (1.63 - 2.32) |  |
| COVID-19+ Co-infection Status |  |  |  | <0.001 |  | <0.001 |
| No Blood Culture, No Co-infection | 2,995 | 145 (4.8) | — |  | — |  |
| Blood Culture(-) Suspected Co-infection | 3,724 | 576 (15) | 3.58 (2.98 - 4.32) |  | 2.78 (2.29 - 3.36) |  |
| Blood Culture(+) Confirmed Co-infection | 184 | 44 (24) | 6.20 (4.13 - 8.79) |  | 4.12 (2.71 - 6.04) |  |
| ^1^Unadjusted Odds Ratio 95% confidence interval (n=1000 bootstraps) | | | | | | |
| ^2^OR = Odds Ratio, CI = Confidence Interval | | | | | | |
| ^3^Wald Test | | | | | | |
| ^4^Adjusted Odds Ratio 95% confidence interval (n=1000 bootstraps) | | | | | | |

| **eTable 12: Combined UAB and OLHS Cohorts In-Hospital Mortality Models during COVID-19 Delta-Variant Wave (06/01/2021-01/01/2022)** | | | | | | |
| --- | --- | --- | --- | --- | --- | --- |
| Characteristic | Encounters Total N | Deaths, N (% of Total) | In-Hospital Mortality Unadjusted OR (95% CI)^1,2^ | p-value^3^ | In-Hospital Mortality Adjusted OR (95% CI)^2,4^ | p-value^3^ |
| Age Range |  |  |  | <0.001 |  | <0.001 |
| <65 years | 1,996 | 137 (6.9) | — |  | — |  |
| ≥65 years | 1,158 | 160 (14) | 2.17 (1.69 - 2.79) |  | 2.09 (1.65 - 2.74) |  |
| Diabetes History |  |  |  | 0.002 |  | 0.11 |
| No Diabetic History | 2,171 | 181 (8.3) | — |  | — |  |
| Diabetic History | 983 | 116 (12) | 1.48 (1.15 - 1.87) |  | 1.25 (0.93 - 1.61) |  |
| COPD History |  |  |  | 0.95 |  | 0.075 |
| No COPD History | 2,438 | 230 (9.4) | — |  | — |  |
| COPD History | 716 | 67 (9.4) | 0.98 (0.73 - 1.29) |  | 0.75 (0.55 - 1.01) |  |
| Heart Failure or MI History |  |  |  | <0.001 |  | 0.038 |
| No Cardiac History | 2,524 | 214 (8.5) | — |  | — |  |
| Heart Failure or MI History | 630 | 83 (13) | 1.63 (1.26 - 2.12) |  | 1.39 (1.00 - 1.85) |  |
| Renal Disease History |  |  |  | 0.040 |  | 0.57 |
| No Renal History | 2,542 | 226 (8.9) | — |  | — |  |
| Renal Disease History | 612 | 71 (12) | 1.35 (0.99 - 1.74) |  | 0.91 (0.65 - 1.25) |  |
| Sex |  |  |  | 0.10 |  | 0.13 |
| Female | 1,683 | 145 (8.6) | — |  | — |  |
| Male | 1,471 | 152 (10) | 1.23 (0.98 - 1.55) |  | 1.21 (0.94 - 1.54) |  |
| First 24-hour SIRS Score |  |  |  | <0.001 |  | 0.004 |
| <2 SIRS Score | 1,074 | 74 (6.9) | — |  | — |  |
| ≥2 SIRS Score | 2,080 | 223 (11) | 1.61 (1.25 - 2.08) |  | 1.53 (1.19 - 2.09) |  |
| COVID-19+ Co-infection Status |  |  |  | <0.001 |  | <0.001 |
| No Blood Culture, No Co-infection | 1,866 | 126 (6.8) | — |  | — |  |
| Blood Culture(-) Suspected Co-infection | 1,219 | 156 (13) | 2.02 (1.59 - 2.61) |  | 1.80 (1.40 - 2.38) |  |
| Blood Culture(+) Confirmed Co-infection | 69 | 15 (22) | 3.81 (1.92 - 6.64) |  | 3.23 (1.65 - 5.74) |  |
| ^1^Unadjusted Odds Ratio 95% confidence interval (n=1000 bootstraps) | | | | | | |
| ^2^OR = Odds Ratio, CI = Confidence Interval | | | | | | |
| ^3^Wald Test | | | | | | |
| ^4^Adjusted Odds Ratio 95% confidence interval (n=1000 bootstraps) | | | | | | |

| **eTable 13: Combined UAB and OLHS Cohorts In-Hospital Mortality Models during COVID-19 Omicron-Variant Wave (01/02/2022- 03/02/2022)** | | | | | | |
| --- | --- | --- | --- | --- | --- | --- |
| Characteristic | Encounters Total N | Deaths, N (% of Total) | In-Hospital Mortality Unadjusted OR (95% CI)^1,2^ | p-value^3^ | In-Hospital Mortality Adjusted OR (95% CI)^2,4^ | p-value^3^ |
| Age Range |  |  |  | <0.001 |  | 0.005 |
| <65 years | 645 | 24 (3.7) | — |  | — |  |
| ≥65 years | 582 | 51 (8.8) | 2.49 (1.58 - 4.26) |  | 2.14 (1.29 - 3.71) |  |
| Diabetes History |  |  |  | 0.29 |  | 0.95 |
| No Diabetic History | 789 | 44 (5.6) | — |  | — |  |
| Diabetic History | 438 | 31 (7.1) | 1.30 (0.80 - 2.06) |  | 0.96 (0.56 - 1.69) |  |
| COPD History |  |  |  | 0.53 |  | 0.22 |
| No COPD History | 843 | 54 (6.4) | — |  | — |  |
| COPD History | 384 | 21 (5.5) | 0.84 (0.47 - 1.44) |  | 0.70 (0.39 - 1.24) |  |
| Heart Failure or MI History |  |  |  | 0.61 |  | 0.73 |
| No Cardiac History | 834 | 49 (5.9) | — |  | — |  |
| Heart Failure or MI History | 393 | 26 (6.6) | 1.14 (0.66 - 1.81) |  | 0.89 (0.50 - 1.57) |  |
| Renal Disease History |  |  |  | 0.030 |  | 0.14 |
| No Renal History | 887 | 46 (5.2) | — |  | — |  |
| Renal Disease History | 340 | 29 (8.5) | 1.67 (1.03 - 2.68) |  | 1.53 (0.88 - 2.56) |  |
| Sex |  |  |  | 0.095 |  | 0.20 |
| Female | 671 | 34 (5.1) | — |  | — |  |
| Male | 556 | 41 (7.4) | 1.51 (0.92 - 2.54) |  | 1.39 (0.85 - 2.37) |  |
| First 24-hour SIRS Score |  |  |  | <0.001 |  | <0.001 |
| <2 SIRS Score | 486 | 8 (1.6) | — |  | — |  |
| ≥2 SIRS Score | 741 | 67 (9.0) | 6.02 (3.15 - 16.4) |  | 4.25 (2.14 - 11.5) |  |
| COVID-19+ Co-infection Status |  |  |  | <0.001 |  | <0.001 |
| No Blood Culture, No Co-infection | 761 | 23 (3.0) | — |  | — |  |
| Blood Culture(-) Suspected Co-infection | 416 | 42 (10) | 3.73 (2.20 - 6.13) |  | 2.69 (1.56 - 4.92) |  |
| Blood Culture(+) Confirmed Co-infection | 50 | 10 (20) | 8.06 (3.04 - 17.7) |  | 5.49 (1.98 - 13.8) |  |
| ^1^Unadjusted Odds Ratio 95% confidence interval (n=1000 bootstraps) | | | | | | |
| ^2^OR = Odds Ratio, CI = Confidence Interval | | | | | | |
| ^3^Wald Test | | | | | | |
| ^4^Adjusted Odds Ratio 95% confidence interval (n=1000 bootstraps) | | | | | | |

| **eTable 14: Characteristics, outcomes, and therapeutics for inpatient encounters with confirmed, suspected, and no community acquired bacteremic infections in the UAB pre-COVID-19 pandemic cohort (2010-2019)** | | | |
| --- | --- | --- | --- |
|  | UAB Cohort (2010-2019) | | |
|  | Confirmed  Blood Culture(+),  N = 1,703 | Suspected  Blood Culture(-),  N = 11,795 | None  No Blood Culture,  N = 85,672 |
| Age, Median (IQR) | 60 (46 – 69) | 59 (45 – 69) | 59 (42 – 70) |
| Sex, n (%) |  |  |  |
| Female | 760 (45) | 5,621 (48) | 46,306 (54) |
| Male | 943 (55) | 6,174 (52) | 39,366 (46) |
| Race, n (%) |  |  |  |
| White or Caucasian | 913 (54) | 6,627 (56) | 46,848 (55) |
| Black or African American | 703 (41) | 4,654 (39) | 34,787 (41) |
| Asian | 34 (2.0) | 236 (2.0) | 1,654 (1.9) |
| Hispanic or Latino | 31 (1.8) | 185 (1.6) | 1,551 (1.8) |
| American Indian or Alaska Native | 9 (0.5) | 18 (0.2) | 104 (0.1) |
| Pacific Islander or Hawaiian Native | 0 (0) | 0 (0) | 4 (<0.1) |
| Multiple/Other | 2 (0.1) | 5 (<0.1) | 73 (<0.1) |
| Decline/Refuse | 11 (0.6) | 70 (0.6) | 651 (0.8) |
| Race Unknown | 0 (0) | 0 (0) | 0 (0) |
| Charlson Comorbidity Score, Median (IQR) | 3 (1 – 4) | 3 (1 – 4) | 2 (0 – 3) |
| Unknown | 189 | 1,592 | 19,145 |
| Inpatient Outcomes |  |  |  |
| Inpatient Length of Stay (Days), Median (IQR) | 7.6 (4.9 – 12.9) | 5.6 (3.2 – 9.8) | 3.8 (2.3 – 6.9) |
| SIRS Score (within 24-hours; max=4), Median (IQR) | 2 (1 – 2) | 1 (1 – 2) | 1 (0 – 1) |
| Unknown | 16 | 236 | 17,055 |
| In-Hospital Mortality Status, n (%) |  |  |  |
| In-Hospital Deceased | 100 (5.9) | 457 (3.9) | 671 (0.8) |
| Discharged Living | 1,603 (94) | 11,338 (96) | 85,001 (99) |
| Mortality Status (30-day), n (%) |  |  |  |
| Deceased (30-day) | 85 (5.0) | 392 (3.3) | 584 (0.7) |
| Living (30-day) | 1,618 (95) | 11,403 (97) | 85,088 (99) |
| ICU Status (anytime), n (%) |  |  |  |
| ICU Admission | 654 (38) | 3,626 (31) | 17,516 (20) |
| No ICU Admission | 1,049 (62) | 8,169 (69) | 68,156 (80) |
| Mechanical Ventilation (anytime), n (%) |  |  |  |
| Required Ventilation | 245 (14) | 1,450 (12) | 6,074 (7.1) |
| No Ventilation | 1,458 (86) | 10,345 (88) | 79,598 (93) |
| Inpatient Therapeutics |  |  |  |
| Antimicrobials (within 48-hours), n (%) |  |  |  |
| Received Antimicrobial (within 48-hrs) | 1,649 (97) | 11,446 (97) | 28,594 (33) |
| No Antimicrobial (within 48-hrs) | 54 (3.2) | 349 (3.0) | 57,078 (67) |
| Dexamethasone (within 48-hours), n (%) |  |  |  |
| Received Dexamethasone (within 48-hrs) | 32 (1.9) | 357 (3.0) | 3,593 (4.2) |
| No Dexamethasone (within 48-hrs) | 1,671 (98) | 11,438 (97) | 82,079 (96) |
| Pre-admission Comorbidities |  |  |  |
| Diabetic, n (%) | 651 (43) | 4,212 (41) | 20,685 (31) |
| Heart Failure or MI, n (%) | 505 (33) | 3,299 (32) | 17,493 (26) |
| Chronic pulmonary disease, n (%) | 399 (26) | 3,316 (33) | 17,495 (26) |
| Renal disease, n (%) | 559 (37) | 3,354 (33) | 14,682 (22) |
| Liver disease, n (%) | 320 (21) | 1,944 (19) | 8,408 (13) |
| Vascular disease, n (%) | 443 (29) | 2,711 (27) | 15,389 (23) |
| Cancer (any malignancy), n (%) | 387 (26) | 2,443 (24) | 13,220 (20) |
| Peptic ulcer disease, n (%) | 108 (7.1) | 663 (6.5) | 3,058 (4.6) |
| Hemiplegia or paraplegia, n (%) | 152 (10) | 883 (8.7) | 3,330 (5.0) |
| Rheumatoid disease, n (%) | 104 (6.9) | 744 (7.3) | 3,563 (5.4) |
| Dementia, n (%) | 57 (3.8) | 292 (2.9) | 1,207 (1.8) |
| AIDS/HIV, n (%) | 39 (2.6) | 317 (3.1) | 1,142 (1.7) |

**Sensitivity Testing**

**Section 1 (Reference Group Testing):** eTables testing pre-admission COVID-19 co-infection risk factor models and primary outcome (in-hospital mortality, mechanical ventilation, and ICU admission) models using the blood culture(-) suspected co-infection as the reference group.

- eTables 15-24
- eFigure 10

**Section 2 (Data Imputation Testing):** eTables testing in-hospital mortality outcome models with imputation procedures for missing data.

- eTables 25-27

**Section 3 (Combined Reference Group and Data Imputation Testing):** eTables testing in-hospital mortality outcome models using blood culture(-) suspected co-infection as the reference group and imputation procedures for missing data.

- eTables 28-30

**Section 4 (Pre-existing Conditions/Comorbidity History Testing):** eTables testing in-hospital mortality outcome models with solid organ transplant and hematologic disorder comorbidities as model variables.

- eTables 31-34

­­

**Sensitivity Testing Section 1:**

**(Reference Group Testing)**

| **eTable 15: 24-Hour Post-Admission Risk Factors of COVID-19 Bacterial Co-infection (Sensitivity Testing with Blood Culture(-) Suspected Co-infection and Blood Culture(+) Confirmed Co-infection groups only)** | | | | |
| --- | --- | --- | --- | --- |
|  | Total Encounters N | Co-infections,  N (% of Total) | COVID-19+ Co-infection, Adjusted OR (95% CI)^1,2^ | p-value^3^ |
| *UAB Cohort (n=3,786)* |  |  |  |  |
| SIRS Heart Rate (beats/min) |  |  |  | 0.052 |
| Heart Rate ≤90 | 1,587 | 30 (1.9) | — |  |
| Heart Rate >90 | 2,199 | 74 (3.4) | 1.52 (1.02 - 2.40) |  |
| SIRS Respiratory Rate (breaths/min) |  |  |  | 0.66 |
| Respiratory Rate ≤20 | 2,592 | 62 (2.4) | — |  |
| Respiratory Rate >20 | 1,194 | 42 (3.5) | 1.10 (0.70 - 1.68) |  |
| SIRS Temperature (C) |  |  |  | <0.001 |
| Temperature 36-38 C | 3,154 | 71 (2.3) | — |  |
| Temperature <36 or >38 C | 632 | 33 (5.2) | 2.14 (1.37 - 3.22) |  |
| SIRS White Blood Cell Count (10^3^/uL) |  |  |  | <0.001 |
| White Blood Cell Count 4-12 | 2,795 | 47 (1.7) | — |  |
| White Blood Cell Count <4 or >12 | 991 | 57 (5.8) | 3.01 (1.94 - 4.48) |  |
| Neutrophil to Lymphocyte Ratio |  |  |  | 0.004 |
| <15 | 3,281 | 72 (2.2) | — |  |
| ≥15 | 505 | 32 (6.3) | 1.95 (1.21 - 3.07) |  |
| *OLHS Cohort (n=5,141)* |  |  |  |  |
| SIRS Heart Rate (beats/min) |  |  |  | 0.007 |
| Heart Rate ≤90 | 1,218 | 30 (2.5) | — |  |
| Heart Rate >90 | 3,923 | 190 (4.8) | 1.72 (1.21 - 2.68) |  |
| SIRS Respiratory Rate (breaths/min) |  |  |  | 0.13 |
| Respiratory Rate ≤20 | 1,183 | 52 (4.4) | — |  |
| Respiratory Rate >20 | 3,958 | 168 (4.2) | 0.79 (0.56 - 1.11) |  |
| SIRS Temperature (C) |  |  |  | 0.12 |
| Temperature 36-38 C | 3,072 | 118 (3.8) | — |  |
| Temperature <36 or >38 C | 2,069 | 102 (4.9) | 1.24 (0.93 - 1.63) |  |
| SIRS White Blood Cell Count (10^3^/uL) |  |  |  | <0.001 |
| White Blood Cell Count 4-12 | 3,580 | 87 (2.4) | — |  |
| White Blood Cell Count <4 or >12 | 1,561 | 133 (8.5) | 2.92 (2.20 - 4.03) |  |
| Neutrophil to Lymphocyte Ratio |  |  |  | <0.001 |
| <15 | 4,500 | 144 (3.2) | — |  |
| ≥15 | 641 | 76 (12) | 2.89 (2.09 - 3.91) |  |
| ^1^Adjusted Odds Ratio 95% confidence interval (n=1000 bootstraps) | | | | |
| ^2^OR = Odds Ratio, CI = Confidence Interval | | | | |
| ^3^Wald Test | | | | |

| **eTable 16: UAB Cohort In-Hospital Mortality Models (Sensitivity Testing with Blood Culture(-) Suspected Co-infection as Reference)** | | | | | | |
| --- | --- | --- | --- | --- | --- | --- |
| Characteristic | Encounters Total N | Deaths, N (% of Total) | In-Hospital Mortality Unadjusted OR (95% CI)^1,2^ | p-value^3^ | In-Hospital Mortality Adjusted OR (95% CI)^2,4^ | p-value^3^ |
| Age Range |  |  |  | <0.001 |  | <0.001 |
| <65 years | 547 | 76 (14) | — |  | — |  |
| ≥65 years | 429 | 121 (28) | 2.40 (1.81 - 3.41) |  | 2.44 (1.74 - 3.40) |  |
| Diabetes History |  |  |  | 0.056 |  | 0.62 |
| No Diabetic History | 525 | 94 (18) | — |  | — |  |
| Diabetic History | 451 | 103 (23) | 1.36 (1.01 - 1.91) |  | 1.09 (0.75 - 1.57) |  |
| COPD History |  |  |  | 0.48 |  | 0.63 |
| No COPD History | 689 | 135 (20) | — |  | — |  |
| COPD History | 287 | 62 (22) | 1.12 (0.80 - 1.60) |  | 0.91 (0.60 - 1.34) |  |
| Heart Failure or MI History |  |  |  | <0.001 |  | 0.023 |
| No Cardiac History | 648 | 107 (17) | — |  | — |  |
| Heart Failure or MI History | 328 | 90 (27) | 1.91 (1.39 - 2.65) |  | 1.52 (1.08 - 2.27) |  |
| Renal Disease History |  |  |  | 0.005 |  | 0.18 |
| No Renal History | 639 | 112 (18) | — |  | — |  |
| Renal Disease History | 337 | 85 (25) | 1.59 (1.14 - 2.20) |  | 1.28 (0.88 - 1.85) |  |
| Sex |  |  |  | 0.040 |  | 0.10 |
| Female | 475 | 83 (17) | — |  | — |  |
| Male | 501 | 114 (23) | 1.38 (1.01 - 1.93) |  | 1.32 (0.94 - 1.85) |  |
| First 24-hour SIRS Score |  |  |  | 0.040 |  | 0.004 |
| <2 SIRS Score | 430 | 74 (17) | — |  | — |  |
| ≥2 SIRS Score | 546 | 123 (23) | 1.40 (1.00 - 1.92) |  | 1.64 (1.18 - 2.33) |  |
| COVID-19+ Co-infection Status |  |  |  | 0.081 |  | 0.49 |
| Blood Culture(-) Suspected Co-infection | 884 | 172 (19) | — |  | — |  |
| Blood Culture(+) Confirmed Co-infection | 92 | 25 (27) | 1.53 (0.91 - 2.49) |  | 1.23 (0.67 - 1.98) |  |
| ^1^Unadjusted Odds Ratio 95% confidence interval (n=1000 bootstraps) | | | | | | |
| ^2^OR = Odds Ratio, CI = Confidence Interval | | | | | | |
| ^3^Wald Test | | | | | | |
| ^4^Adjusted Odds Ratio 95% confidence interval (n=1000 bootstraps) | | | | | | |

| **eTable 17: OLHS Cohort In-Hospital Mortality Models (Sensitivity Testing with Blood Culture(-) Suspected Co-infection as Reference)** | | | | | | |
| --- | --- | --- | --- | --- | --- | --- |
| Characteristic | Encounters Total N | Deaths, N (% of Total) | In-Hospital Mortality Unadjusted OR (95% CI)^1,2^ | p-value^3^ | In-Hospital Mortality Adjusted OR (95% CI)^2,4^ | p-value^3^ |
| Age Range |  |  |  | <0.001 |  | <0.001 |
| <65 years | 2,169 | 197 (9.1) | — |  | — |  |
| ≥65 years | 2,517 | 449 (18) | 2.17 (1.81 - 2.59) |  | 2.22 (1.86 - 2.70) |  |
| Diabetes History |  |  |  | 0.074 |  | 0.93 |
| No Diabetic History | 2,898 | 379 (13) | — |  | — |  |
| Diabetic History | 1,788 | 267 (15) | 1.16 (0.99 - 1.38) |  | 1.01 (0.85 - 1.21) |  |
| COPD History |  |  |  | 0.012 |  | 0.68 |
| No COPD History | 3,510 | 458 (13) | — |  | — |  |
| COPD History | 1,176 | 188 (16) | 1.27 (1.04 - 1.51) |  | 1.04 (0.86 - 1.26) |  |
| Heart Failure or MI History |  |  |  | <0.001 |  | <0.001 |
| No Cardiac History | 3,576 | 432 (12) | — |  | — |  |
| Heart Failure or MI History | 1,110 | 214 (19) | 1.74 (1.45 - 2.07) |  | 1.59 (1.29 - 1.95) |  |
| Renal Disease History |  |  |  | 0.009 |  | 0.55 |
| No Renal History | 3,461 | 450 (13) | — |  | — |  |
| Renal Disease History | 1,225 | 196 (16) | 1.27 (1.07 - 1.52) |  | 0.93 (0.76 - 1.15) |  |
| Sex |  |  |  | <0.001 |  | <0.001 |
| Female | 2,309 | 274 (12) | — |  | — |  |
| Male | 2,377 | 372 (16) | 1.38 (1.17 - 1.64) |  | 1.42 (1.19 - 1.70) |  |
| First 24-hour SIRS Score |  |  |  | <0.001 |  | <0.001 |
| <2 SIRS Score | 1,054 | 81 (7.7) | — |  | — |  |
| ≥2 SIRS Score | 3,632 | 565 (16) | 2.21 (1.74 - 2.89) |  | 2.49 (1.99 - 3.30) |  |
| COVID-19+ Co-infection Status |  |  |  | 0.003 |  | 0.010 |
| Blood Culture(-) Suspected Co-infection | 4,475 | 602 (13) | — |  | — |  |
| Blood Culture(+) Confirmed Co-infection | 211 | 44 (21) | 1.69 (1.20 - 2.34) |  | 1.61 (1.10 - 2.21) |  |
| ^1^Unadjusted Odds Ratio 95% confidence interval (n=1000 bootstraps) | | | | | | |
| ^2^OR = Odds Ratio, CI = Confidence Interval | | | | | | |
| ^3^Wald Test | | | | | | |
| ^4^Adjusted Odds Ratio 95% confidence interval (n=1000 bootstraps) | | | | | | |

| **eTable 18: Combined UAB and OLHS Cohorts In-Hospital Mortality Models (Sensitivity Testing with Blood Culture(-) Suspected Co-infection as Reference)** | | | | | | |
| --- | --- | --- | --- | --- | --- | --- |
| Characteristic | Encounters Total N | Deaths, N (% of Total) | In-Hospital Mortality Unadjusted OR (95% CI)^1,2^ | p-value^3^ | In-Hospital Mortality Adjusted OR (95% CI)^2,4^ | p-value^3^ |
| Age Range |  |  |  | <0.001 |  | <0.001 |
| <65 years | 2,716 | 273 (10) | — |  | — |  |
| ≥65 years | 2,946 | 570 (19) | 2.14 (1.83 - 2.51) |  | 2.11 (1.82 - 2.48) |  |
| Diabetes History |  |  |  | 0.005 |  | 0.58 |
| No Diabetic History | 3,423 | 473 (14) | — |  | — |  |
| Diabetic History | 2,239 | 370 (17) | 1.24 (1.08 - 1.43) |  | 1.05 (0.89 - 1.22) |  |
| COPD History |  |  |  | 0.006 |  | 0.80 |
| No COPD History | 4,199 | 593 (14) | — |  | — |  |
| COPD History | 1,463 | 250 (17) | 1.25 (1.07 - 1.47) |  | 1.02 (0.86 - 1.22) |  |
| Heart Failure or MI History |  |  |  | <0.001 |  | <0.001 |
| No Cardiac History | 4,224 | 539 (13) | — |  | — |  |
| Heart Failure or MI History | 1,438 | 304 (21) | 1.84 (1.56 - 2.13) |  | 1.62 (1.37 - 1.94) |  |
| Renal Disease History |  |  |  | <0.001 |  | 0.81 |
| No Renal History | 4,100 | 562 (14) | — |  | — |  |
| Renal Disease History | 1,562 | 281 (18) | 1.37 (1.16 - 1.62) |  | 1.02 (0.85 - 1.22) |  |
| Sex |  |  |  | <0.001 |  | <0.001 |
| Female | 2,784 | 357 (13) | — |  | — |  |
| Male | 2,878 | 486 (17) | 1.39 (1.20 - 1.61) |  | 1.41 (1.20 - 1.63) |  |
| First 24-hour SIRS Score |  |  |  | <0.001 |  | <0.001 |
| <2 SIRS Score | 1,484 | 155 (10) | — |  | — |  |
| ≥2 SIRS Score | 4,178 | 688 (16) | 1.70 (1.41 - 2.07) |  | 1.91 (1.58 - 2.34) |  |
| COVID-19+ Co-infection Status |  |  |  | <0.001 |  | 0.001 |
| Blood Culture(-) Suspected Co-infection | 5,359 | 774 (14) | — |  | — |  |
| Blood Culture(+) Confirmed Co-infection | 303 | 69 (23) | 1.76 (1.29 - 2.30) |  | 1.60 (1.17 - 2.10) |  |
| ^1^Unadjusted Odds Ratio 95% confidence interval (n=1000 bootstraps) | | | | | | |
| ^2^OR = Odds Ratio, CI = Confidence Interval | | | | | | |
| ^3^Wald Test | | | | | | |
| ^4^Adjusted Odds Ratio 95% confidence interval (n=1000 bootstraps) | | | | | | |

| **eTable 19: UAB Cohort Mechanical Ventilation Models (Sensitivity Testing with Blood Culture(-) Suspected Co-infection as Reference)** | | | | | | |
| --- | --- | --- | --- | --- | --- | --- |
| Characteristic | Encounters Total N | Required Ventilation N (% of Total) | Required Ventilation Unadjusted OR (95% CI)^1,2^ | p-value^3^ | Required Ventilation Adjusted OR (95% CI)^2,4^ | p-value^3^ |
| Age Range |  |  |  | 0.86 |  | 0.61 |
| <65 years | 547 | 140 (26) | — |  | — |  |
| ≥65 years | 429 | 112 (26) | 1.02 (0.78 - 1.32) |  | 1.08 (0.80 - 1.48) |  |
| Diabetes History |  |  |  | 0.12 |  | 0.11 |
| No Diabetic History | 525 | 125 (24) | — |  | — |  |
| Diabetic History | 451 | 127 (28) | 1.25 (0.94 - 1.67) |  | 1.30 (0.95 - 1.82) |  |
| COPD History |  |  |  | 0.11 |  | 0.071 |
| No COPD History | 689 | 188 (27) | — |  | — |  |
| COPD History | 287 | 64 (22) | 0.76 (0.53 - 1.09) |  | 0.73 (0.50 - 1.05) |  |
| Heart Failure or MI History |  |  |  | 0.61 |  | 0.94 |
| No Cardiac History | 648 | 164 (25) | — |  | — |  |
| Heart Failure or MI History | 328 | 88 (27) | 1.09 (0.80 - 1.43) |  | 1.02 (0.72 - 1.40) |  |
| Renal Disease History |  |  |  | 0.17 |  | 0.24 |
| No Renal History | 639 | 156 (24) | — |  | — |  |
| Renal Disease History | 337 | 96 (28) | 1.23 (0.90 - 1.66) |  | 1.22 (0.88 - 1.70) |  |
| Sex |  |  |  | 0.16 |  | 0.20 |
| Female | 475 | 113 (24) | — |  | — |  |
| Male | 501 | 139 (28) | 1.24 (0.93 - 1.68) |  | 1.22 (0.90 - 1.67) |  |
| First 24-hour SIRS Score |  |  |  | <0.001 |  | <0.001 |
| <2 SIRS Score | 430 | 88 (20) | — |  | — |  |
| ≥2 SIRS Score | 546 | 164 (30) | 1.69 (1.25 - 2.25) |  | 1.82 (1.34 - 2.53) |  |
| COVID-19+ Co-infection Status |  |  |  | 0.95 |  | 0.53 |
| Blood Culture(-) Suspected Co-infection | 884 | 228 (26) | — |  | — |  |
| Blood Culture(+) Confirmed Co-infection | 92 | 24 (26) | 1.01 (0.59 - 1.61) |  | 0.85 (0.49 - 1.36) |  |
| ^1^Unadjusted Odds Ratio 95% confidence interval (n=1000 bootstraps) | | | | | | |
| ^2^OR = Odds Ratio, CI = Confidence Interval | | | | | | |
| ^3^Wald Test | | | | | | |
| ^4^Adjusted Odds Ratio 95% confidence interval (n=1000 bootstraps) | | | | | | |

| **eTable 20: OLHS Cohort Mechanical Ventilation Models (Sensitivity Testing with Blood Culture(-) Suspected Co-infection as Reference)** | | | | | | |
| --- | --- | --- | --- | --- | --- | --- |
| Characteristic | Encounters Total N | Required Ventilation N (% of Total) | Required Ventilation Unadjusted OR (95% CI)^1,2^ | p-value^3^ | Required Ventilation Adjusted OR (95% CI)^2,4^ | p-value^3^ |
| Age Range |  |  |  | 0.053 |  | 0.004 |
| <65 years | 2,169 | 349 (16) | — |  | — |  |
| ≥65 years | 2,517 | 459 (18) | 1.16 (1.00 - 1.35) |  | 1.26 (1.09 - 1.47) |  |
| Diabetes History |  |  |  | 0.62 |  | 0.59 |
| No Diabetic History | 2,898 | 506 (17) | — |  | — |  |
| Diabetic History | 1,788 | 302 (17) | 0.96 (0.83 - 1.12) |  | 0.95 (0.81 - 1.12) |  |
| COPD History |  |  |  | 0.25 |  | 0.19 |
| No COPD History | 3,510 | 618 (18) | — |  | — |  |
| COPD History | 1,176 | 190 (16) | 0.90 (0.75 - 1.09) |  | 0.88 (0.73 - 1.06) |  |
| Heart Failure or MI History |  |  |  | 0.81 |  | 0.77 |
| No Cardiac History | 3,576 | 614 (17) | — |  | — |  |
| Heart Failure or MI History | 1,110 | 194 (17) | 1.02 (0.86 - 1.22) |  | 1.03 (0.84 - 1.27) |  |
| Renal Disease History |  |  |  | 0.55 |  | 0.48 |
| No Renal History | 3,461 | 590 (17) | — |  | — |  |
| Renal Disease History | 1,225 | 218 (18) | 1.05 (0.88 - 1.24) |  | 1.07 (0.87 - 1.30) |  |
| Sex |  |  |  | 0.004 |  | 0.008 |
| Female | 2,309 | 361 (16) | — |  | — |  |
| Male | 2,377 | 447 (19) | 1.25 (1.08 - 1.45) |  | 1.23 (1.05 - 1.44) |  |
| First 24-hour SIRS Score |  |  |  | <0.001 |  | <0.001 |
| <2 SIRS Score | 1,054 | 96 (9.1) | — |  | — |  |
| ≥2 SIRS Score | 3,632 | 712 (20) | 2.43 (1.94 - 3.09) |  | 2.50 (2.01 - 3.18) |  |
| COVID-19+ Co-infection Status |  |  |  | 0.16 |  | 0.32 |
| Blood Culture(-) Suspected Co-infection | 4,475 | 764 (17) | — |  | — |  |
| Blood Culture(+) Confirmed Co-infection | 211 | 44 (21) | 1.29 (0.87 - 1.77) |  | 1.19 (0.82 - 1.67) |  |
| ^1^Unadjusted Odds Ratio 95% confidence interval (n=1000 bootstraps) | | | | | | |
| ^2^OR = Odds Ratio, CI = Confidence Interval | | | | | | |
| ^3^Wald Test | | | | | | |
| ^4^Adjusted Odds Ratio 95% confidence interval (n=1000 bootstraps) | | | | | | |

| **eTable 21: Combined UAB and OLHS Cohorts Mechanical Ventilation Models (Sensitivity Testing with Blood Culture(-) Suspected Co-infection as Reference)** | | | | | | |
| --- | --- | --- | --- | --- | --- | --- |
| Characteristic | Encounters Total N | Required Ventilation N (% of Total) | Required Ventilation Unadjusted OR (95% CI)^1,2^ | p-value^3^ | Required Ventilation Adjusted OR (95% CI)^2,4^ | p-value^3^ |
| Age Range |  |  |  | 0.18 |  | 0.067 |
| <65 years | 2,716 | 489 (18) | — |  | — |  |
| ≥65 years | 2,946 | 571 (19) | 1.09 (0.95 - 1.24) |  | 1.14 (1.00 - 1.31) |  |
| Diabetes History |  |  |  | 0.49 |  | 0.62 |
| No Diabetic History | 3,423 | 631 (18) | — |  | — |  |
| Diabetic History | 2,239 | 429 (19) | 1.04 (0.91 - 1.19) |  | 1.04 (0.89 - 1.20) |  |
| COPD History |  |  |  | 0.12 |  | 0.068 |
| No COPD History | 4,199 | 806 (19) | — |  | — |  |
| COPD History | 1,463 | 254 (17) | 0.89 (0.76 - 1.03) |  | 0.86 (0.72 - 1.00) |  |
| Heart Failure or MI History |  |  |  | 0.32 |  | 0.46 |
| No Cardiac History | 4,224 | 778 (18) | — |  | — |  |
| Heart Failure or MI History | 1,438 | 282 (20) | 1.08 (0.93 - 1.24) |  | 1.07 (0.89 - 1.27) |  |
| Renal Disease History |  |  |  | 0.10 |  | 0.13 |
| No Renal History | 4,100 | 746 (18) | — |  | — |  |
| Renal Disease History | 1,562 | 314 (20) | 1.13 (0.96 - 1.30) |  | 1.13 (0.96 - 1.36) |  |
| Sex |  |  |  | 0.001 |  | 0.002 |
| Female | 2,784 | 474 (17) | — |  | — |  |
| Male | 2,878 | 586 (20) | 1.24 (1.09 - 1.41) |  | 1.23 (1.08 - 1.41) |  |
| First 24-hour SIRS Score |  |  |  | <0.001 |  | <0.001 |
| <2 SIRS Score | 1,484 | 184 (12) | — |  | — |  |
| ≥2 SIRS Score | 4,178 | 876 (21) | 1.88 (1.60 - 2.24) |  | 1.92 (1.62 - 2.28) |  |
| COVID-19+ Co-infection Status |  |  |  | 0.089 |  | 0.25 |
| Blood Culture(-) Suspected Co-infection | 5,359 | 992 (19) | — |  | — |  |
| Blood Culture(+) Confirmed Co-infection | 303 | 68 (22) | 1.26 (0.93 - 1.66) |  | 1.17 (0.88 - 1.56) |  |
| ^1^Unadjusted Odds Ratio 95% confidence interval (n=1000 bootstraps) | | | | | | |
| ^2^OR = Odds Ratio, CI = Confidence Interval | | | | | | |
| ^3^Wald Test | | | | | | |
| ^4^Adjusted Odds Ratio 95% confidence interval (n=1000 bootstraps) | | | | | | |

| **eTable 22: UAB Cohort ICU Admission Models (Sensitivity Testing with Blood Culture(-) Suspected Co-infection as Reference)** | | | | | | |
| --- | --- | --- | --- | --- | --- | --- |
| Characteristic | Encounters Total N | ICU Admissions N (% of Total) | ICU Admission Unadjusted OR (95% CI)^1,2^ | p-value^3^ | ICU Admission Adjusted OR (95% CI)^2,4^ | p-value^3^ |
| Age Range |  |  |  | 0.31 |  | 0.23 |
| <65 years | 547 | 251 (46) | — |  | — |  |
| ≥65 years | 429 | 211 (49) | 1.13 (0.89 - 1.46) |  | 1.17 (0.92 - 1.56) |  |
| Diabetes History |  |  |  | 0.11 |  | 0.18 |
| No Diabetic History | 525 | 236 (45) | — |  | — |  |
| Diabetic History | 451 | 226 (50) | 1.23 (0.96 - 1.59) |  | 1.21 (0.93 - 1.60) |  |
| COPD History |  |  |  | 0.41 |  | 0.17 |
| No COPD History | 689 | 332 (48) | — |  | — |  |
| COPD History | 287 | 130 (45) | 0.89 (0.67 - 1.17) |  | 0.80 (0.61 - 1.12) |  |
| Heart Failure or MI History |  |  |  | 0.016 |  | 0.068 |
| No Cardiac History | 648 | 289 (45) | — |  | — |  |
| Heart Failure or MI History | 328 | 173 (53) | 1.40 (1.08 - 1.82) |  | 1.33 (0.98 - 1.81) |  |
| Renal Disease History |  |  |  | 0.12 |  | 0.50 |
| No Renal History | 639 | 291 (46) | — |  | — |  |
| Renal Disease History | 337 | 171 (51) | 1.23 (0.95 - 1.59) |  | 1.11 (0.82 - 1.51) |  |
| Sex |  |  |  | 0.031 |  | 0.051 |
| Female | 475 | 208 (44) | — |  | — |  |
| Male | 501 | 254 (51) | 1.32 (1.03 - 1.71) |  | 1.29 (0.99 - 1.67) |  |
| First 24-hour SIRS Score |  |  |  | <0.001 |  | <0.001 |
| <2 SIRS Score | 430 | 167 (39) | — |  | — |  |
| ≥2 SIRS Score | 546 | 295 (54) | 1.84 (1.43 - 2.41) |  | 2.00 (1.55 - 2.61) |  |
| COVID-19+ Co-infection Status |  |  |  | 0.23 |  | 0.81 |
| Blood Culture(-) Suspected Co-infection | 884 | 413 (47) | — |  | — |  |
| Blood Culture(+) Confirmed Co-infection | 92 | 49 (53) | 1.28 (0.83 - 1.97) |  | 1.07 (0.69 - 1.71) |  |
| ^1^Unadjusted Odds Ratio 95% confidence interval (n=1000 bootstraps) | | | | | | |
| ^2^OR = Odds Ratio, CI = Confidence Interval | | | | | | |
| ^3^Wald Test | | | | | | |
| ^4^Adjusted Odds Ratio 95% confidence interval (n=1000 bootstraps) | | | | | | |

| **eTable 23: OLHS Cohort ICU Admission Models (Sensitivity Testing with Blood Culture(-) Suspected Co-infection as Reference)** | | | | | | |
| --- | --- | --- | --- | --- | --- | --- |
| Characteristic | Encounters Total N | ICU Admissions N (% of Total) | ICU Admission Unadjusted OR (95% CI)^1,2^ | p-value^3^ | ICU Admission Adjusted OR (95% CI)^2,4^ | p-value^3^ |
| Age Range |  |  |  | 0.31 |  | 0.12 |
| <65 years | 2,169 | 933 (43) | — |  | — |  |
| ≥65 years | 2,517 | 1120 (44) | 1.06 (0.95 - 1.19) |  | 1.10 (0.98 - 1.25) |  |
| Diabetes History |  |  |  | 0.19 |  | 0.31 |
| No Diabetic History | 2,898 | 1248 (43) | — |  | — |  |
| Diabetic History | 1,788 | 805 (45) | 1.08 (0.96 - 1.20) |  | 1.08 (0.94 - 1.22) |  |
| COPD History |  |  |  | 0.93 |  | 0.60 |
| No COPD History | 3,510 | 1539 (44) | — |  | — |  |
| COPD History | 1,176 | 514 (44) | 1.00 (0.87 - 1.14) |  | 0.96 (0.83 - 1.10) |  |
| Heart Failure or MI History |  |  |  | 0.087 |  | 0.15 |
| No Cardiac History | 3,576 | 1542 (43) | — |  | — |  |
| Heart Failure or MI History | 1,110 | 511 (46) | 1.12 (0.98 - 1.29) |  | 1.12 (0.95 - 1.30) |  |
| Renal Disease History |  |  |  | 0.34 |  | 0.92 |
| No Renal History | 3,461 | 1502 (43) | — |  | — |  |
| Renal Disease History | 1,225 | 551 (45) | 1.07 (0.94 - 1.23) |  | 1.00 (0.87 - 1.16) |  |
| Sex |  |  |  | 0.004 |  | 0.005 |
| Female | 2,309 | 963 (42) | — |  | — |  |
| Male | 2,377 | 1090 (46) | 1.19 (1.05 - 1.33) |  | 1.18 (1.06 - 1.33) |  |
| First 24-hour SIRS Score |  |  |  | <0.001 |  | <0.001 |
| <2 SIRS Score | 1,054 | 357 (34) | — |  | — |  |
| ≥2 SIRS Score | 3,632 | 1696 (47) | 1.71 (1.48 - 1.99) |  | 1.73 (1.50 - 1.99) |  |
| COVID-19+ Co-infection Status |  |  |  | <0.001 |  | 0.002 |
| Blood Culture(-) Suspected Co-infection | 4,475 | 1935 (43) | — |  | — |  |
| Blood Culture(+) Confirmed Co-infection | 211 | 118 (56) | 1.66 (1.27 - 2.19) |  | 1.58 (1.18 - 2.13) |  |
| ^1^Unadjusted Odds Ratio 95% confidence interval (n=1000 bootstraps) | | | | | | |
| ^2^OR = Odds Ratio, CI = Confidence Interval | | | | | | |
| ^3^Wald Test | | | | | | |
| ^4^Adjusted Odds Ratio 95% confidence interval (n=1000 bootstraps) | | | | | | |

| **eTable 24: Combined UAB and OLHS Cohorts ICU Admission Models (Sensitivity Testing with Blood Culture(-) Suspected Co-infection as Reference)** | | | | | | |
| --- | --- | --- | --- | --- | --- | --- |
| Characteristic | Encounters Total N | ICU Admissions N (% of Total) | ICU Admission Unadjusted OR (95% CI)^1,2^ | p-value^3^ | ICU Admission Adjusted OR (95% CI)^2,4^ | p-value^3^ |
| Age Range |  |  |  | 0.23 |  | 0.14 |
| <65 years | 2,716 | 1184 (44) | — |  | — |  |
| ≥65 years | 2,946 | 1331 (45) | 1.06 (0.96 - 1.18) |  | 1.08 (0.98 - 1.22) |  |
| Diabetes History |  |  |  | 0.046 |  | 0.12 |
| No Diabetic History | 3,423 | 1484 (43) | — |  | — |  |
| Diabetic History | 2,239 | 1031 (46) | 1.11 (1.01 - 1.24) |  | 1.10 (0.97 - 1.23) |  |
| COPD History |  |  |  | 0.72 |  | 0.33 |
| No COPD History | 4,199 | 1871 (45) | — |  | — |  |
| COPD History | 1,463 | 644 (44) | 0.98 (0.87 - 1.09) |  | 0.94 (0.83 - 1.07) |  |
| Heart Failure or MI History |  |  |  | 0.005 |  | 0.021 |
| No Cardiac History | 4,224 | 1831 (43) | — |  | — |  |
| Heart Failure or MI History | 1,438 | 684 (48) | 1.18 (1.05 - 1.33) |  | 1.18 (1.03 - 1.35) |  |
| Renal Disease History |  |  |  | 0.092 |  | 0.62 |
| No Renal History | 4,100 | 1793 (44) | — |  | — |  |
| Renal Disease History | 1,562 | 722 (46) | 1.11 (0.98 - 1.25) |  | 1.03 (0.90 - 1.18) |  |
| Sex |  |  |  | <0.001 |  | <0.001 |
| Female | 2,784 | 1171 (42) | — |  | — |  |
| Male | 2,878 | 1344 (47) | 1.21 (1.08 - 1.35) |  | 1.20 (1.08 - 1.33) |  |
| First 24-hour SIRS Score |  |  |  | <0.001 |  | <0.001 |
| <2 SIRS Score | 1,484 | 524 (35) | — |  | — |  |
| ≥2 SIRS Score | 4,178 | 1991 (48) | 1.67 (1.47 - 1.90) |  | 1.71 (1.51 - 1.95) |  |
| COVID-19+ Co-infection Status |  |  |  | <0.001 |  | 0.002 |
| Blood Culture(-) Suspected Co-infection | 5,359 | 2348 (44) | — |  | — |  |
| Blood Culture(+) Confirmed Co-infection | 303 | 167 (55) | 1.58 (1.24 - 2.02) |  | 1.47 (1.16 - 1.83) |  |
| ^1^Unadjusted Odds Ratio 95% confidence interval (n=1000 bootstraps) | | | | | | |
| ^2^OR = Odds Ratio, CI = Confidence Interval | | | | | | |
| ^3^Wald Test | | | | | | |
| ^4^Adjusted Odds Ratio 95% confidence interval (n=1000 bootstraps) | | | | | | |


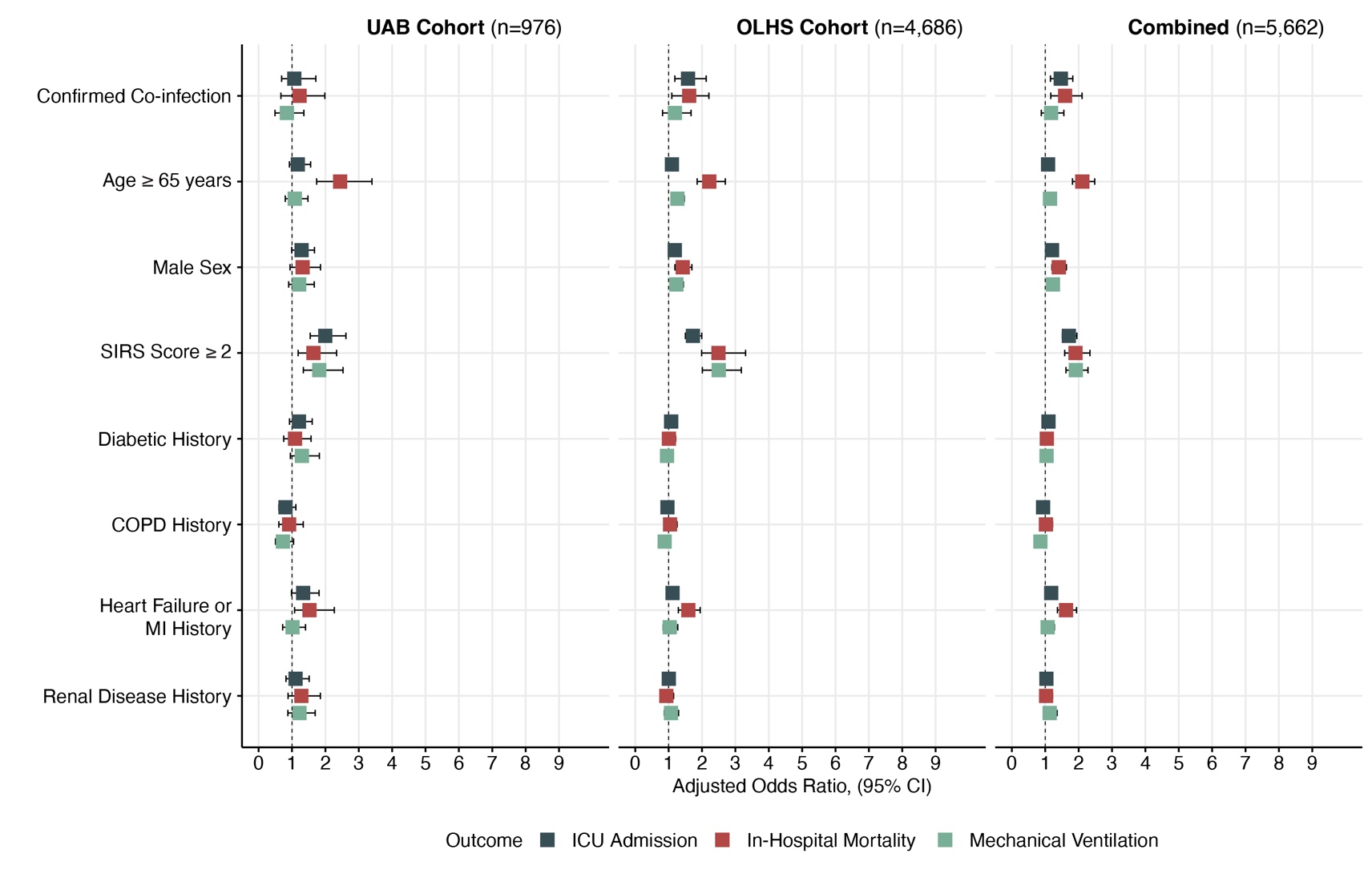


**eFigure 10: In-Hospital Mortality, Mechanical Ventilation, and ICU Admission Multi-variate Logistic Regression Outcome Models (Sensitivity Testing with Blood Culture(-) Suspected Co-infection as Reference).** Multivariable logistic regression models show risk factors that increase likelihood of in-hospital mortality (red), mechanical ventilation (green), and ICU admission (dark grey). Model variables included culture status within 48-hours of admission (reference: 48-hour blood culture negative suspected infection), age ≥65 (reference*: age <65), male sex (reference*: female sex), 24-hour post-admission SIRS score ≥2 (reference*: 24-hour post-admission SIRS score <2), diabetic history, COPD history, heart failure or MI history, renal disease history (reference*: no pre-admission history of respective comorbidity). For combined model details see supplemental eTables 16-24. Abbreviations: reference*, reference not shown; SIRS, severe inflammatory response syndrome score; MI, myocardial infarction; COPD, chronic obstructive pulmonary disease; UAB, University of Alabama at Birmingham cohort; OLHS, Ochsner Louisiana State University Health – Shreveport cohort.

**Sensitivity Testing Section 2:**

**(Data Imputation)**

| **eTable 25: OLHS Cohort In-Hospital Mortality Models (Sensitivity Testing with Imputed Data)** | | | | | | |
| --- | --- | --- | --- | --- | --- | --- |
| Characteristic | Encounters Total N | Deaths, N (% of Total) | In-Hospital Mortality Unadjusted OR (95% CI)^1,2^ | p-value^3^ | In-Hospital Mortality Adjusted OR (95% CI)^2,4^ | p-value^3^ |
| Age Range |  |  |  | <0.001 |  | <0.001 |
| <65 years | 5,198 | 284 (5.5) | — |  | — |  |
| ≥65 years | 4,508 | 623 (14) | 2.77 (2.43 - 3.21) |  | 2.50 (2.14 - 2.94) |  |
| Diabetes History |  |  |  | <0.001 |  | 0.38 |
| No Diabetic History | 6,546 | 540 (8.2) | — |  | — |  |
| Diabetic History | 3,160 | 367 (12) | 1.46 (1.26 - 1.67) |  | 1.07 (0.92 - 1.27) |  |
| COPD History |  |  |  | 0.002 |  | 0.48 |
| No COPD History | 7,433 | 656 (8.8) | — |  | — |  |
| COPD History | 2,273 | 251 (11) | 1.28 (1.10 - 1.50) |  | 0.95 (0.81 - 1.10) |  |
| Heart Failure or MI History |  |  |  | <0.001 |  | <0.001 |
| No Cardiac History | 7,651 | 617 (8.1) | — |  | — |  |
| Heart Failure or MI History | 2,055 | 290 (14) | 1.87 (1.61 - 2.14) |  | 1.54 (1.27 - 1.82) |  |
| Renal Disease History |  |  |  | <0.001 |  | 0.52 |
| No Renal History | 7,630 | 641 (8.4) | — |  | — |  |
| Renal Disease History | 2,076 | 266 (13) | 1.60 (1.37 - 1.87) |  | 1.06 (0.90 - 1.26) |  |
| Sex |  |  |  | <0.001 |  | <0.001 |
| Female | 4,893 | 385 (7.9) | — |  | — |  |
| Male | 4,813 | 522 (11) | 1.43 (1.24 - 1.64) |  | 1.38 (1.19 - 1.61) |  |
| First 24-hour SIRS Score |  |  |  | <0.001 |  | <0.001 |
| <2 SIRS Score | 2,770 | 134 (4.8) | — |  | — |  |
| ≥2 SIRS Score | 6,936 | 773 (11) | 2.47 (2.04 - 3.04) |  | 2.35 (1.96 - 2.91) |  |
| COVID-19+ Co-infection Status |  |  |  | <0.001 |  | <0.001 |
| No Blood Culture, No Blood Culture | 4,401 | 223 (5.1) | — |  | — |  |
| Blood Culture(-) Suspected Co-infection | 5,065 | 632 (12) | 2.67 (2.29 - 3.16) |  | 2.19 (1.87 - 2.60) |  |
| Blood Culture(+) Confirmed Co-infection | 240 | 52 (22) | 5.19 (3.61 - 7.14) |  | 3.85 (2.66 - 5.37) |  |
| ^1^Unadjusted Odds Ratio 95% confidence interval (n=1000 bootstraps, median imputation and binarization for missing 24-hour post-admission SIRS score, k-nearest neighbors imputation for missing comorbidity history) | | | | | | |
| ^2^OR = Odds Ratio, CI = Confidence Interval | | | | | | |
| ^3^Wald Test | | | | | | |
| ^4^Adjusted Odds Ratio 95% confidence interval (n=1000 bootstraps, median imputation and binarization for missing 24-hour post-admission SIRS score, k-nearest neighbors imputation for missing comorbidity history) | | | | | | |

| **eTable 26: UAB Cohort In-Hospital Mortality Models (Sensitivity Testing with Imputed Data)** | | | | | | |
| --- | --- | --- | --- | --- | --- | --- |
| Characteristic | Encounters Total N | Deaths, N (% of Total) | In-Hospital Mortality Unadjusted OR (95% CI)^1,2^ | p-value^3^ | In-Hospital Mortality Adjusted OR (95% CI)^2,4^ | p-value^3^ |
| Age Range |  |  |  | <0.001 |  | <0.001 |
| <65 years | 2,457 | 199 (8.1) | — |  | — |  |
| ≥65 years | 1,618 | 289 (18) | 2.48 (2.06 - 3.01) |  | 2.36 (1.94 - 2.88) |  |
| Diabetes History |  |  |  | 0.039 |  | 0.17 |
| No Diabetic History | 2,232 | 246 (11) | — |  | — |  |
| Diabetic History | 1,843 | 242 (13) | 1.22 (1.00 - 1.46) |  | 0.86 (0.70 - 1.06) |  |
| COPD History |  |  |  | 0.049 |  | 0.70 |
| No COPD History | 3,157 | 361 (11) | — |  | — |  |
| COPD History | 918 | 127 (14) | 1.25 (0.99 - 1.55) |  | 0.95 (0.73 - 1.22) |  |
| Heart Failure or MI History |  |  |  | <0.001 |  | <0.001 |
| No Cardiac History | 3,082 | 291 (9.4) | — |  | — |  |
| Heart Failure or MI History | 993 | 197 (20) | 2.38 (1.95 - 2.89) |  | 1.82 (1.44 - 2.32) |  |
| Renal Disease History |  |  |  | <0.001 |  | 0.007 |
| No Renal History | 3,159 | 313 (9.9) | — |  | — |  |
| Renal Disease History | 916 | 175 (19) | 2.13 (1.77 - 2.59) |  | 1.40 (1.08 - 1.80) |  |
| Sex |  |  |  | <0.001 |  | <0.001 |
| Female | 2,070 | 196 (9.5) | — |  | — |  |
| Male | 2,005 | 292 (15) | 1.64 (1.34 - 2.00) |  | 1.47 (1.21 - 1.80) |  |
| First 24-hour SIRS Score |  |  |  | <0.001 |  | <0.001 |
| <2 SIRS Score | 2,525 | 232 (9.2) | — |  | — |  |
| ≥2 SIRS Score | 1,550 | 256 (17) | 1.97 (1.62 - 2.36) |  | 1.52 (1.22 - 1.89) |  |
| COVID-19+ Co-infection Status |  |  |  | <0.001 |  | <0.001 |
| No Blood Culture, No Blood Culture | 2,679 | 157 (5.9) | — |  | — |  |
| Blood Culture(-) Suspected Co-infection | 1,286 | 302 (23) | 4.93 (4.02 - 6.12) |  | 4.28 (3.40 - 5.29) |  |
| Blood Culture(+) Confirmed Co-infection | 110 | 29 (26) | 5.74 (3.52 - 8.80) |  | 3.94 (2.23 - 6.49) |  |
| ^1^Unadjusted Odds Ratio 95% confidence interval (n=1000 bootstraps, median imputation and binarization for missing 24-hour post-admission SIRS score, k-nearest neighbors imputation for missing comorbidity history) | | | | | | |
| ^2^OR = Odds Ratio, CI = Confidence Interval | | | | | | |
| ^3^Wald Test | | | | | | |
| ^4^Adjusted Odds Ratio 95% confidence interval (n=1000 bootstraps, median imputation and binarization for missing 24-hour post-admission SIRS score, k-nearest neighbors imputation for missing comorbidity history) | | | | | | |

**eTable 27: Combined UAB and OLHS Cohorts In-Hospital Mortality Models (Sensitivity Testing with Imputed Data)**

| Characteristic | Encounters Total N | Deaths, N (% of Total) | In-Hospital Mortality Unadjusted OR (95% CI)^1,2^ | p-value^3^ | In-Hospital Mortality Adjusted OR (95% CI)^2,4^ | p-value^3^ |
| --- | --- | --- | --- | --- | --- | --- |
| Age Range |  |  |  | <0.001 |  | <0.001 |
| <65 years | 7,655 | 483 (6.3) | — |  | — |  |
| ≥65 years | 6,126 | 912 (15) | 2.60 (2.32 - 2.94) |  | 2.22 (1.98 - 2.51) |  |
| Diabetes History |  |  |  | <0.001 |  | 0.85 |
| No Diabetic History | 8,401 | 771 (9.2) | — |  | — |  |
| Diabetic History | 5,380 | 624 (12) | 1.30 (1.15 - 1.44) |  | 0.99 (0.88 - 1.11) |  |
| COPD History |  |  |  | <0.001 |  | 0.84 |
| No COPD History | 10,699 | 1017 (9.5) | — |  | — |  |
| COPD History | 3,082 | 378 (12) | 1.33 (1.16 - 1.51) |  | 0.99 (0.86 - 1.13) |  |
| Heart Failure or MI History |  |  |  | <0.001 |  | <0.001 |
| No Cardiac History | 10,669 | 887 (8.3) | — |  | — |  |
| Heart Failure or MI History | 3,112 | 508 (16) | 2.16 (1.92 - 2.42) |  | 1.76 (1.52 - 2.02) |  |
| Renal Disease History |  |  |  | <0.001 |  | 0.006 |
| No Renal History | 10,785 | 939 (8.7) | — |  | — |  |
| Renal Disease History | 2,996 | 456 (15) | 1.89 (1.67 - 2.11) |  | 1.22 (1.06 - 1.40) |  |
| Sex |  |  |  | <0.001 |  | <0.001 |
| Female | 6,963 | 581 (8.3) | — |  | — |  |
| Male | 6,818 | 814 (12) | 1.49 (1.32 - 1.67) |  | 1.43 (1.27 - 1.60) |  |
| First 24-hour SIRS Score |  |  |  | <0.001 |  | <0.001 |
| <2 SIRS Score | 5,194 | 336 (6.5) | — |  | — |  |
| ≥2 SIRS Score | 8,587 | 1059 (12) | 2.04 (1.81 - 2.32) |  | 1.78 (1.55 - 2.04) |  |
| COVID-19+ Co-infection Status |  |  |  | <0.001 |  | <0.001 |
| No Blood Culture, No Blood Culture | 7,080 | 380 (5.4) | — |  | — |  |
| Blood Culture(-) Suspected Co-infection | 6,351 | 934 (15) | 3.05 (2.72 - 3.45) |  | 2.49 (2.19 - 2.85) |  |
| Blood Culture(+) Confirmed Co-infection | 350 | 81 (23) | 5.31 (4.04 - 6.92) |  | 3.88 (2.92 - 5.10) |  |
| ^1^Unadjusted Odds Ratio 95% confidence interval (n=1000 bootstraps, median imputation and binarization for missing 24-hour post-admission SIRS score, k-nearest neighbors imputation for missing comorbidity history) | | | | | | |
| ^2^OR = Odds Ratio, CI = Confidence Interval | | | | | | |
| ^3^Wald Test | | | | | | |
| ^4^Adjusted Odds Ratio 95% confidence interval (n=1000 bootstraps, median imputation and binarization for missing 24-hour post-admission SIRS score, k-nearest neighbors imputation for missing comorbidity history) | | | | | | |

**Sensitivity Testing Section 3:**

**(Combined Reference Group**

**and**

**Data Imputation Testing)**

| **eTable 28: OLHS Cohort In-Hospital Mortality Models (Sensitivity Testing with Blood Culture(-) Suspected Co-infection as Reference and Imputed Data)** | | | | | | |
| --- | --- | --- | --- | --- | --- | --- |
| Characteristic | Encounters Total N | Deaths, N (% of Total) | In-Hospital Mortality Unadjusted OR (95% CI)^1,2^ | p-value^3^ | In-Hospital Mortality Adjusted OR (95% CI)^2,4^ | p-value^3^ |
| Age Range |  |  |  | <0.001 |  | <0.001 |
| <65 years | 2,559 | 216 (8.4) | — |  | — |  |
| ≥65 years | 2,746 | 468 (17) | 2.24 (1.89 - 2.67) |  | 2.19 (1.85 - 2.63) |  |
| Diabetes History |  |  |  | <0.001 |  | 0.51 |
| No Diabetic History | 3,417 | 401 (12) | — |  | — |  |
| Diabetic History | 1,888 | 283 (15) | 1.33 (1.12 - 1.57) |  | 1.06 (0.88 - 1.28) |  |
| COPD History |  |  |  | 0.003 |  | 0.79 |
| No COPD History | 3,989 | 483 (12) | — |  | — |  |
| COPD History | 1,316 | 201 (15) | 1.30 (1.09 - 1.55) |  | 1.03 (0.83 - 1.26) |  |
| Heart Failure or MI History |  |  |  | <0.001 |  | <0.001 |
| No Cardiac History | 4,146 | 461 (11) | — |  | — |  |
| Heart Failure or MI History | 1,159 | 223 (19) | 1.91 (1.60 - 2.26) |  | 1.65 (1.34 - 2.02) |  |
| Renal Disease History |  |  |  | <0.001 |  | 0.89 |
| No Renal History | 4,037 | 479 (12) | — |  | — |  |
| Renal Disease History | 1,268 | 205 (16) | 1.43 (1.20 - 1.71) |  | 1.02 (0.82 - 1.25) |  |
| Sex |  |  |  | <0.001 |  | <0.001 |
| Female | 2,538 | 287 (11) | — |  | — |  |
| Male | 2,767 | 397 (14) | 1.32 (1.13 - 1.55) |  | 1.37 (1.15 - 1.62) |  |
| First 24-hour SIRS Score |  |  |  | <0.001 |  | <0.001 |
| <2 SIRS Score | 1,136 | 85 (7.5) | — |  | — |  |
| ≥2 SIRS Score | 4,169 | 599 (14) | 2.08 (1.64 - 2.63) |  | 2.36 (1.87 - 2.99) |  |
| COVID-19+ Co-infection Status |  |  |  | <0.001 |  | <0.001 |
| Blood Culture(-) Suspected Co-infection | 5,065 | 632 (12) | — |  | — |  |
| Blood Culture(+) Confirmed Co-infection | 240 | 52 (22) | 1.92 (1.38 - 2.55) |  | 1.73 (1.25 - 2.42) |  |
| ^1^Unadjusted Odds Ratio 95% confidence interval (n=1000 bootstraps, median imputation and binarization for missing 24-hour post-admission SIRS score, k-nearest neighbors imputation for missing comorbidity history) | | | | | | |
| ^2^OR = Odds Ratio, CI = Confidence Interval | | | | | | |
| ^3^Wald Test | | | | | | |
| ^4^Adjusted Odds Ratio 95% confidence interval (n=1000 bootstraps, median imputation and binarization for missing 24-hour post-admission SIRS score, k-nearest neighbors imputation for missing comorbidity history) | | | | | | |

| **eTable 29: UAB Cohort In-Hospital Mortality Models (Sensitivity Testing with Blood Culture(-) Suspected Co-infection as Reference and Imputed Data)** | | | | | | |
| --- | --- | --- | --- | --- | --- | --- |
| Characteristic | Encounters Total N | Deaths, N (% of Total) | In-Hospital Mortality Unadjusted OR (95% CI)^1,2^ | p-value^3^ | In-Hospital Mortality Adjusted OR (95% CI)^2,4^ | p-value^3^ |
| Age Range |  |  |  | <0.001 |  | <0.001 |
| <65 years | 801 | 135 (17) | — |  | — |  |
| ≥65 years | 595 | 196 (33) | 2.42 (1.93 - 3.12) |  | 2.34 (1.78 - 3.06) |  |
| Diabetes History |  |  |  | 0.37 |  | 0.43 |
| No Diabetic History | 730 | 166 (23) | — |  | — |  |
| Diabetic History | 666 | 165 (25) | 1.13 (0.87 - 1.44) |  | 0.90 (0.66 - 1.16) |  |
| COPD History |  |  |  | 0.10 |  | 0.89 |
| No COPD History | 1,068 | 242 (23) | — |  | — |  |
| COPD History | 328 | 89 (27) | 1.28 (0.95 - 1.70) |  | 1.01 (0.73 - 1.41) |  |
| Heart Failure or MI History |  |  |  | <0.001 |  | <0.001 |
| No Cardiac History | 1,001 | 197 (20) | — |  | — |  |
| Heart Failure or MI History | 395 | 134 (34) | 2.08 (1.61 - 2.64) |  | 1.83 (1.34 - 2.49) |  |
| Renal Disease History |  |  |  | 0.005 |  | 0.44 |
| No Renal History | 988 | 214 (22) | — |  | — |  |
| Renal Disease History | 408 | 117 (29) | 1.45 (1.12 - 1.86) |  | 1.13 (0.83 - 1.52) |  |
| Sex |  |  |  | 0.009 |  | 0.019 |
| Female | 653 | 134 (21) | — |  | — |  |
| Male | 743 | 197 (27) | 1.41 (1.08 - 1.80) |  | 1.38 (1.04 - 1.79) |  |
| First 24-hour SIRS Score |  |  |  | 0.11 |  | 0.026 |
| <2 SIRS Score | 618 | 134 (22) | — |  | — |  |
| ≥2 SIRS Score | 778 | 197 (25) | 1.22 (0.96 - 1.58) |  | 1.36 (1.04 - 1.78) |  |
| COVID-19+ Co-infection Status |  |  |  | 0.50 |  | 0.85 |
| Blood Culture(-) Suspected Co-infection | 1,286 | 302 (23) | — |  | — |  |
| Blood Culture(+) Confirmed Co-infection | 110 | 29 (26) | 1.18 (0.72 - 1.77) |  | 0.96 (0.54 - 1.53) |  |
| ^1^Unadjusted Odds Ratio 95% confidence interval (n=1000 bootstraps, median imputation and binarization for missing 24-hour post-admission SIRS score, k-nearest neighbors imputation for missing comorbidity history) | | | | | | |
| ^2^OR = Odds Ratio, CI = Confidence Interval | | | | | | |
| ^3^Wald Test | | | | | | |
| ^4^Adjusted Odds Ratio 95% confidence interval (n=1000 bootstraps, median imputation and binarization for missing 24-hour post-admission SIRS score, k-nearest neighbors imputation for missing comorbidity history) | | | | | | |

| **eTable 30: Combined UAB and OLHS Cohorts In-Hospital Mortality Models (Sensitivity Testing with Blood Culture(-) Suspected Co-infection as Reference and Imputed Data)** | | | | | | |
| --- | --- | --- | --- | --- | --- | --- |
| Characteristic | Encounters Total N | Deaths, N (% of Total) | In-Hospital Mortality Unadjusted OR (95% CI)^1,2^ | p-value^3^ | In-Hospital Mortality Adjusted OR (95% CI)^2,4^ | p-value^3^ |
| Age Range |  |  |  | <0.001 |  | <0.001 |
| <65 years | 3,360 | 351 (10) | — |  | — |  |
| ≥65 years | 3,341 | 664 (20) | 2.13 (1.85 - 2.44) |  | 2.01 (1.74 - 2.34) |  |
| Diabetes History |  |  |  | 0.006 |  | 0.95 |
| No Diabetic History | 3,961 | 560 (14) | — |  | — |  |
| Diabetic History | 2,740 | 455 (17) | 1.21 (1.06 - 1.38) |  | 1.00 (0.87 - 1.16) |  |
| COPD History |  |  |  | <0.001 |  | 0.30 |
| No COPD History | 5,127 | 723 (14) | — |  | — |  |
| COPD History | 1,574 | 292 (19) | 1.38 (1.19 - 1.60) |  | 1.08 (0.91 - 1.28) |  |
| Heart Failure or MI History |  |  |  | <0.001 |  | <0.001 |
| No Cardiac History | 5,112 | 645 (13) | — |  | — |  |
| Heart Failure or MI History | 1,589 | 370 (23) | 2.10 (1.82 - 2.42) |  | 1.84 (1.56 - 2.19) |  |
| Renal Disease History |  |  |  | <0.001 |  | 0.53 |
| No Renal History | 5,021 | 689 (14) | — |  | — |  |
| Renal Disease History | 1,680 | 326 (19) | 1.52 (1.31 - 1.75) |  | 1.06 (0.89 - 1.25) |  |
| Sex |  |  |  | <0.001 |  | <0.001 |
| Female | 3,191 | 421 (13) | — |  | — |  |
| Male | 3,510 | 594 (17) | 1.34 (1.16 - 1.54) |  | 1.39 (1.20 - 1.60) |  |
| First 24-hour SIRS Score |  |  |  | <0.001 |  | <0.001 |
| <2 SIRS Score | 1,709 | 195 (11) | — |  | — |  |
| ≥2 SIRS Score | 4,992 | 820 (16) | 1.52 (1.30 - 1.81) |  | 1.71 (1.46 - 2.05) |  |
| COVID-19+ Co-infection Status |  |  |  | <0.001 |  | <0.001 |
| Blood Culture(-) Suspected Co-infection | 6,351 | 934 (15) | — |  | — |  |
| Blood Culture(+) Confirmed Co-infection | 350 | 81 (23) | 1.74 (1.34 - 2.22) |  | 1.57 (1.19 - 2.04) |  |
| ^1^Unadjusted Odds Ratio 95% confidence interval (n=1000 bootstraps, median imputation and binarization for missing 24-hour post-admission SIRS score, k-nearest neighbors imputation for missing comorbidity history) | | | | | | |
| ^2^OR = Odds Ratio, CI = Confidence Interval | | | | | | |
| ^3^Wald Test | | | | | | |
| ^4^Adjusted Odds Ratio 95% confidence interval (n=1000 bootstraps, median imputation and binarization for missing 24-hour post-admission SIRS score, k-nearest neighbors imputation for missing comorbidity history) | | | | | | |

**Sensitivity Testing Section 4:**

**(Pre-existing Conditions/Comorbidity History Testing)**

| **­eTable 31: Combined UAB and OLHS Cohorts In-Hospital Mortality Models (Sensitivity Testing with Hematologic Disease History)** | | | | | | |
| --- | --- | --- | --- | --- | --- | --- |
| Characteristic | Encounters Total N | Deaths, N (% of Total) | In-Hospital Mortality Unadjusted OR (95% CI)^1,2^ | p-value^3^ | In-Hospital Mortality Adjusted OR (95% CI)^2,4^ | p-value^3^ |
| Age Range |  |  |  | <0.001 |  | <0.001 |
| <65 years | 6,072 | 370 (6.1) | — |  | — |  |
| ≥65 years | 5,212 | 767 (15) | 2.65 (2.31 - 3.02) |  | 2.37 (2.08 - 2.75) |  |
| Diabetes History |  |  |  | <0.001 |  | 0.52 |
| No Diabetic History | 7,165 | 646 (9.0) | — |  | — |  |
| Diabetic History | 4,119 | 491 (12) | 1.37 (1.19 - 1.55) |  | 1.05 (0.91 - 1.20) |  |
| COPD History |  |  |  | 0.022 |  | 0.15 |
| No COPD History | 8,472 | 822 (9.7) | — |  | — |  |
| COPD History | 2,812 | 315 (11) | 1.17 (1.02 - 1.34) |  | 0.90 (0.77 - 1.04) |  |
| Heart Failure or MI History |  |  |  | <0.001 |  | <0.001 |
| No Cardiac History | 8,526 | 736 (8.6) | — |  | — |  |
| Heart Failure or MI History | 2,758 | 401 (15) | 1.79 (1.57 - 2.05) |  | 1.46 (1.25 - 1.71) |  |
| Renal Disease History |  |  |  | <0.001 |  | 0.46 |
| No Renal History | 8,540 | 764 (8.9) | — |  | — |  |
| Renal Disease History | 2,744 | 373 (14) | 1.60 (1.40 - 1.82) |  | 1.06 (0.90 - 1.24) |  |
| Sex |  |  |  | <0.001 |  | <0.001 |
| Female | 5,894 | 488 (8.3) | — |  | — |  |
| Male | 5,390 | 649 (12) | 1.52 (1.33 - 1.71) |  | 1.40 (1.22 - 1.59) |  |
| First 24-hour SIRS Score |  |  |  | <0.001 |  | <0.001 |
| <2 SIRS Score | 4,369 | 267 (6.1) | — |  | — |  |
| ≥2 SIRS Score | 6,915 | 870 (13) | 2.22 (1.93 - 2.55) |  | 1.91 (1.66 - 2.23) |  |
| Hematologic Disease History^5^ |  |  |  | <0.001 |  | <0.001 |
| No Hematologic Disease History | 9,901 | 931 (9.4) | — |  | — |  |
| Hematologic Disease History | 1,383 | 206 (15) | 1.69 (1.42 - 1.97) |  | 1.39 (1.17 - 1.66) |  |
| COVID-19+ Co-infection Status |  |  |  | <0.001 |  | <0.001 |
| No Blood Culture, No Co-infection | 5,622 | 294 (5.2) | — |  | — |  |
| Blood Culture(-) Suspected Co-infection | 5,359 | 774 (14) | 3.06 (2.66 - 3.53) |  | 2.43 (2.09 - 2.81) |  |
| Blood Culture(+) Confirmed Co-infection | 303 | 69 (23) | 5.35 (3.87 - 7.03) |  | 3.85 (2.74 - 5.16) |  |
| ^1^Unadjusted Odds Ratio 95% confidence interval (n=1000 bootstraps) | | | | | | |
| ^2^OR = Odds Ratio, CI = Confidence Interval | | | | | | |
| ^3^Wald Test | | | | | | |
| ^4^Adjusted Odds Ratio 95% confidence interval (n=1000 bootstraps)  ^5^Hemtologic disease history was derived from ICD-9/10 (International Code of Disease 9^th^/10^th^ version) billing code evidence for any of the following:   - HIV/AIDS (ICD-9: 042.x-0.44.x; ICD-10: B20.x-B.22.x, B24.x) - Lymphoma (ICD-9: x–202.3x, 202.5–203.0, 203.8, 238.6, 273.3, V10.71, V10.72, V10.79; ICD-10: C81.x–C85.x, C88.x, C96.x, C90.0, C90.2) - Select Coagulopathies (ICD-9: 286.x, 287.1, 287.3–287.5; ICD-10: D65–D68.x, D69.1, D69.3– D69.6) | | | | | | |
|  | | | | | | |

| **eTable 32: Combined UAB and OLHS Cohorts In-Hospital Mortality Models (Sensitivity Testing with Hematologic Disease History and Blood Culture(-) Suspected Co-infection as Reference)** | | | | | | |
| --- | --- | --- | --- | --- | --- | --- |
| Characteristic | Encounters Total N | Deaths, N (% of Total) | In-Hospital Mortality Unadjusted OR (95% CI)^1,2^ | p-value^3^ | In-Hospital Mortality Adjusted OR (95% CI)^2,4^ | p-value^3^ |
| Age Range |  |  |  | <0.001 |  | <0.001 |
| <65 years | 2,716 | 273 (10) | — |  | — |  |
| ≥65 years | 2,946 | 570 (19) | 2.15 (1.84 - 2.53) |  | 2.13 (1.84 - 2.50) |  |
| Diabetes History |  |  |  | 0.005 |  | 0.62 |
| No Diabetic History | 3,423 | 473 (14) | — |  | — |  |
| Diabetic History | 2,239 | 370 (17) | 1.23 (1.06 - 1.42) |  | 1.04 (0.89 - 1.22) |  |
| COPD History |  |  |  | 0.006 |  | 0.98 |
| No COPD History | 4,199 | 593 (14) | — |  | — |  |
| COPD History | 1,463 | 250 (17) | 1.25 (1.06 - 1.45) |  | 0.99 (0.84 - 1.17) |  |
| Heart Failure or MI History |  |  |  | <0.001 |  | <0.001 |
| No Cardiac History | 4,224 | 539 (13) | — |  | — |  |
| Heart Failure or MI History | 1,438 | 304 (21) | 1.83 (1.59 - 2.12) |  | 1.56 (1.31 - 1.86) |  |
| Renal Disease History |  |  |  | <0.001 |  | 0.87 |
| No Renal History | 4,100 | 562 (14) | — |  | — |  |
| Renal Disease History | 1,562 | 281 (18) | 1.39 (1.17 - 1.61) |  | 0.98 (0.82 - 1.18) |  |
| Sex |  |  |  | <0.001 |  | <0.001 |
| Female | 2,784 | 357 (13) | — |  | — |  |
| Male | 2,878 | 486 (17) | 1.38 (1.19 - 1.59) |  | 1.39 (1.20 - 1.61) |  |
| First 24-hour SIRS Score |  |  |  | <0.001 |  | <0.001 |
| <2 SIRS Score | 1,484 | 155 (10) | — |  | — |  |
| ≥2 SIRS Score | 4,178 | 688 (16) | 1.69 (1.42 - 2.05) |  | 1.92 (1.60 - 2.31) |  |
| Hematologic Disease History^5^ |  |  |  | <0.001 |  | <0.001 |
| No Hematologic Disease History | 4,897 | 682 (14) | — |  | — |  |
| Hematologic Disease History | 765 | 161 (21) | 1.63 (1.37 - 1.97) |  | 1.45 (1.16 - 1.76) |  |
| COVID-19+ Co-infection Status |  |  |  | <0.001 |  | 0.002 |
| Blood Culture(-) Suspected Co-infection | 5,359 | 774 (14) | — |  | — |  |
| Blood Culture(+) Confirmed Co-infection | 303 | 69 (23) | 1.76 (1.29 - 2.30) |  | 1.55 (1.12 - 2.08) |  |
| ^1^Unadjusted Odds Ratio 95% confidence interval (n=1000 bootstraps) | | | | | | |
| ^2^OR = Odds Ratio, CI = Confidence Interval | | | | | | |
| ^3^Wald Test | | | | | | |
| ^4^Adjusted Odds Ratio 95% confidence interval (n=1000 bootstraps)  ^5^Hemtologic disease history was derived from ICD-9/10 (International Code of Disease 9^th^/10^th^ version) billing code evidence for any of the following:   - HIV/AIDS (ICD-9: 042.x-0.44.x; ICD-10: B20.x-B.22.x, B24.x) - Lymphoma (ICD-9: x–202.3x, 202.5–203.0, 203.8, 238.6, 273.3, V10.71, V10.72, V10.79; ICD-10: C81.x–C85.x, C88.x, C96.x, C90.0, C90.2) - Select Coagulopathies (ICD-9: 286.x, 287.1, 287.3–287.5; ICD-10: D65–D68.x, D69.1, D69.3– D69.6) | | | | | | |

| **eTable 33: UAB Cohort In-Hospital Mortality Models (Sensitivity Testing with Solid Organ Transplant Status)** | | | | | | |
| --- | --- | --- | --- | --- | --- | --- |
| Characteristic | Encounters Total N | Deaths, N (% of Total) | In-Hospital Mortality Unadjusted OR (95% CI)^1,2^ | p-value^3^ | In-Hospital Mortality Adjusted OR (95% CI)^2,4^ | p-value^3^ |
| Age Range |  |  |  | <0.001 |  | <0.001 |
| <65 years | 1,704 | 116 (6.8) | — |  | — |  |
| ≥65 years | 1,168 | 183 (16) | 2.55 (2.02 - 3.28) |  | 2.44 (1.87 - 3.18) |  |
| Diabetes History |  |  |  | <0.001 |  | 0.69 |
| No Diabetic History | 1,700 | 149 (8.8) | — |  | — |  |
| Diabetic History | 1,172 | 150 (13) | 1.54 (1.19 - 1.93) |  | 1.06 (0.82 - 1.36) |  |
| COPD History |  |  |  | 0.50 |  | 0.16 |
| No COPD History | 2,084 | 212 (10) | — |  | — |  |
| COPD History | 788 | 87 (11) | 1.10 (0.85 - 1.45) |  | 0.79 (0.60 - 1.07) |  |
| Heart Failure or MI History |  |  |  | <0.001 |  | 0.003 |
| No Cardiac History | 2,068 | 169 (8.2) | — |  | — |  |
| Heart Failure or MI History | 804 | 130 (16) | 2.18 (1.71 - 2.73) |  | 1.56 (1.13 - 2.07) |  |
| Renal Disease History |  |  |  | <0.001 |  | 0.044 |
| No Renal History | 2,113 | 177 (8.4) | — |  | — |  |
| Renal Disease History | 759 | 122 (16) | 2.08 (1.60 - 2.67) |  | 1.39 (1.01 - 1.89) |  |
| Sex |  |  |  | <0.001 |  | 0.011 |
| Female | 1,551 | 128 (8.3) | — |  | — |  |
| Male | 1,321 | 171 (13) | 1.64 (1.31 - 2.12) |  | 1.40 (1.07 - 1.83) |  |
| First 24-hour SIRS Score |  |  |  | <0.001 |  | <0.001 |
| <2 SIRS Score | 1,802 | 139 (7.7) | — |  | — |  |
| ≥2 SIRS Score | 1,070 | 160 (15) | 2.10 (1.69 - 2.62) |  | 1.71 (1.33 - 2.22) |  |
| Solid Organ Transplant Status |  |  |  | 0.008 |  | 0.71 |
| No Solid Organ Transplant | 2,649 | 264 (10.0) | — |  | — |  |
| Solid Organ Transplant Recipient | 223 | 35 (16) | 1.68 (1.12 - 2.33) |  | 1.08 (0.68 - 1.68) |  |
| COVID-19+ Co-infection Status |  |  |  | <0.001 |  | <0.001 |
| No Blood Culture, No Co-infection | 1,896 | 102 (5.4) | — |  | — |  |
| Blood Culture(-) Suspected Co-infection | 884 | 172 (19) | 4.25 (3.28 - 5.51) |  | 3.44 (2.68 - 4.56) |  |
| Blood Culture(+) Confirmed Co-infection | 92 | 25 (27) | 6.64 (4.05 - 10.7) |  | 4.02 (2.16 - 6.74) |  |
| ^1^Unadjusted Odds Ratio 95% confidence interval (n=1000 bootstraps) | | | | | | |
| ^2^OR = Odds Ratio, CI = Confidence Interval | | | | | | |
| ^3^Wald Test | | | | | | |
| ^4^Adjusted Odds Ratio 95% confidence interval (n=1000 bootstraps) | | | | | | |

| **eTable 34: UAB Cohort In-Hospital Mortality Models (Sensitivity Testing with Solid Organ Transplant Status and Blood Culture(-) Suspected Co-infection as Reference)** | | | | | | |
| --- | --- | --- | --- | --- | --- | --- |
| Characteristic | Encounters Total N | Deaths, N (% of Total) | In-Hospital Mortality Unadjusted OR (95% CI)^1,2^ | p-value^3^ | In-Hospital Mortality Adjusted OR (95% CI)^2,4^ | p-value^3^ |
| Age Range |  |  |  | <0.001 |  | <0.001 |
| <65 years | 547 | 76 (14) | — |  | — |  |
| ≥65 years | 429 | 121 (28) | 2.46 (1.79 - 3.46) |  | 2.48 (1.75 - 3.58) |  |
| Diabetes History |  |  |  | 0.056 |  | 0.64 |
| No Diabetic History | 525 | 94 (18) | — |  | — |  |
| Diabetic History | 451 | 103 (23) | 1.37 (0.98 - 1.89) |  | 1.09 (0.77 - 1.54) |  |
| COPD History |  |  |  | 0.48 |  | 0.65 |
| No COPD History | 689 | 135 (20) | — |  | — |  |
| COPD History | 287 | 62 (22) | 1.13 (0.79 - 1.54) |  | 0.92 (0.63 - 1.32) |  |
| Heart Failure or MI History |  |  |  | <0.001 |  | 0.021 |
| No Cardiac History | 648 | 107 (17) | — |  | — |  |
| Heart Failure or MI History | 328 | 90 (27) | 1.91 (1.40 - 2.59) |  | 1.55 (1.08 - 2.25) |  |
| Renal Disease History |  |  |  | 0.005 |  | 0.36 |
| No Renal History | 639 | 112 (18) | — |  | — |  |
| Renal Disease History | 337 | 85 (25) | 1.60 (1.13 - 2.19) |  | 1.21 (0.79 - 1.80) |  |
| Sex |  |  |  | 0.040 |  | 0.11 |
| Female | 475 | 83 (17) | — |  | — |  |
| Male | 501 | 114 (23) | 1.41 (1.01 - 1.91) |  | 1.32 (0.94 - 1.82) |  |
| First 24-hour SIRS Score |  |  |  | 0.040 |  | 0.004 |
| <2 SIRS Score | 430 | 74 (17) | — |  | — |  |
| ≥2 SIRS Score | 546 | 123 (23) | 1.38 (1.04 - 1.90) |  | 1.66 (1.21 - 2.34) |  |
| Solid Organ Transplant Status |  |  |  | 0.26 |  | 0.40 |
| No Solid Organ Transplant | 860 | 169 (20) | — |  | — |  |
| Solid Organ Transplant Recipient | 116 | 28 (24) | 1.28 (0.79 - 1.98) |  | 1.26 (0.72 - 2.00) |  |
| COVID-19+ Co-infection Status |  |  |  | 0.081 |  | 0.47 |
| Blood Culture(-) Suspected Co-infection | 884 | 172 (19) | — |  | — |  |
| Blood Culture(+) Confirmed Co-infection | 92 | 25 (27) | 1.55 (0.93 - 2.46) |  | 1.20 (0.68 - 1.97) |  |
| ^1^Unadjusted Odds Ratio 95% confidence interval (n=1000 bootstraps) | | | | | | |
| ^2^OR = Odds Ratio, CI = Confidence Interval | | | | | | |
| ^3^Wald Test | | | | | | |
| ^4^Adjusted Odds Ratio 95% confidence interval (n=1000 bootstraps) | | | | | | |
